# Federated Learning for Predicting Major Postoperative Complications

**DOI:** 10.1097/AS9.0000000000000573

**Published:** 2025-05-02

**Authors:** Yuanfang Ren, Yonggi Park, Benjamin Shickel, Ziyuan Guan, Ayush Patel, Yingbo Ma, Zhenhong Hu, Jeremy A. Balch, Tyler J. Loftus, Parisa Rashidi, Tezcan Ozrazgat-Baslanti, Azra Bihorac

**Affiliations:** From the * Intelligent Clinical Care Center, University of Florida, Gainesville, FL; † Division of Nephrology, Hypertension, and Renal Transplantation, Department of Medicine, University of Florida, Gainesville, FL; ‡ Department of Surgery, University of Florida, Gainesville, FL; § Department of Biomedical Engineering, University of Florida, Gainesville, FL.

**Keywords:** data privacy, electronic health records, federated learning, major surgery, postoperative complications

## Abstract

**Objective:**

To develop a robust model to accurately predict the risk of postoperative complications using clinical data from multiple institutions while ensuring data privacy.

**Background:**

Building accurate, artificial intelligence models to predict postoperative complications relies on accessibility of large-scale and diverse datasets, often restricted by privacy concerns.

**Methods:**

This retrospective cohort study includes adult patients admitted to University of Florida Health (UFH) hospitals in Gainesville (GNV) (n = 79,850) and Jacksonville (JAX) (n = 28,636) for all inpatient major surgical procedures. We developed federated learning models to predict 9 major postoperative complications and compared them with both local models trained on a single site and central models trained on a pooled dataset from 2 hospitals.

**Results:**

Our best-federated learning models using preoperative features achieved the area under the receiver operating characteristics curve values with 95% confidence interval (CI) ranging from 0.80 (95% CI, 0.79–0.80) for wound complications to 0.90 (95% CI, 0.90–0.91) for prolonged intensive care unit (ICU) stay at UFH GNV. At UFH JAX, these values ranged from 0.71 (95% CI, 0.70–0.72) for wound complications to 0.90 (95% CI, 0.88–0.92) for in-hospital mortality. Federated learning models achieved comparable discrimination to central models for all outcomes, except prolonged ICU stay, where the performance of the federated learning model was slightly better at UFH GNV and slightly worse at UFH JAX. Our federated learning models obtained comparable performance to the best local models.

**Conclusions:**

We show federated learning to be a useful tool to train robust postoperative outcome prediction models from large-scale data across 2 hospitals.

## INTRODUCTION

In the United States, over 15 million major inpatient surgeries are performed annually and at least 150,000 patients die within 30 days after surgery due to postoperative complications.^1,2^ Postoperative complications occur in up to 32% of surgeries, ranging from minor wound complications to mortality.^3^ Beyond the immediate health impacts on patients, these complications also incur substantial financial costs, both for individuals and healthcare institutions.^4,5^ The accurate prediction of postoperative complication risk during the preoperative period is crucial, as it allows for the identification of patients who would benefit from postoperative triage to an intensive care unit (ICU) as well as more informed decision-making by patients and their healthcare providers.

Assessing surgical risk necessitates the timely and accurate synthesis of vast amount of clinical data. The integration of artificial intelligence (AI) with electronic health records (EHR) data has facilitated data digitization, enabling the increased utilization of machine-learning tools for risk surveillance and diagnosis.^6,7^ While studies using data from single hospitals have provided insights into surgical risk estimation, there is a need for broader representation from diverse populations to enhance generalizability.^3,7–9^ Even with the option of centralized data pooling from multiple centers, there still exists concern for privacy violations and vulnerable data leakage.^10–13^ Federated learning offers a potential solution. This collaborative and decentralized machine-learning approach involves maintaining a deep-learning model within a central server, while the training process is distributed across medical centers.^13,14^ Each center develops an individualized model based on its unique patient data, and the central server updates the global model following predefined criteria, selectively incorporating valuable feedback.^14^ This preserves patient data privacy and security standards.^9–14^ Despite successful applications of federated learning in predicting outcomes such as acute kidney injury (AKI) stage, adverse drug reactions, hospitalizations, and COVID-19 mortality, its application for predicting surgical postoperative outcomes has not been explored.^9,10,13–15^

Our objective was to develop a robust federated learning model to accurately predict the risk of postoperative complications using EHR data from 2 academic hospitals. Our hypothesis was that our federated learning model trained on distributed data would have comparable performance to a model trained on pooled data while simultaneously preserving data privacy and security.

## METHODS

### Data Source and Participants

Using the University of Florida Health (UFH) Integrated Repository as an honest broker for data deidentification, we gathered 2 single-center, longitudinal EHR datasets for all adult patients who were admitted to UFH Gainesville (GNV) and Jacksonville (JAX) for any type of inpatient major surgical procedure between January 1, 2012, and May 1, 2021. We excluded minor procedures performed for controlling pain (nerve block performed outside the operating room), gastrointestinal-related minor surgeries (endoscopic procedures performed outside the operating room), and organ donation surgeries for patients who donated organs before death. Detailed inclusion and exclusion criteria used to identify encounters involving completed inpatient major surgical procedures are shown in Supplemental Figure 1, see https://links.lww.com/AOSO/A493. When a patient had multiple surgeries during one admission, only the first surgery was used in the analysis. The final retrospective UFH GNV cohort consisted of 62,827 patients undergoing 79,850 surgeries and the retrospective UFH JAX cohort consisted of 23,563 patients undergoing 28,636 surgeries.

Each dataset includes demographic information, vital signs, laboratory values, medications, diagnoses, and procedures for all index admissions as well as admissions within 12 months before index admissions. This study was approved by the University of Florida Institutional Review Board and Privacy Office (IRB#201600223, IRB#201600262) as an exempt study with a waiver of informed consent.

### Study Design

**FIGURE 1.**
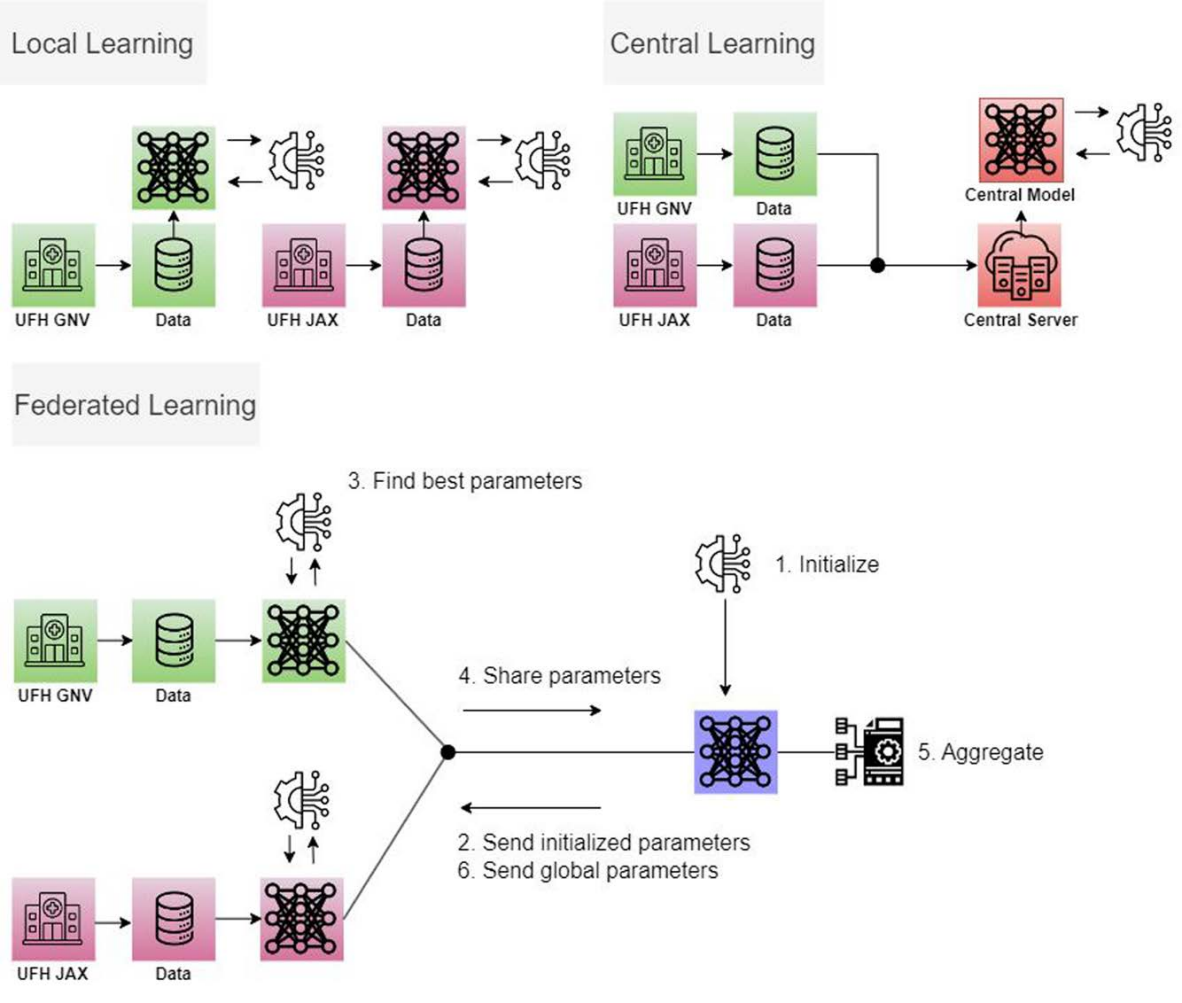
Difference in learning paradigms of local learning, central learning, and federated learning. Local learning trains the model on data from a single center. Central learning trains the model on centralized data pooled from multiple centers. Federated learning trains each local model at each center and aggregates the respective computed model weights at a server without the need to exchange the actual data.

We followed the guidelines given in Transparent Reporting of a multivariable prediction model for Individual Prognosis or Diagnosis (TRIPOD)^16^ and Leisman et al^17^ under the type 2b analysis category. We chronologically split each dataset by surgery start dates into 3 cohorts: training (63% of observations, n = 51,953 surgeries for UFH GNV and n = 18,502 surgeries for UFH JAX), validation (7% of observations, n = 5565 surgeries for UFH GNV and n = 2023 surgeries for UFH JAX), and test (30% of observations, n = 22,332 surgeries for UFH GNV and n = 8111 surgeries for UFH JAX) cohorts to mitigate potential adverse effects of dataset drift due to changes in clinical practice or patient populations over time. All data from each patient were assigned to only one cohort. We developed the model using a training cohort and employed 3 distinct learning paradigms: local learning, central learning, and federated learning (Fig. 1). Local learning trains the model on data from a single center, while central learning trains the model on centralized data pooled from multiple centers. Federated learning trains each local model at each center and aggregates the respective computed model weights at a server without the need to exchange the actual data. We utilized a validation cohort to tune the hyperparameters and select the model. We validated the performance of models on the test cohort.

### Outcome

We have developed and implemented an array of AI algorithms for computable phenotyping and dynamic perioperative risk assessment for 9 postoperative complications including prolonged (>48 hours) ICU stay and mechanical ventilation (MV), neurological complications including delirium, cardiovascular complications, AKI, venous thromboembolism (VTE), sepsis, wound complications that include infectious and mechanical wound complications, and in-hospital mortality.^3,8,18^ We used the exact dates to calculate the duration of MV and ICU stay. We defined AKI using KDIGO consensus criteria^19,20^ while a set of previously described criteria was applied to annotate the remaining complications.^21,22^ The algorithm also calculates risk probabilities for in-hospital mortality, where the date of death was determined using hospital records and the search of the Social Security Death Index.^23^

### Predictors

For each patient, we considered all potential predictors available in the harmonized dataset. We identified 402 preoperative features, including demographic, socioeconomic, admission information (eg., admission source), planned procedure and provider information, comorbidity, medications, and laboratory measurements. Supplemental Table 1, see https://links.lww.com/AOSO/A493 provides a comprehensive list of all preoperative features along with their distribution across 2 hospitals. We derived patient comorbidities and calculated the composite Charlson Comorbidity Index using up to 50 International Classification of Disease codes from all available historical diagnosis codes.^24–26^ We extracted medications dispensed in the 1 year before the scheduled surgery day using RxNorm data categorized into drug classes based on the United States Department of Veterans Affairs National Drug File Reference Terminology.^27^ We extracted laboratory values measured 1 year before surgery using Logical Observation Identifiers Names and Codes and calculated derived features, including the count, mean, variance, and minimum and maximum values. We also identified 9 intraoperative time series data including systolic blood pressure, diastolic blood pressure, mean arterial pressure, heart rate, temperature, end-tidal carbon dioxide, peripheral capillary oxygen saturation, peak inspiratory pressure, respiratory rate, and minimum alveolar concentration. Supplemental Figure 2, see https://links.lww.com/AOSO/A493 presents the distribution of these intraoperative time series data across 2 centers. For continuous preoperative variables and intraoperative time series variables, we also created “presence” features to enable our model to distinguish if a given value was observed or imputed, acknowledging that the patterns of missingness might be informative.

### Data Preprocessing

We processed feature variables to remove outliers, impute missingness, and standardize the values.^7,28^ A set of automatic rules was used for the removal of outliers that were considered unreasonable observations by medical experts. For continuous variables, observations in the top and bottom 1% of the distribution were considered as outliers, and they were imputed with a random number generated from the range between 0.5th and 5th percentiles and between 95th and 99.5th percentiles, respectively. All missing observations were imputed as follows. For nominal variables with missing entries, a distinct “missing” category was created. For continuous variables, the median value from the training cohort for a given variable was used for imputation. Intraoperative time series data were resampled at 1-minute intervals. Missing time series data were imputed by linear interpolation. If an entire time series variable was missing, the median values from the training cohort were used for imputation. We standardized the continuous variables using standard normalization techniques. For local learning and federated learning, the entire data preprocessing process was independently carried out at each center. For central learning, the process was conducted with pooled data from 2 centers.

### Model Architecture

Following the model architecture in the previously described study,^7^ we developed 2 deep-learning models to assess the risk of postoperative complications: a preoperative model and a perioperative model. The preoperative model utilized only preoperative features, whereas the perioperative model was developed using a combination of both preoperative and intraoperative features.

The perioperative model consisted of 2 submodels: one for processing preoperative features and another for intraoperative features. In the preoperative submodel, features were categorized into continuous, binary, and high-cardinality types and then input into respective neural networks tailored for each variable type. The network architecture for continuous and binary features consisted of fully connected layers, while high-cardinality features involved embedding layers followed by fully connected layers. The latent representation from 3 neural networks was combined through a fully connected layer. For the intraoperative submodel, a bidirectional recurrent neural network equipped with gated recurrent units and an attention mechanism was employed to process intraoperative multivariate time series data. The final patient representation was derived by integrating the outputs from both submodels through another fully connected layer. This integrated output was then passed into 9 distinct branches, each corresponding to 1 of the 9 outcomes, to calculate the risk probability. Within each branch, there was an outcome-specific fully connected layer followed by a sigmoid activation function, generating a score for each outcome. This score was interpreted as the likelihood of a preoperative patient developing a specific postoperative complication. For a more detailed description of the model, we refer readers to Shickel et al^7^.

The preoperative model shared a similar architecture with a perioperative model but differed in that it did not include the intraoperative submodel. We trained these 2 models using 3 learning paradigms: local learning, central learning, and federated learning. We used 3 federated learning algorithms including FedAvg,^29^ FedProx,^30^ and SCAFFOLD.^31^ FedAvg aggregates model updates from multiple clients using a central server and is a popular federated learning approach for its simplicity, scalability, and efficiency in communication. Despite these strengths, it encounters challenges pertaining to data heterogeneity and potential security vulnerabilities.^30^ FedProx extends the FedAvg algorithm by adding a regularization term to the loss function, which encourages model similarity across clients, thereby addressing the challenges posed by data and model heterogeneity.^30^ SCAFFOLD addresses the problem of nonindependently and identically distributed (non-IID) data in federated learning, but it comes with trade-off of higher computational and communication demands.^31^

### Statistical Analysis

We evaluated the robustness of models by (1) performing subgroup analysis based on sex (female vs male), race (African American vs non-African American), age (age ≤65 vs >65 years old), and surgery type^3^ (cardiothoracic surgery, noncardiac general surgery, neurological surgery, specialty surgery, and other surgery) and (2) conducting a sensitivity analysis of undersampling the data from the UFH GNV center to equalize the sample sizes between the 2 centers. We repeated the sampling experiment 10 times to enhance the reliability of the experiment’s findings.

We assessed each model’s discrimination using the area under the receiver operating characteristic curve (AUROC) and the area under the precision-recall curve (AUPRC). To obtain a 95% confidence interval (CI) for all performance metrics, we employed the 1000-sample bootstrap method. For comparing clinical characteristics and outcomes of patients, we utilized the χ2 test for categorical variables and the Kruskal–Wallis test for continuous variables. The threshold for statistical significance was set at less than 0.05 for 2-sided tests. We adjusted *P* values for the family-wise error rate resulting from multiple comparisons using the Bonferroni correction. We determined the statistically significant difference between AUROCs using DeLong test. Data analysis was conducted using Python software of version 3.9, R software of version 4.3.3, and NVFlare of version 2.3.

## RESULTS

### Patient Baseline Characteristics and Outcomes

Among 39,582 adult patients who underwent 51,953 major inpatient surgical procedures in the UFH training cohort, the mean (standard deviation, SD) age was 57 (17) years; 19,698 patients (50%) were female, and 19,884 (50%) were male (Table 1); 30,947 patients (78%) were White and 5474 (14%) were African American. The UFH JAX training cohort contained 18,502 inpatient surgical procedures involving 14,846 patients and the demographic characteristics were significantly different from the UFH GNV training cohort (younger patients with a mean [SD] age, 52 [17] years; 7204 [49%] were females and 7642 [51%] were males; 8675 [59%] were White and 5158 [35%] were African American).

**TABLE 1.** Patient Characteristics in Training Cohorts

Variables	UFH GNV	UFH JAX	*P* value
Number of patients, n	39,582	14,846	
Number of surgical procedures, n	51,953	18,502	
Age in years, mean (SD)*	57 (17)	52 (17)	<0.001
Sex, n (%)*			
Male	19,884 (50)	7642 (51)	<0.001
Female	19,698 (50)	7204 (49)	<0.001
Race, n (%)*†			
White	30,947 (78)	8675 (59)	<0.001
African American	5474 (14)	5158 (35)	<0.001
Other‡	2521 (6)	952 (6)	0.32
Missing	640 (2)	61 (0)	<0.001
Ethnicity, n (%)*†			
Non-Hispanic	37,165 (94)	14,079 (95)	<0.001
Hispanic	1707 (4)	676 (5)	0.96
Missing	710 (2)	91 (0)	<0.001
Marital status, n (%)*			
Married	19,182 (48)	5055 (34)	<0.001
Single	11,769 (30)	5392 (36)	<0.001
Divorced	6084 (15)	3875 (26)	<0.001
Missing	2547 (7)	524 (4)	<0.001
Insurance, n (%)*			
Medicare	17,767 (45)	4408 (30)	<0.001
Private	12,311 (31)	4308 (29)	<0.001
Medicaid	6296 (16)	5908 (40)	<0.001
Uninsured	3208 (8)	222 (1)	<0.001
Complications, n (%)§			
Prolonged ICU stay	15,049 (29)	4372 (24)	<0.001
Sepsis	3958 (8)	1601 (9)	<0.001
Cardiovascular complications	6419 (12)	2104 (11)	<0.001
Venous thromboembolism	2483 (5)	721 (4)	<0.001
Prolonged mechanical ventilation	4667 (9)	1492 (8)	<0.001
Neurological complications including delirium	8873 (17)	2199 (12)	<0.001
Wound complications	8088 (16)	2594 (14)	<0.001
Acute kidney injury	7924 (15)	2577 (14)	<0.001
In-hospital mortality	952 (2)	329 (2)	0.66

* Data were reported based on values calculated at the latest hospital admission.

† Race and ethnicity were self-reported.

‡ Other races include American Indian or Alaska Native, Asian, Native Hawaiian or Pacific Islander, and multiracial.

§ Data were reported based on postoperative complication status for each surgical procedure. When a patient had multiple surgeries during one admission, only the first surgery was used in the analysis.

Nearly each major surgical service was adequately represented with a notable lack of gastrointestinal and surgical oncology procedures at UFH JAX (Supplemental Table 1, see https://links.lww.com/AOSO/A493). The prevalence of postoperative complications in the UFH GNV training cohort was 15% for AKI, 12% for cardiovascular complications, 17% for neurological complications including delirium, 29% for prolonged ICU stay, 9% for prolonged MV, 8% for sepsis, 5% for VTE, 16% for wound complications, and 2% for in-hospital mortality. Compared with UFH GNV training cohort, UFH JAX training cohort had similar complication prevalence, except for neurological complications including delirium (12% vs 17%), prolonged ICU stay (24% vs 29%), and wound complications (14% vs 16%). There was also some variation in complication prevalence between the training and test cohorts at each center (Supplemental Table 2, see https://links.lww.com/AOSO/A493). For example, the prevalence of all complications except in-hospital mortality in UFH GNV training cohort was significantly different from that in UFH GNV test cohort. At UFH JAX center, the prevalence of complications including cardiovascular complications, prolonged MV, VTE, and in-hospital mortality was significantly different between training and test cohorts.

The distribution of features used for model development was significantly different between the 2 centers (Supplemental Table 1 and Supplemental Figure 2, see https://links.lww.com/AOSO/A493). Additional details of patient clinical characteristics, complication prevalence, and distribution of features are shown in Table 1 and Supplemental Tables 1 and 2, see https://links.lww.com/AOSO/A493.

### Comparison Between Central Learning and Federated Learning Models

We evaluated the performance of central learning models and federated learning models by calculating AUROC and AUPRC values (Tables 2–5). In the preoperative federated learning models, AUROC values ranged from 0.79 (95% CI, 0.78–0.80) for wound complications to 0.90 (95% CI, 0.90–0.91) for prolonged ICU stay at UFH GNV center (Table 2). At UFH JAX center, these values ranged from 0.71 (95% CI, 0.70–0.73) for wound complications to 0.90 (95% CI, 0.87–0.92) for in-hospital mortality (Table 3). In the perioperative federated learning models, AUROC values ranged from 0.81 (95% CI, 0.80–0.81) for wound complications to 0.92 (95% CI, 0.92–0.92) for prolonged ICU stay at UFH GNV center (Table 2). At UFH JAX center, these values ranged from 0.73 (95% CI, 0.72–0.75) for wound complications to 0.93 (95% CI, 0.91–0.95) for in-hospital mortality (Table 3). All perioperative models outperformed preoperative models in both sites in terms of AUROC, especially for cardiovascular complications (UFH GNV: 0.85 vs 0.82; UFH JAX: 0.84 vs 0.79). Federated learning models achieved comparable AUROC performance to central learning models for all outcomes, except prolonged ICU stay, where the performance of federated learning models was slightly higher than central learning models (preoperative models: 0.90 [95% CI, 0.90–0.91] vs 0.89 [95% CI, 0.89–0.90]; perioperative models: 0.92 [95% CI, 0.92–0.92] vs 0.91 [95% CI, 0.91–0.92]) at UFH GNV center, but slightly lower at UFH JAX center (preoperative models: 0.87 [95% CI, 0.86–0.88] vs 0.89 [95% CI, 0.89–0.90]; perioperative models: 0.89 [95% CI, 0.88–0.90] vs 0.91 [95% CI, 0.90–0.91]). Federated learning models achieved comparable AUPRC performance to the central learning model at UFH GNV center (Table 4) while yielding lower discrimination at UFH JAX center for the majority of the complications (Table 5). Three federated learning models had similar performance and the SCAFFOLD model achieved the best overall performance.

**TABLE 2.** Comparison of AUROC With 95% Confidence Interval for Central Learning and Federated Learning Models in the UFH GNV Cohort

Outcome	Period	CL	FedAvg	FedProx	SCAFFOLD
Prolonged ICU stay	Preop	0.89 (0.89–0.90)	**0.90 (0.90–0.90**)*	**0.90 (0.90–0.91**)*	**0.90 (0.90–0.91**)*
	Periop	0.91 (0.91–0.92)	**0.92 (0.92–0.92**)*	**0.92 (0.92–0.92**)*	**0.92 (0.92–0.92**)*
Sepsis	Preop	0.88 (0.87–0.88)	0.88 (0.87–0.88)	0.88 (0.87–0.89)	0.88 (0.87–0.88)
	Periop	0.89 (0.88–0.89)	0.89 (0.88–0.89)	0.89 (0.88–0.89)	0.89 (0.88–0.89)
Cardiovascular complication	Preop	0.82 (0.81–0.82)	0.82 (0.81–0.82)	0.82 (0.81–0.83)	0.82 (0.81–0.82)
	Periop	0.85 (0.84–0.85)	0.85 (0.85–0.86)	0.85 (0.85–0.86)	0.85 (0.84–0.86)
Venous thromboembolism	Preop	**0.83 (0.82–0.84**)	0.82 (0.81–0.83)	**0.83 (0.82–0.84**)	**0.83 (0.82–0.84**)
	Periop	0.83 (0.82–0.84)	**0.84 (0.83–0.85**)	**0.84 (0.83–0.85**)	**0.84 (0.83–0.85**)
Prolonged mechanical ventilation	Preop	0.90 (0.89–0.91)	0.90 (0.89–0.91)	0.90 (0.90–0.91)	0.90 (0.90–0.91)
	Periop	0.91 (0.91–0.92)	**0.92 (0.92–0.93**)	**0.92 (0.91–0.93**)	**0.92 (0.91–0.92**)
Neurological complications, including delirium	Preop	0.85 (0.85–0.86)	0.85 (0.85–0.86)	0.85 (0.85–0.86)	0.85 (0.85–0.86)
	Periop	0.85 (0.85–0.86)	**0.86 (0.85–0.86**)	**0.86 (0.85–0.86**)	**0.86 (0.85–0.86**)*
Wound complications	Preop	0.79 (0.78–0.80)	0.79 (0.78–0.80)	**0.80 (0.79–0.80**)	**0.80 (0.79–0.80**)
	Periop	0.80 (0.79–0.80)	**0.81 (0.80–0.81**)	**0.81 (0.80–0.81**)*	**0.81 (0.80–0.81**)
Acute kidney injury	Preop	0.82 (0.81–0.82)	0.82 (0.81–0.82)	0.82 (0.82–0.83)	0.82 (0.82–0.83)
	Periop	0.82 (0.82–0.83)	**0.83 (0.82–0.84**)	**0.83 (0.82–0.84**)	**0.83 (0.83–0.84**)*
In-hospital mortality	Preop	**0.90 (0.88–0.91**)	0.89 (0.87–0.90)	**0.90 (0.89–0.91**)	0.89 (0.87–0.90)
	Periop	0.90 (0.89–0.91)	**0.91 (0.90–0.92**)	**0.91 (0.90–0.92**)	**0.91 (0.89–0.92**)

Bold values indicate the best performance.

* The *P* values represent a difference <0.05 compared with the central learning model for each site.

CL indicates central learning; Periop, perioperative; Preop, preoperative.

**TABLE 3.** Comparison of AUROC With 95% Confidence Interval for Central Learning and Federated Learning Models in the UFH JAX Cohort

Outcome	Period	CL	FedAvg	FedProx	SCAFFOLD
Prolonged ICU stay	Preop	**0.89 (0.89–0.90**)	0.87 (0.87–0.88)*	0.87 (0.86–0.87)*	0.87 (0.86–0.87)*
	Periop	**0.91 (0.90–0.91**)	0.89 (0.88–0.89)*	0.89 (0.88–0.90)*	0.90 (0.89–0.90)*
Sepsis	Preop	**0.89 (0.88–0.90**)	0.88 (0.87–0.89)	0.88 (0.87–0.89)	0.88 (0.87–0.89)
	Periop	**0.90 (0.89–0.91**)	0.89 (0.88–0.90)	0.89 (0.88–0.90)	0.89 (0.88–0.90)
Cardiovascular complication	Preop	**0.80 (0.78–0.82**)	0.79 (0.77–0.80)	0.78 (0.77–0.80)	0.79 (0.78–0.80)
	Periop	0.84 (0.83–0.85)	0.84 (0.83–0.85)	0.84 (0.82–0.85)	0.84 (0.83–0.85)
Venous thromboembolism	Preop	**0.81 (0.79–0.83**)	0.80 (0.78–0.82)	0.79 (0.77–0.82)	0.79 (0.77–0.81)
	Periop	**0.83 (0.81–0.85**)	0.82 (0.80–0.84)	0.82 (0.80–0.84)	**0.83 (0.81–0.85**)
Prolonged mechanical ventilation	Preop	**0.84 (0.82–0.86**)	0.83 (0.82–0.85)	0.82 (0.81–0.84)	**0.84 (0.82–0.85**)
	Periop	**0.86 (0.85–0.88**)	0.85 (0.84–0.87)	**0.86 (0.84–0.87**)	**0.86 (0.85–0.88**)
Neurological complications, including delirium	Preop	**0.84 (0.83–0.85**)	**0.84 (0.83–0.85**)	0.83 (0.82–0.84)	**0.84 (0.83–0.85**)
	Periop	**0.85 (0.84–0.86**)	0.84 (0.83–0.85)	0.84 (0.83–0.85)	0.84 (0.83–0.85)
Wound complications	Preop	**0.72 (0.70–0.73**)	0.71 (0.70–0.72)	0.71 (0.70–0.73)	0.71 (0.70–0.72)
	Periop	**0.74 (0.72–0.75**)	0.73 (0.72–0.75)	0.73 (0.72–0.75)	**0.74 (0.72–0.75**)
Acute kidney injury	Preop	0.79 (0.78–0.81)	0.79 (0.78–0.80)	0.79 (0.78–0.80)	0.79 (0.77–0.80)
	Periop	**0.81 (0.80–0.82**)	**0.81 (0.79–0.82**)	0.80 (0.79–0.82)	**0.81 (0.80–0.82**)
In-hospital mortality	Preop	**0.92 (0.90–0.94**)	0.90 (0.87–0.92)	0.90 (0.87–0.92)	0.90 (0.88–0.92)
	Periop	**0.93 (0.91–0.95**)	0.92 (0.90–0.94)	**0.93 (0.91–0.95**)	0.92 (0.90–0.94)

Bold values indicate the best performance.

* The *P* values represent a difference <0.05 compared with the central learning model for each site.

CL indicates central learning; Periop, perioperative; Preop, preoperative.

**TABLE 4.** Comparison of AUPRC With 95% Confidence Interval for Central Learning and Federated Learning Models in the UF GNV Cohort

Outcome	Period	CL	FedAvg	FedProx	SCAFFOLD
Prolonged ICU stay	Preop	0.82 (0.81-0.83)	**0.84** **(0.83-0.84**)	**0.84** **(0.83-0.85**)	**0.84** **(0.83-0.84**)
	Periop	0.86 (0.85–0.86)	**0.87 (0.86–0.87**)	**0.87 (0.86–0.87**)	**0.87 (0.87–0.88**)
Sepsis	Preop	0.53 (0.51–0.55)	0.53 (0.51–0.55)	**0.54 (0.52–0.56**)	**0.54 (0.52–0.56**)
	Periop	0.55 (0.53–0.571	**0.56 (0.54–0.58**)	**0.56 (0.54–0.58**)	**0.56 (0.54–0.58**)
Cardiovascular complication	Preop	0.52 (0.51–0.54)	0.52 (0.51–0.54)	**0.53 (0.52–0.54**)	**0.53 (0.51–0.54**)
	Periop	0.59 (0.57–0.60)	0.59 (0.58–0.61)	0.59 (0.58–0.61)	0.59 (0.58–0.60)
Venous thromboembolism	Preop	0.26 (0.24–0.27)	0.26 (0.25–0.28)	0.26 (0.24–0.28)	**0.27 (0.25–0.29**)
	Periop	0.25 (0.23–0.27)	0.28 (0.26–0.30)	0.27 (0.25–0.29)	**0.29 (0.27–0.31**)
Prolonged mechanical ventilation	Preop	0.56 (0.54–0.58)	0.56 (0.54–0.58)	0.56 (0.54–0.58)	0.56 (0.54–0.58)
	Periop	0.60 (0.58–0.62)	**0.62 (0.61–0.64**)	**0.62 (0.60–0.64**)	**0.62 (0.60–0.63**)
Neurological complications, including delirium	Preop	0.67 (0.66–0.68)	0.67 (0.66–0.68)	**0.68 (0.67–0.69**)	**0.68 (0.67–0.69**)
	Periop	0.67 (0.66–0.68)	0.68 (0.67–0.70)	0.68 (0.67–0.69)	**0.69 (0.68–0.70**)
Wound complications	Preop	0.58 (0.57–0.60)	0.58 (0.57–0.59)	0.59 (0.58–0.61)	**0.60 (0.58–0.61**)
	Periop	0.59 (0.58–0.60)	0.60 (0.59–0.61)	**0.61 (0.59–0.62**)	**0.61 (0.60–0.62**)
Acute kidney injury	Preop	0.52 (0.51–0.53)	**0.53 (0.51–0.54**)	**0.53 (0.51–0.54**)	**0.53 (0.51–0.54**)
	Periop	0.53 (0.52–0.54)	0.55 (0.53–0.56)	0.54 (0.53–0.56)	**0.56 (0.54–0.57**)
In-hospital mortality	Preop	**0.18 (0.15–0.21**)	0.17 (0.15–0.21)	**0.18 (0.15–0.21**)	0.17 (0.14–0.19)
	Periop	0.19 (0.16–0.22)	**0.22 (0.18–0.25**)	0.20 (0.17–0.23)	0.20 (0.17–0.24)

Bold values indicate the best performance.

CL indicates central learning; Periop, perioperative; Preop, preoperative.

**TABLE 5.** Comparison of AUPRC With 95% Confidence Interval for Central Learning and Federated Learning Models in the UF JAX Cohort

Outcome	Period	CL	FedAvg	FedProx	SCAFFOLD
Prolonged ICU stay	Preop	**0.77 (0.75–0.78**)	0.72 (0.70–0.73)	0.71 (0.69–0.72)	0.71 (0.70–0.73)
	Periop	**0.80 (0.78–0.81**)	0.74 (0.72–0.75)	0.73 (0.71–0.75)	0.76 (0.74–0.78)
Sepsis	Preop	**0.52 (0.48–0.55**)	0.49 (0.45–0.52)	0.49 (0.45–0.52)	0.50 (0.46–0.53)
	Periop	**0.53 (0.49–0.56**)	0.52 (0.48–0.55)	0.51 (0.48–0.55)	0.51 (0.48–0.55)
Cardiovascular complication	Preop	**0.36 (0.33–0.39**)	0.32 (0.30–0.35)	0.33 (0.30–0.36)	0.34 (0.32–0.37)
	Periop	**0.43 (0.40–0.46**)	**0.43 (0.40–0.46**)	0.42 (0.39–0.45)	**0.43 (0.40–0.46**)
Venous thromboembolism	Preop	**0.14 (0.12–0.18**)	0.12 (0.10–0.15)	0.13 (0.10–0.16)	**0.14 (0.11–0.18**)
	Periop	**0.16 (0.13–0.20**)	0.15 (0.12–0.18)	0.14 (0.11–0.17)	0.14 (0.12–0.18)
Prolonged mechanical ventilation	Preop	**0.43 (0.39–0.47**)	0.39 (0.35–0.43)	0.38 (0.34–0.42)	0.40 (0.36–0.44)
	Periop	**0.50 (0.46–0.53**)	0.46 (0.43–0.50)	0.45 (0.41–0.49)	0.49 (0.45–0.53)
Neurological complications, including delirium	Preop	**0.48 (0.45–0.51**)	0.47 (0.44–0.50)	0.47 (0.44–0.50)	**0.48 (0.46–0.51**)
	Periop	**0.49 (0.46–0.52**)	0.48 (0.46–0.51)	0.48 (0.45–0.51)	**0.49 (0.46–0.52**)
Wound complications	Preop	**0.37 (0.35–0.40**)	0.36 (0.34–0.39)	0.35 (0.33–0.38)	**0.37 (0.34–0.39**)
	Periop	0.38 (0.36–0.41)	0.37 (0.35–0.39)	0.37 (0.35–0.40)	**0.39 (0.37–0.42**)
Acute kidney injury	Preop	0.40 (0.37–0.43)	**0.41 (0.38–0.44**)	0.40 (0.38–0.43)	**0.41 (0.38–0.43**)
	Periop	0.42 (0.40–0.45)	0.43 (0.41–0.46)	0.43 (0.40–0.45)	**0.44 (0.41–0.46**)
In-hospital mortality	Preop	**0.16 (0.12–0.22**)	0.14 (0.10–0.20)	0.14 (0.10–0.19)	0.14 (0.10–0.19)
	Periop	**0.24 (0.17–0.32**)	0.22 (0.16–0.30)	0.21 (0.16–0.29)	0.22 (0.17–0.30)

Bold values indicate the best performance.

CL indicates central learning; Periop, perioperative; Preop, preoperative.

### Generalizability of Federated Learning Models

**FIGURE 2.**
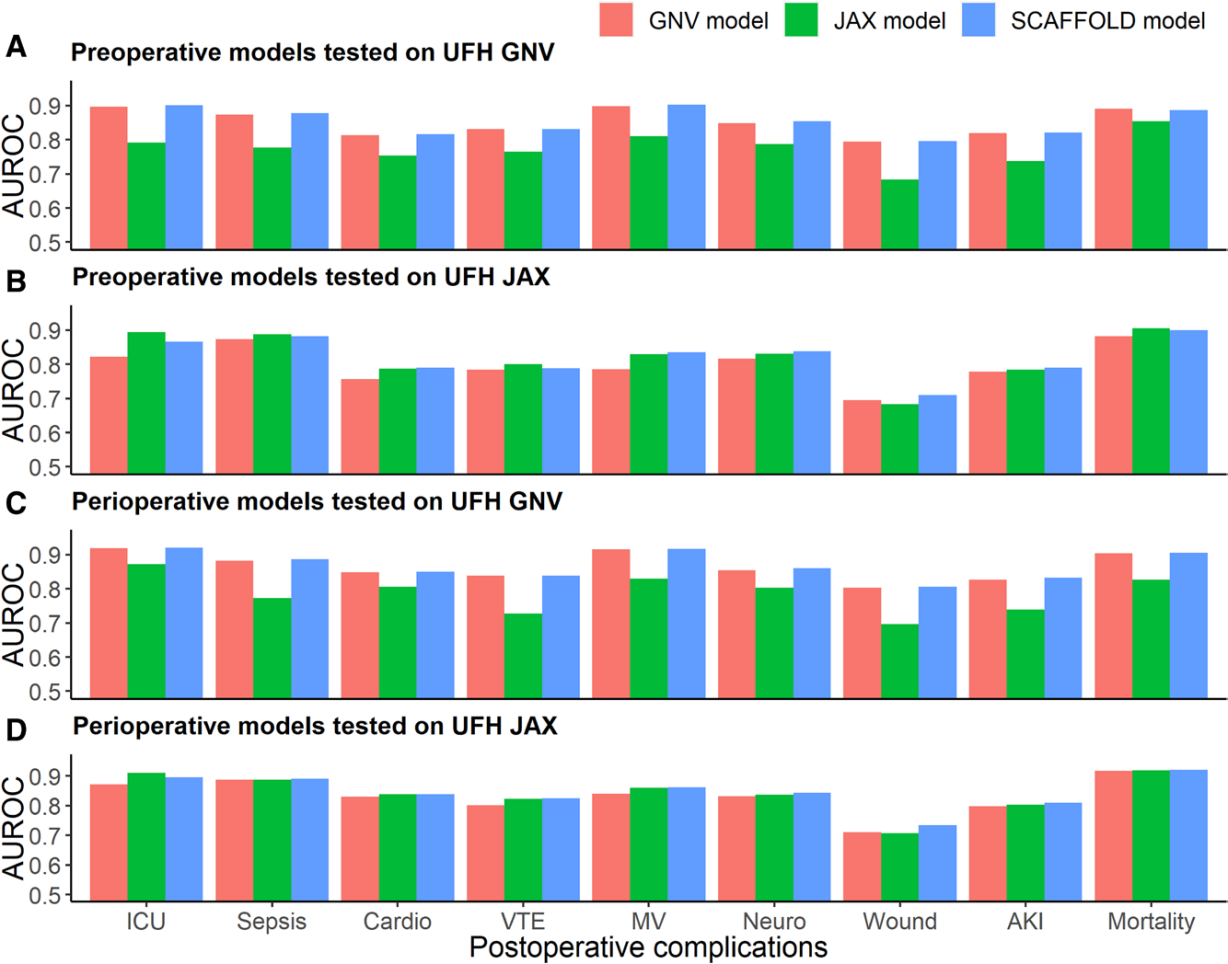
Performance of local learning models and federated learning models. Preoperative models were evaluated on UFH GNV test data (A) and UFH JAX test data (B). Perioperative models were evaluated on UFH GNV test data (C) and UFH JAX test data (D). The full names of complications displayed on the *x*-axis from left to right are: prolonged intensive care unit stay, sepsis, cardiovascular complications, venous thromboembolism, prolonged mechanical ventilation, neurological complications including delirium, wound complications, acute kidney injury, and in-hospital mortality.

We assessed the generalizability of our federated learning model by comparing its performance with 2 local learning models: the GNV model and the JAX model (Figure 2, Tables 6 and 7, and Supplemental Table 3, see https://links.lww.com/AOSO/A493). For simplicity, we only reported the performance of the SCAFFOLD model. Each local learning model demonstrated strong performance within its respective center, but this performance declined when applied to a different center, indicating limited generalizability. That is, at UFH GNV center, GNV model had better AUROC performance than JAX model on all complications; while at UFH JAX center, compared with GNV model, JAX model achieved better AUROC performance on all complications except for wound complications (preoperative models: 0.68 [95% CI, 0.67–0.70] vs 0.70 [95% CI, 0.68–0.71]; perioperative models: 0.71 [95% CI, 0.70–0.72] vs 0.71 [95% CI, 0.70–0.72]).

**TABLE 6.** Comparison of AUROC With 95% Confidence Interval for Local Learning and Federated Learning Preoperative Models

Outcome	Model	GNV Test Data	JAX Test Data
Prolonged ICU stay	GNV model	**0.90 (0.89–0.90**)	0.82 (0.81–0.83)*
	JAX model	0.79 (0.79–0.80)*	**0.89 (0.89–0.90**)*
	SCAFFOLD	**0.90 (0.90–0.91**)	0.87 (0.86–0.87)
	CL	0.89 (0.89–0.90)*	**0.89 (0.89–0.90**)*
Sepsis	GNV model	0.87 (0.87–0.88)	0.87 (0.86–0.89)
	JAX model	0.78 (0.77–0.79)*	**0.89 (0.88–0.90**)
	SCAFFOLD	**0.88 (0.87–0.88**)	0.88 (0.87–0.89)
	CL	**0.88 (0.87–0.88**)	**0.89 (0.88–0.90**)
Cardiovascular complication	GNV model	0.81 (0.81–0.82)	0.76 (0.74–0.77)*
	JAX model	0.75 (0.75–0.76)*	0.79 (0.77–0.80)
	SCAFFOLD	**0.82 (0.81–0.82**)	0.79 (0.78–0.80)
	CL	**0.82 (0.81–0.82**)	**0.80 (0.78–0.82**)
Venous thromboembolism	GNV model	**0.83 (0.82–0.84**)	0.79 (0.76–0.81)
	JAX model	0.77 (0.76–0.78)*	0.80 (0.78–0.82)
	SCAFFOLD	**0.83 (0.82–0.84**)	0.79 (0.77–0.81)
	CL	**0.83 (0.82–0.84**)	**0.81 (0.79–0.83**)
Prolonged mechanical ventilation	GNV model	**0.90 (0.89–0.91**)	0.79 (0.77–0.80)*
	JAX model	0.81 (0.80–0.82)*	0.83 (0.81–0.85)
	SCAFFOLD	**0.90 (0.90–0.91**)	**0.84 (0.82–0.85**)
	CL	**0.90 (0.89–0.91**)	**0.84 (0.82–0.86**)
Neurological complications, including delirium	GNV model	**0.85 (0.84–0.85**)	0.82 (0.81–0.83)*
	JAX model	0.79 (0.78–0.79)*	0.83 (0.82–0.84)
	SCAFFOLD	**0.85 (0.85–0.86**)	**0.84 (0.83–0.85**)
	CL	**0.85 (0.85–0.86**)	**0.84 (0.83–0.85**)
Wound complications	GNV model	**0.80 (0.79–0.80**)	0.70 (0.68–0.71)
	JAX model	0.68 (0.68–0.69)*	0.68 (0.67–0.70)*
	SCAFFOLD	**0.80 (0.79–0.80**)	0.71 (0.70–0.72)
	CL	0.79 (0.78–0.80)	**0.72 (0.70–0.73**)
Acute kidney injury	GNV model	**0.82 (0.81–0.83**)	0.78 (0.77–0.79)
	JAX model	0.74 (0.73–0.75)*	0.78 (0.77–0.80)
	SCAFFOLD	**0.82 (0.82–0.83**)	**0.79 (0.78–0.80**)
	CL	**0.82 (0.81–0.82**)	**0.79 (0.78–0.81**)
In-hospital mortality	GNV model	0.89 (0.88–0.90)	0.88 (0.86–0.91)
	JAX model	0.85 (0.84–0.87)*	0.91 (0.88–0.93)
	SCAFFOLD	0.89 (0.87–0.90)	0.90 (0.88–0.92)
	CL	**0.90 (0.88–0.91**)	**0.92 (0.90–0.94**)

Bold values indicate the best performance.

* The *P* values represent a difference <0.05 compared with the SCAFFOLD model for each site.

CL indicates central learning.

**TABLE 7.** Comparison of AUROC With 95% Confidence Interval for Local Learning and Federated Learning Perioperative Models

Outcome	Model	GNV Test Data	JAX Test Data
Prolonged ICU stay	GNV model	**0.92 (0.92–0.92**)	0.87 (0.87–0.88)*
	JAX model	0.87 (0.87–0.88)*	**0.91 (0.91–0.92**)*
	SCAFFOLD	**0.92 (0.92–0.92**)	0.90 (0.89–0.90)
	CL	0.91 (0.91–0.92)*	**0.91 (0.90–0.91**)*
Sepsis	GNV model	0.88 (0.88–0.89)	0.89 (0.88–0.90)
	JAX model	0.77 (0.76–0.78)*	0.89 (0.88–0.90)
	SCAFFOLD	**0.89 (0.88–0.89**)	0.89 (0.88–0.90)
	CL	**0.89 (0.88–0.89**)	**0.90 (0.89–0.91**)
Cardiovascular complication	GNV model	**0.85 (0.84–0.85**)	0.83 (0.82–0.84)
	JAX model	0.81 (0.80–0.81)*	**0.84 (0.83–0.85**)
	SCAFFOLD	**0.85 (0.84–0.86**)	**0.84 (0.83–0.85**)
	CL	**0.85 (0.84–0.85**)	**0.84 (0.83–0.85**)
Venous thromboembolism	GNV model	**0.84 (0.83–0.85**)	0.80 (0.78–0.82)
	JAX model	0.73 (0.71–0.74)*	0.82 (0.80–0.84)
	SCAFFOLD	**0.84 (0.83–0.85**)	**0.83 (0.81–0.85**)
	CL	0.83 (0.82–0.84)	**0.83 (0.81–0.85**)
Prolonged mechanical ventilation	GNV model	**0.92 (0.91–0.92**)	0.84 (0.83–0.86)
	JAX model	0.83 (0.82–0.84)*	**0.86 (0.84–0.88**)
	SCAFFOLD	**0.92 (0.91–0.92**)	**0.86 (0.85–0.88**)
	CL	0.91 (0.91–0.92)	**0.86 (0.85–0.88**)
Neurological complications, including delirium	GNV model	0.85 (0.85–0.86)	0.83 (0.82–0.84)
	JAX model	0.80 (0.80–0.81)*	0.84 (0.83–0.85)
	SCAFFOLD	**0.86 (0.86–0.86**)	0.84 (0.83–0.85)
	CL	0.85 (0.85–0.86)*	**0.85 (0.84–0.86**)
Wound complications	GNV model	0.80 (0.80–0.81)	0.71 (0.70–0.72)*
	JAX model	0.70 (0.69–0.70)*	0.71 (0.70–0.72)*
	SCAFFOLD	**0.81 (0.80–0.81**)	**0.74 (0.72–0.75**)
	CL	0.80 (0.79–0.80)	**0.74 (0.72–0.75**)
Acute kidney injury	GNV model	**0.83 (0.82–0.83**)	0.80 (0.79–0.81)
	JAX model	0.74 (0.73–0.75)*	0.80 (0.79–0.82)
	SCAFFOLD	**0.83 (0.83–0.84**)	**0.81 (0.80–0.82**)
	CL	0.82 (0.82–0.83)*	**0.81 (0.80–0.82**)
In-hospital mortality	GNV model	0.90 (0.89–0.91)	0.92 (0.90–0.94)
	JAX model	0.83 (0.80–0.85)*	0.92 (0.90–0.94)
	SCAFFOLD	**0.91 (0.89–0.92**)	0.92 (0.90–0.94)
	CL	0.90 (0.89–0.91)	**0.93 (0.91–0.95**)

Bold values indicate the best performance.

* The *P* values represent a difference <0.05 compared with the SCAFFOLD model for each site.

CL indicates central learning.

On the other hand, our federated learning model obtained comparable performance to the best local learning model at each center, demonstrating strong generalizability. That is, at UFH GNV center, the federated learning model achieved performance comparable to or slightly better than the best local learning model, GNV model, across all complications. Similar comparative results were observed for all complications at UFH JAX center, except for the prolonged ICU stay (preoperative models: 0.87 [95% CI, 0.86-0.87] vs 0.89 [95% CI, 0.89-0.90]; perioperative models: 0.90 [95% CI, 0.89-0.90] vs 0.91 [95% CI, 0.91-0.92]).

The local learning preoperative model trained on a larger cohort (GNV model) performed significantly worse at UFH JAX center compared with the federated learning models across several complications: prolonged ICU stay (0.82 [95% CI, 0.81–0.83] vs 0.87 [95% CI, 0.86–0.87]), cardiovascular complication (0.76 [95% CI, 0.74–0.77] vs 0.79 [95% CI, 0.78–0.82]), prolonged MV (0.79 [95% CI, 0.77–0.80] vs 0.84 [95% CI, 0.82–0.85]), and neurological complications including delirium (0.82 [95% CI, 0.81–0.83] vs 0.84 [95% CI, 0.83–0.85]). These results suggest that the JAX cohort included patients with unique characteristics, making the federated learning model more predictive for these complications compared with the GNV model. We investigated the complications with the largest performance drops: prolonged ICU stay and prolonged MV. Our analysis identified 267 surgery cases where the GNV model produced false positives for prolonged ICU stay complication, which were corrected by the federated learning model. Additionally, we found 191 cases with false negatives for ICU complications, 154 cases with false negatives for prolonged MV, and 68 cases with false positives for prolonged MV. A key observation was the frequent absence of the primary procedure code for these patients, a variable that was crucial for predicting these postoperative complications. The missing rates for these 4 subsets of patients were 45%, 53%, 48%, and 38%, respectively, which were significantly higher than the missing rates in UFH GNV training cohort (5%) and the UFH JAX cohort (36%) (Supplemental Table 1, see https://links.lww.com/AOSO/A493). This highlights the excellent performance of the federated learning approach, SCAFFOLD, in addressing the common challenge of nonindependently and non-IID data distribution in federated learning.

### Robustness of Federated Learning Models

To determine if the performance of federated learning models was independent of patient characteristics, we conducted subgroup analysis based on sex, race, age, and surgery type (Supplemental Tables 4–13, see https://links.lww.com/AOSO/A493). All 3 federated learning models and the central learning model exhibited similar performance patterns across different subgroups. The performance of federated learning models showed significant differences between female and male patients in predicting sepsis, cardiovascular complication, and VTE at both centers, with a notable difference in AKI and in-hospital mortality prediction specifically at UFH JAX center. Performance was not affected by race across all complications at both centers, except for a significant difference observed in predicting sepsis at UFH GNV and in-hospital mortality at both centers. Age had a substantial impact on the models’ performance, with this effect being more pronounced at the UFH GNV center. Surgery type also significantly influenced the models’ performance. At both centers, almost all complications exhibited significant differences across surgery types. Specifically, all models had significantly lower performance for cardiothoracic and neurological surgeries compared with other surgery types.

To determine if the performance of the federated learning model would be influenced by the various sample sizes across centers, we conducted a sensitivity analysis of undersampling the samples from UFH GNV centers to equalize the sample sizes between the 2 centers (Supplemental Table 14, see https://links.lww.com/AOSO/A493). We observed a pronounced performance decline at the larger data provider (UFH GNV) and a slight performance increase at the UFH JAX center (prolonged ICU stay: 0.87 vs 0.89 ± 0.003).

## DISCUSSION

We have developed preoperative and perioperative federated learning models to predict risks of major postoperative complications using large EHR datasets from 2 centers. The federated learning models proved to be more robust than local models. This improvement is attributed to the use of larger and more diverse datasets. The federated learning models also proved to be noninferior to the central learning models while also maintaining data privacy and security.

Our validation results confirmed the robustness of the federated learning models, demonstrating their capability to provide strong predictive performance on nonindependent and non-IID data distributions while preserving patient privacy. The UFH GNV and UFH JAX datasets exhibited notable differences, particularly in the missingness of the primary procedure codes. Procedure codes provide insights into the complexity and invasiveness of a surgical procedure, with more complex procedures typically requiring longer monitoring and extended use of ICU resources. The high rate of missing procedure codes at UFH JAX resulted in the largest performance drop for the local learning model (GNV model) for predicting prolonged ICU stay and prolonged MV complications. However, this degradation was mitigated in the federated learning model, highlighting its robustness in multicenter collaborative studies. These findings align with other studies demonstrating the utility of federated learning in healthcare, especially during the COVID-19 pandemic. For instance, Dayan et al^32^ proposed a federated learning model to predict the future oxygen requirements for patients with COVID-19 using vital signs, laboratory data, and chest X-rays from 20 institutions. The proposed model achieved an increase in generalizability of 38% in terms of AUROC when compared with local training models. Vaid et al^9^ presented federated learning models to predict mortality in hospitalized patients with COVID-19 within 7 days, outperforming local models at most hospitals. Similarly, Feki et al^33^ applied federated learning for COVID-19 screening using chest X-rays, demonstrating that the federated learning model remained robust despite the non-IID and unbalanced properties of the decentralized data, showing comparable performance to a central learning model. Federated learning has also been applied to enhance surgical outcomes. Feng et al^34^ developed a robust federated learning model for identifying high-risk patients with postoperative gastric cancer recurrence using computed tomography images.

Several studies have shown that centers with small sample sizes may benefit from federated learning by granting access to larger and more diverse datasets.^32,34^ However, this trend was not mirrored at the UFH JAX center, despite its smaller sample count. A possible explanation is that the data size of UFH JAX cohort was sufficient to learn their own inherent data representation. In federated learning, updates from various clients are often weighted according to their sample sizes. We increased the weight of UFH JAX cohort in our sensitivity analysis and observed limited improvements at UFH JAX center but obvious performance degradation for large data providers. We conjectured that balancing the data sizes across multiple centers to mitigate bias towards centers with smaller datasets is not advisable, and instead, fine-tuning a trained, federated learning model at each local center may be a better option.

Integrating our predictive models of postoperative complications into EHR systems, both preoperative and perioperative, can offer significant advantages throughout the surgical process by facilitating decision-making and improving patient care at various stages. The preoperative model using only perioperative data, enables clinicians to assess surgical risk early, allowing for more informed decisions regarding surgery candidacy, surgical planning, and preoperative optimization. By identifying high-risk patients before surgery, clinicians can implement personalized care strategies, such as optimizing medical conditions, tailoring anesthesia plans, and determining the need for additional monitoring resources such as invasive hemodynamic monitoring, ICU beds, or specialized care teams.^35^ For example, patients at high risk of postoperative AKI or cardiovascular events could benefit from preoperative interventions such as optimizing fluid status,^19^ controlling blood pressure,^19,36^ managing anemia,^37^ adjusting medication regimens,^19,35^ and implementing prehabilitation.^38,39^ The perioperative model incorporating both preoperative and intraoperative data can continuously update risk scores using real-time data during the surgery such as vital signs and intraoperative hemodynamic fluctuations. This real-time capability allows for dynamic adjustments during the procedure. For example, if the model detects an increasing risk of postoperative complications, such as sepsis or prolonged MV, timely interventions—such as adjusting fluid therapy,^40^ modifying ventilation strategies,^41^ or enhancing intraoperative monitoring—can be applied to mitigate these risks and improve patient outcomes. Additionally, these models can also help guide decisions regarding postoperative care such as the need for invasive monitoring or early ICU admission.

Integrating the predictive models into existing clinical decision-making systems is a challenging task, and the collaborative nature of multicenter federated learning adds further complexity to the process. First, each center should map its database into a common data model, such as the OMOP (Observational Medical Outcomes Partnership) Common Data Model, to ensure consistency and improve interoperability across centers. Second, it is essential to identify a set of common variables for federated learning across all centers. Focusing on routinely collected variables can encourage broader participation from centers and enhance the generalizability of the models. Third, an OMOP-based ETL (Extract, Transform, Load) pipeline should be shared and deployed at each center to automate data preparation into the standardized format required for federated learning. Finally, the federated learning process can be initiated, with each center using the same setup. Secure network communications should also be established to protect data while sharing model updates, ensuring privacy and compliance. Once the model is developed, it can be adapted into each hospital’s system to provide real-time predictions. This process typically includes integrating data from the data center, converting it to the OMOP format, applying the ETL pipeline, running predictive models, providing outputs to physicians, and collecting their feedback.^3,28^

While the federated learning model provides broader insights across diverse populations, local learning remains valuable in certain cases. For instance, local learning can quickly adapt to changing data when the federated model’s performance drops due to data drift, or when resources for federated participation are limited. Additionally, centers with large datasets or unique patient populations may prefer local models. Combining both approaches can be beneficial—federated learning offers a broad perspective, while local models can be fine-tuned to meet specific needs. This combination helps institutions benefit from the wide-ranging insights of federated learning while ensuring predictions remain accurate and relevant to their particular patient population.

The study had several limitations. First, it used data from only 2 hospitals and lacked external validation, resulting in limited population diversity and reducing the generalizability of our findings. Our subgroup analysis across age and surgery type further confirmed this limitation, underscoring the need for more diverse datasets to enhance the robustness and applicability of our models. Second, the study was conducted as a simulation of federated learning, rather than an actual implementation. Certain practical challenges and dynamics of real-world deployment were not tested. For example, EHR data cleaning, labeling, and standardization approaches vary across centers. Other technical issues—like network latency, data synchronization, and varying computation capacities of nodes—may significantly limit the deployment of the federated learning model. Future work should focus on implementing federated learning models in real-world settings, involving more centers, incorporating routinely collected variables, and using standardized data models and pipelines. Additionally, future research can explore predicting other postoperative complications, such as surgical site infections, postoperative bleeding, cardiac dysfunction, and pneumonia, to further enhance the clinical utility of these models in improving outcomes for surgical patients.

## CONCLUSIONS

We developed federated learning models for predicting major postoperative complications after surgery using EHR data from 2 centers. Federated learning models achieved comparable performance to central models and outperformed local models while maintaining data privacy. Further implementation studies are needed to test the federated learning platform across healthcare systems and test the clinical efficacy of these models in improving patient care.

## ACKNOWLEDGMENTS

We gratefully acknowledge the technical support of NVIDIA AI Technology Center (NVAITC) at UF for this research. We would like to acknowledge the Intelligent Clinical Care Center research group for the support provided for this study. We acknowledge the University of Florida Integrated Data Repository (IDR) and the UF Health Office of the Chief Data Officer for providing the analytic data set for this project.

B.S., T.O.-B., P.R., and A.B. contributed to the study design. Y.R., Y.P., A.P., and T.O.-B. drafted the manuscript. Y.P. and Y.R. worked on data processing and analysis. All authors contributed to data interpretation and provided critical revisions.

## Supplementary Material

## References

[1] Lee PHU Gawande AA. The number of surgical procedures in an American lifetime in 3 states. J Am Coll Surg. 2008;207:S75.

[2] Gawande AA Regenbogen SE. Critical need for objective assessment of postsurgical patients. Anesthesiology. 2011;114:1269–1270. 21478732 10.1097/ALN.0b013e318219d76b

[3] Bihorac A Ozrazgat-Baslanti T Ebadi A. MySurgeryRisk: development and validation of a machine-learning risk algorithm for major complications and death after surgery. Ann Surg. 2019;269:652–662. 29489489 10.1097/SLA.0000000000002706 PMC6110979

[4] Weinberg L Ratnasekara V Tran AT. The association of postoperative complications and hospital costs following distal pancreatectomy. Front Surg. 2022;9:890518. 35711711 10.3389/fsurg.2022.890518 PMC9195500

[5] Healy MA Mullard AJ Campbell DA Jr. Hospital and payer costs associated with surgical complications. JAMA Surg. 2016;151:823–830. 27168356 10.1001/jamasurg.2016.0773

[6] Juhn Y Liu H. Artificial intelligence approaches using natural language processing to advance EHR-based clinical research. J Allergy Clin Immunol. 2020;145:463–469. 31883846 10.1016/j.jaci.2019.12.897 PMC7771189

[7] Shickel B Loftus TJ Ruppert M. Dynamic predictions of postoperative complications from explainable, uncertainty-aware, and multi-task deep neural networks. Sci Rep. 2023;13:1224. 36681755 10.1038/s41598-023-27418-5 PMC9867692

[8] Datta S Loftus TJ Ruppert MM. Added value of intraoperative data for predicting postoperative complications: the MySurgeryRisk PostOp Extension. J Surg Res. 2020;254:350–363. 32531520 10.1016/j.jss.2020.05.007 PMC7755426

[9] Vaid A Jaladanki SK Xu J. Federated learning of electronic health records to improve mortality prediction in hospitalized patients with COVID-19: machine learning approach. JMIR Med Inform. 2021;9:e24207. 33400679 10.2196/24207 PMC7842859

[10] Choudhury O Park Y Salonidis T. Predicting adverse drug reactions on distributed health data using federated learning. AMIA Annu Symp Proc. 2019;2019:313–322. 32308824 PMC7153050

[11] Rieke N Hancox J Li W. The future of digital health with federated learning. NPJ Digit Med. 2020;3:119. 33015372 10.1038/s41746-020-00323-1 PMC7490367

[12] Yang Q Liu Y Chen T. Federated machine learning: concept and applications. ACM Trans Intell Syst Technol. 2019;10:1–19.

[13] Brisimi TS Chen R Mela T. Federated learning of predictive models from federated electronic health records. Int J Med Inform. 2018;112:59–67. 29500022 10.1016/j.ijmedinf.2018.01.007 PMC5836813

[14] Ng D Lan X Yao MM. Federated learning: a collaborative effort to achieve better medical imaging models for individual sites that have small labelled datasets. Quant Imaging Med Surg. 2021;11:852–857. 33532283 10.21037/qims-20-595 PMC7779924

[15] Huang CT Wang TJ Kuo LK. Federated machine learning for predicting acute kidney injury in critically ill patients: a multicenter study in Taiwan. Health Inf Sci Syst. 2023;11:48. 37822805 10.1007/s13755-023-00248-5 PMC10562351

[16] Collins GS Reitsma JB Altman DG. Transparent reporting of a multivariable prediction model for individual prognosis or diagnosis (TRIPOD): the TRIPOD Statement. BMC Med. 2015;13:1. 25563062 10.1186/s12916-014-0241-z PMC4284921

[17] Leisman DE Harhay MO Lederer DJ. Development and reporting of prediction models: guidance for authors from editors of respiratory, sleep, and critical care journals. Crit Care Med. 2020;48:623–633. 32141923 10.1097/CCM.0000000000004246 PMC7161722

[18] Adhikari L Ozrazgat-Baslanti T Ruppert M. Improved predictive models for acute kidney injury with IDEA: intraoperative data embedded analytics. PLoS One. 2019;14:e0214904. 30947282 10.1371/journal.pone.0214904 PMC6448850

[19] Kellum JA Lameire N Aspelin P. Kidney Disease: Improving Global Outcomes (KDIGO) Acute Kidney Injury Work Group. KDIGO clinical practice guideline for acute kidney injury. Kidney Int Suppl. 2012;2:1–138.

[20] Ozrazgat-Baslanti T Motaei A Islam R. Development and validation of computable phenotype to identify and characterize kidney health in adult hospitalized patients. arXiv:190303149. 2019.

[21] Thottakkara P Ozrazgat-Baslanti T Hupf BB. Application of machine learning techniques to high-dimensional clinical data to forecast postoperative complications. PLoS One. 2016;11:e0155705. 27232332 10.1371/journal.pone.0155705 PMC4883761

[22] Hobson C Ozrazgat-Baslanti T Kuxhausen A. Cost and mortality associated with postoperative acute kidney injury. Ann Surg. 2015;261:1207–1214. 24887982 10.1097/SLA.0000000000000732 PMC4247993

[23] Bihorac A Ozrazgat-Baslanti T Mahanna E. Long-term outcomes for different forms of stress cardiomyopathy after surgical treatment for subarachnoid hemorrhage. Anesth Analg. 2016;122:1594–1602. 27007075 10.1213/ANE.0000000000001231 PMC4857194

[24] Elixhauser A Steiner C Harris DR. Comorbidity measures for use with administrative data. Med Care. 1998;36:8–27. 9431328 10.1097/00005650-199801000-00004

[25] Wald R Waikar SS Liangos O. Acute renal failure after endovascular vs open repair of abdominal aortic aneurysm. J Vasc Surg. 2006;43:460–466; discussion 466. 16520155 10.1016/j.jvs.2005.11.053

[26] Charlson ME Pompei P Ales KL. A new method of classifying prognostic comorbidity in longitudinal studies: development and validation. J Chronic Dis. 1987;40:373–383. 3558716 10.1016/0021-9681(87)90171-8

[27] VHA. National Drug File Reference Terminology (NDF-RT) Documentation. US Department of Veterans Affairs; 2012.

[28] Ren Y Loftus TJ Datta S. Performance of a machine learning algorithm using electronic health record data to predict postoperative complications and report on a mobile platform. JAMA Netw Open. 2022;5:e2211973. 35576007 10.1001/jamanetworkopen.2022.11973 PMC9112066

[29] McMahan B Moore E Ramage D Hampson S Aguera y Arcas B. Communication-efficient learning of deep networks from decentralized data. In: Proceedings of the 20th International Conference on Artificial Intelligence and Statistics. Proceedings of Machine Learning Research. 2017;54:1273–1282.

[30] Li T Sahu AK Zaheer M Sanjabi M Talwalkar A Smith V. Federated optimization in heterogeneous networks. In: Proceedings of Machine learning and systems. Proceedings of the 3rd MLSys Conference. 2020;2:429–450.

[31] Karimireddy SP Kale S Mohri M Reddi S Stich S Suresh AT. SCAFFOLD: stochastic controlled averaging for federated learning. In: *Proceedings of the 37th International Conference on Machine Learning*. PMLR. 2020;119:5132–5143.

[32] Dayan I Roth HR Zhong A. Federated learning for predicting clinical outcomes in patients with COVID-19. Nat Med. 2021;27:1735–1743. 34526699 10.1038/s41591-021-01506-3 PMC9157510

[33] Feki I Ammar S Kessentini Y. Federated learning for COVID-19 screening from chest X-ray images. Appl Soft Comput. 2021;106:107330. 33776607 10.1016/j.asoc.2021.107330 PMC7979273

[34] Feng B Shi J Huang L. Robustly federated learning model for identifying high-risk patients with postoperative gastric cancer recurrence. Nat Commun. 2024;15:742. 38272913 10.1038/s41467-024-44946-4 PMC10811238

[35] Fleisher LA Fleischmann KE Auerbach AD. 2014 ACC/AHA guideline on perioperative cardiovascular evaluation and management of patients undergoing noncardiac surgery: executive summary: a report of the American College of Cardiology/American Heart Association Task Force on practice guidelines. Developed in collaboration with the American College of Surgeons, American Society of Anesthesiologists, American Society of Echocardiography, American Society of Nuclear Cardiology, Heart Rhythm Society, Society for Cardiovascular Angiography and Interventions, Society of Cardiovascular Anesthesiologists, and Society of Vascular Medicine Endorsed by the Society of Hospital Medicine. J Nucl Cardiol. 2015;22:162–215. 25523415 10.1007/s12350-014-0025-z

[36] Futier E Lefrant JY Guinot PG; INPRESS Study Group. Effect of individualized vs standard blood pressure management strategies on postoperative organ dysfunction among high-risk patients undergoing major surgery: a randomized clinical trial. JAMA. 2017;318:1346–1357. 28973220 10.1001/jama.2017.14172 PMC5710560

[37] Munoz M Gomez-Ramirez S Martin-Montanez E. Perioperative anemia management in colorectal cancer patients: a pragmatic approach. World J Gastroenterol. 2014;20:1972–1985. 24587673 10.3748/wjg.v20.i8.1972 PMC3934467

[38] Gillis C Li C Lee L. Prehabilitation versus rehabilitation: a randomized control trial in patients undergoing colorectal resection for cancer. Anesthesiology. 2014;121:937–947. 25076007 10.1097/ALN.0000000000000393

[39] Shakya P Poudel S. Prehabilitation in patients before major surgery: a review article. JNMA J Nepal Med Assoc. 2022;60:909–915. 36705159 10.31729/jnma.7545 PMC9924929

[40] Levy MM Evans LE Rhodes A. The surviving sepsis campaign bundle: 2018 update. Crit Care Med. 2018;46:997–1000. 29767636 10.1097/CCM.0000000000003119

[41] Futier E Constantin JM Paugam-Burtz C; IMPROVE Study Group. A trial of intraoperative low-tidal-volume ventilation in abdominal surgery. N Engl J Med. 2013;369:428–437. 23902482 10.1056/NEJMoa1301082

