# Supplementary material for: Federated Learning for Predicting Major Postoperative Complications
Source: Ann Surg Open. 2025 May 2;6(2):e573. doi: 10.1097/AS9.0000000000000573 (PMC12185077; doi:10.1097/AS9.0000000000000573)
Supplement: Supplementary file 1 [file as9-6-e573-s001.pdf]

Supplemental Digital Content

Yuanfang Ren, Yonggi Park, Benjamin Shickel, Ziyuan Guan, Ayush Patel, Yingbo Ma, Zhenhong Hu, Jeremy A. Balch, Tyler J. Loftus, Parisa Rashidi, Tezcan Ozrazgat-Baslanti, Azra Bihorac. **Federated learning for predicting major postoperative complications**

This supplementary material has been provided by the authors to give readers additional information about their work.

SDC Figure 1. Cohort selection and exclusion criteria

SDC Figure 2. Distribution of intraoperative features used for model development in training cohort across the sites

SDC Table 1. Characteristics of preoperative features used for model development in training cohort across the sites

SDC Table 2. Patient characteristics

SDC Table 3. Comparison of AUPRC for local learning and federated learning models

SDC Table 4. Subgroup analysis of AUROC with 95% confidence interval for federated learning models based on sex in the UFH GNV cohort

SDC Table 5. Subgroup analysis of AUROC with 95% confidence interval for federated learning models based on sex in the UFH JAX cohort

SDC Table 6. Subgroup analysis of AUROC with 95% confidence interval for federated learning models based on race in the UFH GNV cohort

SDC Table 7. Subgroup analysis of AUROC with 95% confidence interval for federated learning models based on race in the UFH JAX cohort

SDC Table 8. Subgroup analysis of AUROC with 95% confidence interval for federated learning models based on age in the UFH GNV cohort

SDC Table 9. Subgroup analysis of AUROC with 95% confidence interval for federated learning models based on age in the UFH JAX cohort

SDC Table 10. Subgroup analysis of AUROC with 95% confidence interval across models (central learning and SCAFFOLD models) based on surgery type in the UFH GNV cohort

SDC Table 11. Subgroup analysis of AUROC with 95% confidence interval across models (FedAvg and FedProx models) based on surgery type in the UFH GNV cohort

SDC Table 12. Subgroup analysis of AUROC with 95% confidence interval across models (central learning and SCAFFOLD models) based on surgery type in the UFH JAX cohort

SDC Table 13. Subgroup analysis of AUROC with 95% confidence interval across models (FedAvg and FedProx models) based on surgery type in the UFH JAX cohort

SDC Table 14. Comparison of AUROC for federated learning preoperative models with varied and equal training sample sizes

SDC Figure 1. Cohort selection and exclusion criteria

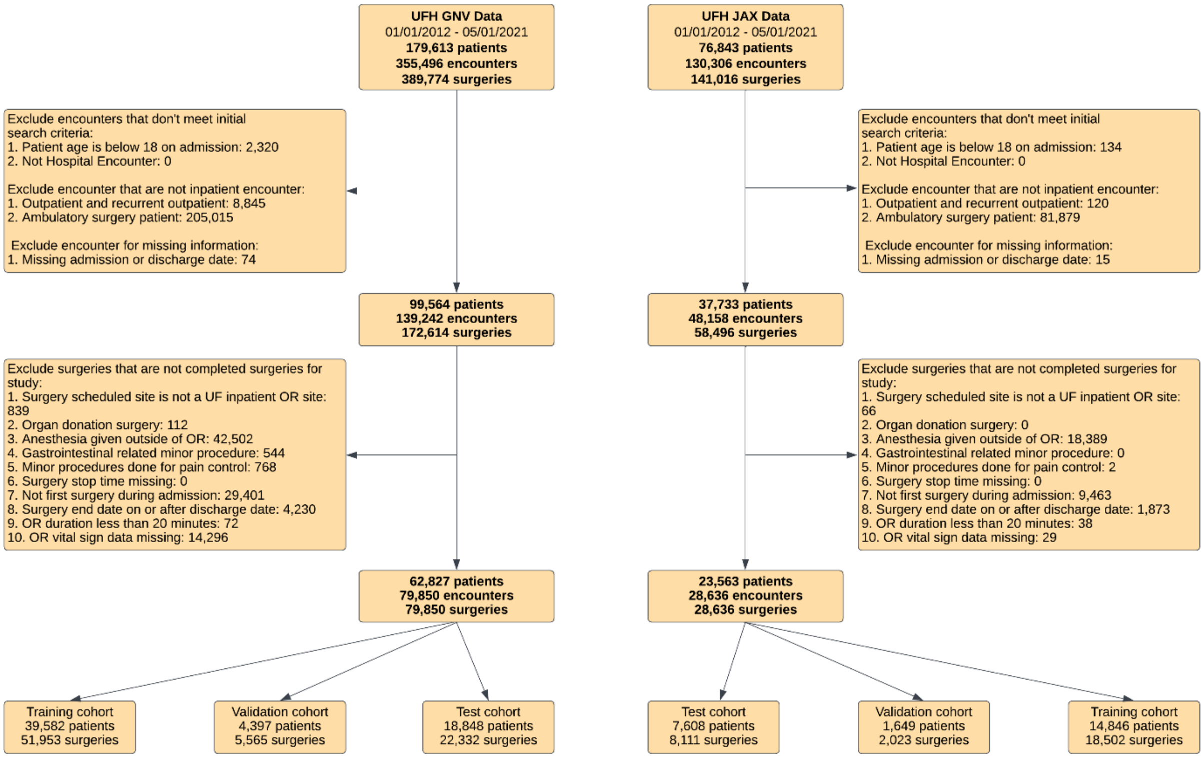

SDC Figure 2. Distribution of intraoperative features used for model development in training cohort across the sites

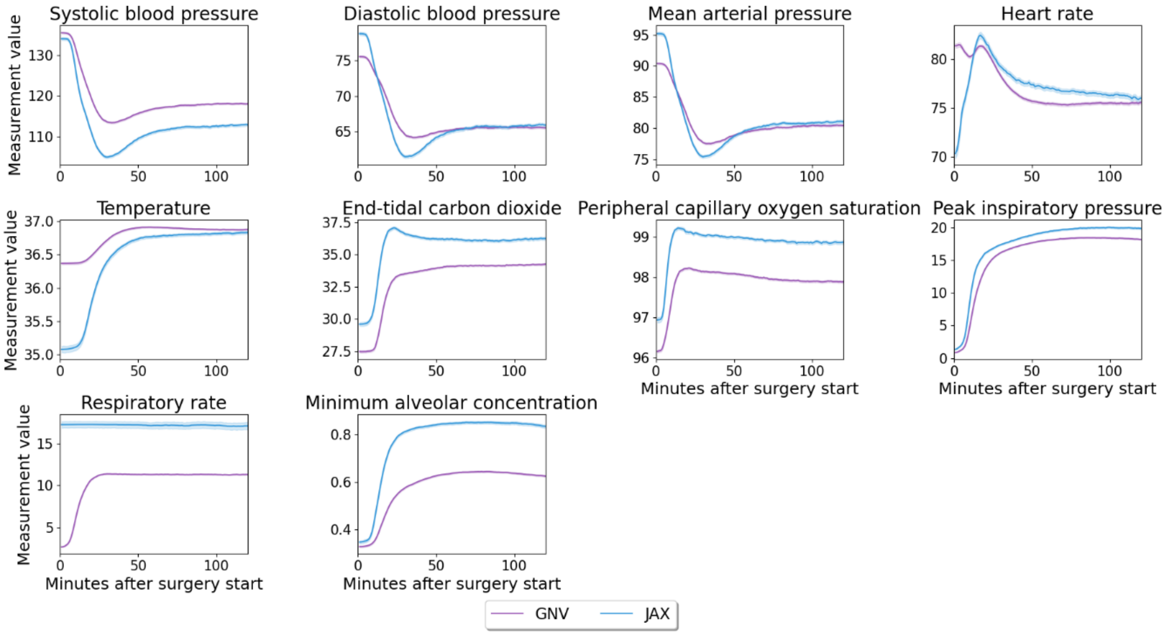

SDC Table 1. Characteristics of preoperative features used for model development in training cohort across the sites

Features	UFH GNV	UFH JAX	P-value
Number of patients, n	39,582	14,846	
Number of encounters, n	51,953	18,502	
**Demographic information**			
Age, years, mean (SD)	57 (17)	52 (17)	<.001
Sex, n (%)			
Male	26,109 (50)	9,620 (52)	<.001
Female	25,844 (50)	8,882 (48)	<.001
Distance of residence to hospital, km, mean (SD)	71 (114)	31 (119)	<.001
Rural at patient residential area, n (%)	17,980 (35)	1,787 (10)	<.001
Total population at patient residential area, mean (SD)	20,506 (13,395)	31,312 (16,300)	<.001
Median total income at patient residential area, USD, mean (SD)	43,254 (12,457)	44,880 (15,656)	<.001
Prevalence of residents living below poverty at patient residential area, %, mean (SD)	20 (10)	21 (11)	0.28
Prevalence of African American residents living below poverty at patient residential area, %, mean (SD)	0.15 (0.15)	0.4 (0.3)	<.001
Prevalence of Hispanic residents living below poverty at patient residential area, %, mean (SD)	0.08 (0.07)	0.1 (0.0)	<.001
County, n (%)			
1st Rank	12,170 (23)	12,890 (70)	
2nd Rank	5,547 (11)	1,593 (9)	
3rd Rank	2,321 (4)	1,179 (6)	
Zip code, n (%)			
1st Rank	1,925 (4)	1,698 (9)	
2nd Rank	1,139 (2)	1,567 (8)	
3rd Rank	1,137 (2)	1,197 (6)	
Marital Status, n (%)			
Married	25,092(48)	6,222 (34)	<.001
Single	15,412(30)	6,675 (36)	<.001
Divorced	8,215 (16)	4,968 (27)	<.001
Missing	3,234 (6)	637 (3)	<.001
Native Language Spoken, n (%)			
English	51,025 (98)	17,962 (97)	<.001
Non-English	928 (2)	540 (3)	<.001
Insurance paying the bills, n (%)			
Medicare	23,483 (45)	5,602 (30)	<.001
Private	15,840 (30)	5,154 (28)	<.001
Medicaid	8,646 (17)	7,493 (41)	<.001
Uninsured	3,984 (8)	253 (1)	<.001
Race, n (%)[a]			
African American	7,166 (14)	6,435 (35)	<.001
White	40,874(79)	10,904 (59)	<.001
Other[b]	3,197 (6)	1,100 (6)	0.32

**Features**	**UFH GNV**	**UFH JAX**	**P-value**
Missing	716 (1)	63 (0.3)	<.001
Ethnicity, n (%)[a]			
Non-Hispanic	48,946 (96)	17,623 (96)	<.001
Hispanic	2,195 (4)	784 (4)	0.96
Smoking Status, n (%)			
Never	21,607 (42)	7,130 (39)	<.001
Former	17,758 (34)	5,102 (30)	<.001
Current	9,511 (18)	5,549 (28)	<.001
Missing	3,077 (6)	721 (4)	<.001
Body Mass Index, median (IQR)	28 (24, 33)	28 (24, 33)	<.001
**Surgical information, n (%)**			
Time from Admission to Surgery, days, median (IQR)	3 (2, 21)	4 (2, 30)	<.001
Emergency admission	20,589 (40)	10214 (55)	<.001
The admission happened at night	24,613 (47)	9104 (49)	<.001
Admission type			
Surgery	36,789 (85)	15,089 (82)	<.001
Medicine	6,307 (15)	3,413 (18)	<.001
Transferred from another hospital	8,077 (16)	1,085 (6)	<.001
Anesthesia Type			
General	46,547 (90)	18,112 (98)	<.001
Local/regional	5,406 (10)	390 (2)	<.001
Scheduled room is trauma room	375 (1)	0 (0)	<.001
Scheduled post operation location is ICU	9,999 (19)	295 (2)	<.001
Scheduled surgery room			
1st Rank	3,433 (7)	1,582 (9)	
2nd Rank	2,726 (5)	1,462 (8)	
3rd Rank	2,716 (5)	1,458 (8)	
Attending Surgeon			
1st Rank	8,857 (17)	1,040 (6)	
2nd Rank	1,421(3)	981 (5)	
3rd Rank	1,143 (2)	736 (4)	
Current Procedural Terminology code of the primary procedure			
1st Rank	1,235 (3)	610 (5)	
2nd Rank	881 (2)	270 (2)	
3rd Rank	767 (2)	246 (2)	
Missing	2,603 (5)	6,713 (36)	<.001
Admission Day			
Monday	10,076 (19)	3,531 (19)	0.36
Tuesday	9,944 (19)	2,932 (16)	<.001
Wednesday	8,722 (17)	3,002 (16)	0.08
Sunday	8,575 (17)	3,359 (18)	<.001

**Features**	**UFH GNV**	**UFH JAX**	**P-value**
Thursday	8,656 (17)	2,913 (16)	.003
Friday	3,210 (6)	1,372 (7)	<.001
Saturday	2,770 (5)	1,393 (8)	<.001
Admission Month			
Aug	4,562 (9)	1,626 (9)	0.99
Jul	4,438 (9)	1,591 (9)	0.82
Jun	4,452 (9)	1,582 (8)	0.95
Oct	4,643 (9)	1,535 (8)	0.008
Sep	4,249 (8)	1,466 (8)	0.28
Mar	4,116 (8)	1,663 (9)	<.001
Jan	4,849 (9)	1,492 (8)	<.001
May	3,852 (7)	1,567 (8)	<.001
Feb	4,353 (8)	1,456 (8)	0.03
Dec	4,373 (8)	1,490 (8)	0.13
Nov	4,153 (8)	1,417 (8)	0.15
Apr	3,913 (8)	1,617 (9)	<.001
Surgery Type			
Orthopedic surgery	10,967 (21)	4,009 (22)	0.11
Neurosurgery	5,947 (11)	1,687 (9)	<.001
Vascular surgery	3,485 (7)	763 (4)	<.001
Urology	4,055 (8)	1,012 (6)	<.001
Ear Nose Throat	3,092 (6)	2,374 (13)	<.001
Cardio Thoracic Surgery	2,885 (6)	1,189 (6)	<.001
Gastrointestinal Surgery	2,726 (5)	0 (0)	<.001
OB Gynecology	1,854 (4)	1,484 (8)	<.001
Surgical Oncology	1,687 (3)	0 (0)	<.001
Other[c]	15,255 (29)	5,984 (32)	<.001
**Medication within one year before admission date, n (%)**			
Number of kinds of nephrotoxic drugs received, median (IQR)	0 (0,1)	0 (0,1)	<.001
ACE Inhibitors	4,996 (10)	2,173 (12)	<.001
Aminoglycosides	1,825 (4)	1,336 (7)	<.001
Antiemetic	14,754 (28)	5,178 (28)	0.29
Aspirin	6,589 (13)	2,314 (12)	0.54
Betablockers	8,342 (16)	2,610 (14)	<.001
Bicarbonate	1,742 (3)	308 (2)	<.001
Diuretics	5,542 (11)	1,930 (10)	0.38
Vasopressors or inotropes	9,482 (18)	3,644 (20)	<.001
Statin	4,482 (9)	1,505 (8)	0.04
Vancomycin	5,676 (11)	1,789 (10)	<.001
Nonsteroidal anti-inflammatory drugs	7,195 (14)	3,279 (18)	<.001

Features	UFH GNV	UFH JAX	P-value
**Comorbidities within one year before admission date, n (%)**			
Charlson comorbidity index, median (IQR)	2 (0, 4)	2 (0, 4)	0.12
Alcohol or drug abuse	6,956 (13)	4,816 (26)	<.001
Myocardial Infarction	2,970 (6)	1,331 (7)	<.001
Congestive Heart Failure	6,739 (13)	2,282 (12)	0.03
Peripheral Vascular Disease	10,006 (19)	4,146 (22)	<.001
Cerebrovascular Disease	6,812 (13)	2,370 (13)	0.30
Chronic Pulmonary Disease	13,931 (27)	5,281 (28)	<.001
Cancer	11,937 (23)	3,672 (20)	<.001
Metastatic Carcinoma	4,190 (8)	1,301 (7)	<.001
Liver Disease	6,093 (12)	2,600 (14)	<.001
Diabetes	11,335 (22)	4,685 (25)	<.001
Hypertension	7,190 (14)	3,102 (17)	<.001
Obesity	14,061 (27)	6,291 (34)	<.001
Fluid and electrolyte disorders	11,557 (22)	5,523 (30)	<.001
Valvular Disease	5,460 (11)	3,465 (19)	<.001
Coagulopathy	3,946 (8)	1,407 (8)	0.98
Weight Loss	6,854 (13)	3,382 (18)	<.001
Depression	12,778 (25)	4,535 (24)	0.83
Chronic anemia	4,255 (8)	2,459 (13)	<.001
Chronic Kidney Disease	8,444 (16)	3,332 (18)	<.001
**Laboratory results information**			
Have pH test with 7 days prior to surgery, n (%)	2,493 (5)	1,187 (6)	<.001
Have pH test with 8-365 days prior to surgery, n (%)	4,246 (8)	1,381 (7)	0.002
Automated urinalysis, urine glucose within 7 days prior to surgery, mg/dL, n (%)			
Missing	39,184 (75)	14,021 (76)	0.33
Negative	11,096 (21)	3,735 (20)	<.001
Small	931 (2)	374 (2)	0.05
Moderate	382 (1)	293 (2)	<.001
Large	360 (1)	79 (0.4)	<.001
Automated urinalysis, urine glucose within 8-365 days prior to surgery, mg/dL, n (%)			
Missing	36,131 (70)	12,098 (65)	<.001
Negative	13,031 (25)	5,197 (28)	<.001
Small	1,467 (3)	535 (3)	0.65
Moderate	648 (1)	409 (2)	<.001
Large	676 (1)	263 (1)	0.23
Automated urinalysis, urine protein presence within 365 days prior to surgery, mg/dL, n (%)			
Missing	27,278 (53)	8,970 (48)	<.001

Features	UFH GNV	UFH JAX	P-value
Negative	10,449 (20)	3,860 (21)	0.03
Moderate	9,271 (18)	3,866 (21)	<.001
Small	3,209 (6)	1,049 (6)	0.01
Large	1,746 (3)	757 (4)	<.001
Automated urinalysis, urine red blood cell within 365 days prior to surgery, /hpf, n (%)			
Missing	36,435 (70)	11,641 (63)	<.001
Negative	9,224 (18)	4,259 (23)	<.001
Small	3,332 (6)	1,439 (8)	<.001
Large	2,453 (5)	969 (5)	0.005
Moderate	509 (1)	194 (1)	0.44
Automated urinalysis, urine hemoglobin within 7 days prior to surgery, mg/dL, n (%)			
Missing	39,891 (77)	14,874 (80)	<.001
Negative	7,143 (14)	2,077 (11)	<.001
Small	2,520 (5)	699 (4)	<.001
Moderate	1,342 (3)	463 (3)	0.57
Large	1,057 (2)	389 (2)	0.60
Automated urinalysis, urine hemoglobin within 8-365 days prior to surgery, mg/dL, n (%)			
Missing	38,935 (75)	13,161 (71)	<.001
Negative	6,690 (13)	2,934 (16)	<.001
Small	2,839 (5)	1,017 (5)	0.88
Large	1,807 (3)	697 (4)	0.07
Moderate	1,682 (3)	693 (4)	0.001
Hemoglobin within 7 days prior to surgery, g/dl, median (IQR)			
Minimum	12.60 (10.8, 13.9)	12.0 (10.3, 13.5)	<.001
Maximum	13.2 (11.7, 14.4)	12.8 (11.3, 14.0)	<.001
Average	12.9 (11.2, 14.1)	12.4 (10.8, 13.7)	<.001
Variance	0 (0, 0.3)	0 (0, 0.5)	<.001
Count	1 (0, 2)	1 (0, 2)	<.001
Hemoglobin within 8-365 days prior to surgery, g/dl, median (IQR)			
Minimum	12.0 (9.7, 13.6)	11.6 (9.5, 13.2)	<.001
Maximum	13.7 (12.5, 14.8)	13.3 (12.1, 14.4)	<.001
Average	12.7 (11.0, 13.9)	12.3 (10.8, 13.6)	<.001
Variance	0.4 (0, 1.3)	0.4 (0, 1.3)	<.001
Count	1 (0, 4)	1 (0, 4)	<.001

Features	UFH GNV	UFH JAX	P-value
Glucose in blood within 7 days prior to surgery, mg/dL, median (IQR)			
Minimum	100 (88, 119)	99 (87, 119)	0.04
Maximum	118 (98, 155)	122 (100, 162)	<.001
Average	110 (95, 135)	111 (96, 137)	<.001
Variance	0 (0, 283)	22 (0, 374)	<.001
Count	1 (0, 2)	1 (0, 3)	<.001
Glucose in blood within 8-365 days prior to surgery, mg/dL, median (IQR)			
Minimum	89 (79, 101)	88 (77, 99)	<.001
Maximum	128 (101, 180)	127 (101, 185)	0.64
Average	108 (95, 128)	106 (94, 127)	<.001
Variance	168 (0, 669)	199 (5, 742)	<.001
Count	1 (0, 5)	1 (0, 6)	<.001
Urea nitrogen in blood within 7 days prior to surgery, mg/dL, median (IQR)			
Minimum	14.0 (10.0, 19.0)	12.0 (9.0, 17.0)	<.001
Maximum	16.0 (12.0, 21.0)	14.0 (11.0, 19.0)	<.001
Average	15.0 (11.0, 20.0)	13.0 (10.0, 18.0)	<.001
Variance	0.0 (0.0, 2.9)	0.0 (0.0, 4.3)	<.001
Count	1 (0, 2)	1 (0, 2)	<.001
Urea nitrogen in blood within 8-365 days prior to surgery, mg/dL, median (IQR)			
Minimum	12.0 (8.0, 16.0)	11.0 (8.0, 15.0)	<.001
Maximum	18.0 (13.0, 24.0)	16.0 (12.0, 22.0)	<.001
Average	14.7 (11.1, 19.0)	13.5 (10.5, 17.8)	<.001
Variance	4.3 (0.0, 15.5)	4.5 (0.0, 15.9)	<.001
Count	1 (0, 4)	1 (0, 4)	<.001
Serum creatinine within 7 days prior to surgery, mg/dL, median (IQR)			
Minimum	0.8 (0.7, 1.0)	0.8 (0.7, 1.0)	<.001
Maximum	0.9 (0.7, 1.1)	0.9 (0.7, 1.1)	0.03

Features	UFH GNV	UFH JAX	P-value
Average	0.9 (0.7, 1.1)	0.9 (0.7, 1.1)	0.009
Variance	0.0 (0.0, 0.0)	0.0 (0.0, 0.0)	<.001
Count	1 (0, 2)	1 (0, 2)	<.001
Serum creatinine within 8-365 days prior to surgery, mg/dL, median (IQR)			
Minimum	0.8 (0.6, 1.0)	0.8 (0.6, 0.9)	<.001
Maximum	1.0 (0.8, 1.2)	0.9 (0.8, 1.2)	<.001
Average	0.9 (0.7, 1.1)	0.8 (0.7, 1.0)	<.001
Variance	0.0 (0.0, 0.0)	0.0 (0.0, 0.0)	<.001
Count	1 (0, 4)	1 (0, 4)	<.001
Serum Calcium within 7 days prior to surgery, mmol/L, median (IQR)			
Minimum	9.1 (8.6, 9.6)	8.9 (8.4, 9.4)	<.001
Maximum	9.4 (9.0, 9.7)	9.2 (8.9, 9.6)	<.001
Average	9.2 (8.8, 9.6)	9.1 (8.7, 9.4)	<.001
Variance	0.0 (0.0, 0.1)	0.0 (0.0, 0.1)	<.001
Count	1 (0, 2)	1 (0, 2)	<.001
Serum Calcium within 8-365 days prior to surgery, mmol/L, median (IQR)			
Minimum	8.9 (8.2, 9.4)	8.9 (8.3, 9.4)	0.85
Maximum	9.6 (9.3, 9.9)	9.6 (9.3, 9.9)	<.001
Average	9.2 (8.8, 9.6)	9.2 (8.9, 9.5)	0.05
Variance	0.1 (0.0, 0.2)	0.1 (0.0, 0.2)	0.17
Count	1 (0, 4)	1 (0, 4)	<.001
Serum Sodium within 7 days prior to surgery, mmol/L, median (IQR)			
Minimum	138 (136, 140)	138 (136, 140)	0.51
Maximum	140 (138, 142)	140 (138, 142)	<.001
Average	139 (137, 141)	139 (137, 141)	0.003
Variance	0 (0, 2)	0 (0, 3)	<.001
Count	1 (0, 2)	1 (0, 2)	<.001

Features	UFH GNV	UFH JAX	P-value
Serum Sodium within 8-365 days prior to surgery, mmol/L, median (IQR)			
Minimum	137 (134, 140)	137 (134, 140)	0.02
Maximum	141 (139, 143)	141 (140, 143)	<.001
Average	139 (137, 140)	139 (137, 141)	<.001
Variance	2 (0, 6)	3 (0, 8)	<.001
Count	1 (0, 4)	1 (0, 4)	<.001
Urea nitrogen-Creatinine ratio within 7 days prior to surgery, median (IQR)			
Minimum	16.1 (12.4, 20.8)	14.1 (10.6, 18.4)	<.001
Maximum	18.0 (14.1, 23.3)	16.2 (12.5, 21.2)	<.001
Average	17.1 (13.4, 22.0)	15.2 (11.6, 19.7)	<.001
Variance	0.0 (0.0, 2.9)	0.0 (0.0, 3.8)	<.001
Count	0 (0, 0)	1 (0, 2)	<.001
Urea nitrogen-Creatinine ratio within 8-365 days prior to surgery, median (IQR)			
Minimum	14.2 (10.7, 18.8)	12.5 (9.2, 16.3)	<.001
Maximum	20.0 (15.4, 26.1)	18.9 (14.6, 24.6)	<.001
Average	17.2 (13.7, 21.7)	15.7 (12.5, 19.8)	<.001
Variance	2.9 (0.0, 14.7)	5.1 (0.0, 17.0)	<.001
Count	0 (0, 0)	1 (0, 4)	<.001
Potassium in serum within 7 days prior to surgery, mmol/L, median (IQR)			
Minimum	3.9 (3.6, 4.2)	3.8 (3.5, 4.1)	<.001
Maximum	4.2 (3.9, 4.5)	4.1 (3.8, 4.4)	<.001
Average	4.0 (3.8, 4.3)	4.0 (3.7, 4.2)	<.001
Variance	0.0 (0.0, 0.1)	0.0 (0.0, 0.1)	0.03
Count	1 (0, 2)	1 (0, 2)	<.001
Potassium in serum within 8-365 days prior to surgery, mmol/L, median (IQR)			
Minimum	3.8 (3.4, 4.1)	3.8 (3.4, 4.1)	0.89

Features	UFH GNV	UFH JAX	P-value
Maximum	4.4 (4.1, 4.8)	4.4 (4.1, 4.8)	<.001
Average	4.1 (3.9, 4.3)	4.1 (3.8, 4.3)	0.06
Variance	0.1 (0.0, 0.2)	0.1 (0.0, 0.2)	<.001
Count	1 (0, 4)	1 (0, 4)	<.001
Chloride in Serum within 7 days prior to surgery, mmol/L, mmol/L, median (IQR)			
Minimum	101 (98, 103)	100 (97, 102)	<.001
Maximum	102 (100, 105)	102 (99, 104)	<.001
Average	102 (99, 104)	101 (98, 103)	<.001
Variance	0 (0, 2)	0 (0, 4)	<.001
Count	1 (0, 2)	1 (0, 2)	<.001
Chloride in Serum within 8-365 days prior to surgery, mmol/L, mmol/L, median (IQR)			
Minimum	100 (97, 102)	99 (96, 102)	<.001
Maximum	104 (101, 107)	104 (101, 106)	<.001
Average	102 (100, 104)	101 (99, 103)	<.001
Variance	2 (0, 8)	4 (0, 10)	<.001
Count	1 (0, 4)	1 (0, 4)	<.001
Serum CO2 within 7 days prior to surgery, mmol/L, median (IQR)			
Minimum	24 (22, 26)	23 (21, 25)	<.001
Maximum	26 (24, 28)	25 (23, 27)	<.001
Average	25 (23, 27)	24 (22, 26)	<.001
Variance	0 (0, 2)	0 (0, 2)	0.41
Count	1 (0, 2)	1 (0, 2)	<.001
Serum CO2 within 8-365 days prior to surgery, mmol/L, median (IQR)			
Minimum	23 (21, 26)	23 (20, 25)	<.001
Maximum	27 (25, 29)	26 (25, 28)	<.001
Average	25 (23, 27)	24 (23, 26)	<.001
Variance	2 (0, 6)	2 (0, 6)	<.001
Count	1 (0, 4)	1 (0, 4)	<.001
Anion gap in blood within 7 days prior to surgery, mmol/L, median (IQR)			
Minimum	12 (9, 14)	13 (11, 15)	<.001
Maximum	13 (11, 16)	15 (13, 17)	<.001

Features	UFH GNV	UFH JAX	P-value
Average	12 (10, 15)	14 (12, 15)	<.001
Variance	0 (0, 2)	0 (0, 2)	0.40
Count	1 (0, 1)	1 (0, 2)	<.001
Anion gap in blood within 8-365 days prior to surgery, mmol/L, median (IQR)			
Minimum	10 (7, 12)	12 (10, 14)	<.001
Maximum	14 (12, 17)	15 (14, 18)	<.001
Average	12 (10, 14)	13 (12, 15)	<.001
Variance	2 (0, 6)	2 (0, 5)	0.10
Count	0 (0, 2)	1 (0, 3)	<.001
Band form neutrophils/100 leukocytes in blood within 7 days prior to surgery, %, median (IQR)			
Minimum	5 (2, 12)	4 (1, 9)	<.001
Maximum	8 (3, 19)	6 (2, 15)	<.001
Average	7 (3, 16)	5 (2, 12)	<.001
Variance	0 (0, 1)	0 (0, 2)	0.40
Count	0 (0, 0)	0 (0, 0)	<.001
Band form neutrophils/100 leukocytes in blood within 8-365 days prior to surgery, %, median (IQR)			
Minimum	3 (1, 7)	2 (1, 4)	<.001
Maximum	8 (3, 19)	7 (2, 18)	<.001
Average	5 (2, 11)	4 (2, 10)	<.001
Variance	0 (0, 25)	0 (0, 29)	0.03
Count	0 (0, 0)	0 (0, 0)	<.001
White Blood Cell in blood within 7 days prior to surgery, thou/uL, median (IQR)			
Minimum	7.6 (6.0, 10.0)	8.1 (6.2, 11.0)	<.001
Maximum	8.7 (6.6, 12.0)	9.6 (7.1, 13.6)	<.001
Average	8.2 (6.4, 10.9)	8.9 (6.7, 12.2)	<.001
Variance	0.0 (0.0, 1.0)	0.0 (0.0, 1.6)	<.001
Count	1 (0, 2)	1 (0, 2)	<.001
White Blood Cell in blood within 8-365 days prior to surgery, thou/uL, median (IQR)			
Minimum	6.3 (4.9, 7.9)	6.3 (4.9, 8.0)	0.93
Maximum	9.2 (7.0, 12.9)	9.2 (6.8, 13.0)	0.19
Average	7.7 (6.2, 9.7)	7.8 (6.1, 9.8)	0.77
Variance	0.9 (0.0, 4.7)	1.0 (0.0, 5.1)	<.001

Features	UFH GNV	UFH JAX	P-value
Count	1 (0, 4)	1 (0, 4)	<.001
Serum Red Blood Cell within 7 days prior to surgery, Million/uL, median (IQR)			
Minimum	4.2 (3.7, 4.7)	4.1 (3.6, 4.6)	<.001
Maximum	4.4 (4.0, 4.8)	4.4 (3.9, 4.8)	<.001
Average	4.3 (3.8, 4.7)	4.2 (3.8, 4.7)	<.001
Variance	0.0 (0.0, 0.03)	0.0 (0.0, 0.05)	<.001
Count	1 (0, 2)	1 (0, 2)	<.001
Serum Red Blood Cell within 8-365 days prior to surgery, Million/uL, median (IQR)			
Minimum	4.1 (3.4, 4.6)	4.0 (3.4, 4.5)	0.003
Maximum	4.6 (4.2, 4.9)	4.6 (4.2, 4.9)	<.001
Average	4.3 (3.8, 4.7)	4.2 (3.8, 4.6)	0.006
Variance	0.0 (0.0, 0.1)	0.0 (0.0, 0.2)	<.001
Count	1 (0, 4)	1 (0, 4)	<.001
Hematocrit in blood within 7 days prior to surgery, %, median (IQR)			
Minimum	38.1 (33.0, 41.9)	36.7 (31.9, 40.5)	<.001
Maximum	39.8 (35.8, 43.3)	38.5 (34.6, 41.9)	<.001
Average	38.9 (34.4, 42.4)	37.5 (33.3, 41.0)	<.001
Variance	0.0 (0.0, 3.0)	0.0 (0.0, 3.6)	<.001
Count	1 (0, 2)	1 (0, 2)	<.001
Hematocrit in blood within 8-365 days prior to surgery, %, median (IQR)			
Minimum	36.4 (30.0, 41.0)	35.6 (29.6, 39.9)	<.001
Maximum	41.4 (38.2, 44.5)	40.3 (37.1, 43.4)	<.001
Average	38.4 (34.0, 42.0)	37.5 (33.2, 41.0)	<.001
Variance	3.4 (0.0, 12.2)	3.4 (0.0, 11.7)	0.03
Count	1 (0, 4)	1 (0, 4)	<.001
The amount of hemoglobin relative to the size of the cell in blood within 7 days prior to surgery, g/dL, median (IQR)			

Features	UFH GNV	UFH JAX	P-value
Minimum	32.7 (31.7, 33.5)	32.8 (31.8, 33.7)	<.001
Maximum	33.5 (32.6, 34.2)	33.2 (32.3, 34.1)	<.001
Average	33.1 (32.2, 33.8)	33.0 (32.1, 33.9)	0.17
Variance	0.1 (0.0, 0.4)	0.0 (0.0, 0.2)	<.001
Count	2 (0, 2)	1 (0, 2)	<.001
The amount of hemoglobin relative to the size of the cell in blood within 8-365 days prior to surgery, g/dL, median (IQR)			
Minimum	32.2 (31.1, 33.2)	32.2 (31.1, 33.1)	0.20
Maximum	33.7 (32.9, 34.5)	33.4 (32.5, 34.3)	<.001
Average	33.0 (32.1, 33.7)	32.8 (31.9, 33.6)	<.001
Variance	0.3 (0.1, 0.6)	0.2 (0.0, 0.6)	<.001
Count	1 (0, 6)	1 (0, 4)	<.001
Red cell distribution width in Blood within 7 days prior to surgery, %, median (IQR)			
Minimum	14.1 (13.3, 15.1)	13.5 (12.8, 14.7)	<.001
Maximum	14.4 (13.5, 15.5)	13.7 (12.9, 15.0)	<.001
Average	14.2 (13.4, 15.3)	13.6 (12.8, 14.8)	<.001
Variance	0.0 (0.0, 0.0)	0.0 (0.0, 0.0)	<.001
Count	1 (0, 2)	1 (0, 2)	<.001
Red cell distribution width in Blood within 8-365 days prior to surgery, %, median (IQR)			
Minimum	13.8 (13.1, 14.7)	13.5 (12.8, 14.6)	<.001
Maximum	14.8 (13.8, 16.3)	14.5 (13.4, 16.1)	<.001
Average	14.3 (13.5, 15.4)	14.0 (13.2, 15.3)	<.001
Variance	0.1 (0.0, 0.6)	0.1 (0.0, 0.5)	0.04
Count	1 (0, 3)	1 (0, 4)	<.001
Platelet in blood within 7 days prior to surgery, thou/uL, median (IQR)			
Minimum	225 (177, 281)	237 (186, 294)	<.001
Maximum	242 (194, 303)	258 (207, 320)	<.001

Features	UFH GNV	UFH JAX	P-value
Average	233 (186, 291)	247 (197, 305)	<.001
Variance	0 (0, 242)	0 (0, 427)	<.001
Count	1 (0, 2)	1 (0, 2)	<.001
Platelet in blood within 8-365 days prior to surgery, thou/uL, median (IQR)			
Minimum	203 (155, 255)	217 (166, 270)	<.001
Maximum	266 (210, 345)	283 (227, 360)	<.001
Average	235 (189, 290)	251 (202, 304)	<.001
Variance	333 (0, 1850)	420 (0, 2025)	<.001
Count	1 (0, 4)	1 (0, 4)	<.001
Mean platelet volume within 7 days prior to surgery, fL, median (IQR)			
Minimum	7.9 (7.3, 8.7)	10.2 (9.6, 10.9)	<.001
Maximum	8.3 (7.6, 9.2)	10.4 (9.8, 11.1)	<.001
Average	8.1 (7.5, 8.9)	10.3 (9.7, 11.0)	<.001
Variance	0.0 (0.0, 0.1)	0.0 (0.0, 0.0)	<.001
Count	1 (0, 2)	1 (0, 2)	<.001
Mean platelet volume within 8-365 days prior to surgery, fL, median (IQR)			
Minimum	7.7 (7.1, 8.5)	9.9 (9.3, 10.7)	<.001
Maximum	8.9 (8.1, 9.8)	10.8 (10.1, 11.5)	<.001
Average	8.2 (7.6, 9.0)	10.3 (9.7, 11.0)	<.001
Variance	0.1 (0.0, 0.5)	0.1 (0.0, 0.2)	<.001
Count	1 (0, 3)	1 (0, 4)	<.001
Mean Corpuscular Volume in blood within 7 days prior to surgery, fL, median (IQR)			
Minimum	90.1 (86.1, 93.9)	88.6 (84.5, 92.3)	<.001
Maximum	90.8 (86.9, 94.7)	89.5 (85.4, 93.3)	<.001
Average	90.5 (86.5, 94.2)	89.0 (85.0, 92.7)	<.001
Variance	0.0 (0.0, 0.4)	0.0 (0.0, 0.7)	<.001
Count	1 (0, 2)	1 (0, 2)	<.001

Features	UFH GNV	UFH JAX	P-value
Mean Corpuscular Volume in blood within 8-365 days prior to surgery, fL, median (IQR)			
Minimum	89.0 (84.8, 92.9)	87.7 (83.2, 91.5)	<.001
Maximum	92.0 (88.0, 96.1)	90.8 (86.6, 94.9)	<.001
Average	90.4 (86.6, 94.2)	89.2 (85.1, 93.0)	<.001
Variance	0.8 (0.0, 3.3)	1.2 (0.0, 3.9)	<.001
Count	1 (0, 4)	1 (0, 4)	<.001
Mean Corpuscular Hemoglobin in blood within 7 days prior to surgery, fL, median (IQR)			
Minimum	29.8 (28.2, 31.2)	29.4 (27.7, 30.8)	<.001
Maximum	30.1 (28.6, 31.5)	29.7 (28.0, 31.1)	<.001
Average	29.9 (28.4, 31.3)	29.5 (27.8, 31.0)	<.001
Variance	0.0 (0.0, 0.1)	0.0 (0.0, 0.1)	<.001
Count	1 (0, 2)	1 (0, 2)	<.001
Mean Corpuscular Hemoglobin in blood within 8-365 days prior to surgery, fL, median (IQR)			
Minimum	29.3 (27.6, 30.8)	28.9 (26.9, 30.4)	<.001
Maximum	30.5 (28.9, 31.9)	29.9 (28.2, 31.4)	<.001
Average	29.9 (28.3, 31.3)	29.4 (27.6, 30.8)	<.001
Variance	0.1 (0.0, 0.5)	0.1 (0.0, 0.4)	0.39
Count	1 (0, 4)	1 (0, 4)	<.001
Serum Lactate within 7 days prior to surgery, mmol/L, median (IQR)			
Minimum	1.2 (0.9, 1.9)	1.6 (1.1, 2.3)	<.001
Maximum	1.6 (1.1, 2.6)	1.7 (1.2, 2.7)	<.001
Average	1.5 (1.0, 2.2)	1.7 (1.2, 2.5)	<.001
Variance	0.0 (0.0, 0.1)	0.0 (0.0, 0.0)	<.001
Count	0 (0, 0)	0 (0, 0)	<.001
Serum Lactate within 8-365 days prior to surgery, mmol/L, median (IQR)			
Minimum	0.9 (0.7, 1.3)	1.2 (0.9, 1.7)	<.001

Features	UFH GNV	UFH JAX	P-value
Maximum	1.7 (1.2, 2.7)	1.8 (1.2, 2.8)	<.001
Average	1.3 (1.0, 1.8)	1.5 (1.1, 2.2)	<.001
Variance	0.1 (0.0, 0.4)	0.0 (0.0, 0.4)	<.001
Count	0 (0, 0)	0 (0, 0)	<.001
Serum Alanine aminotransferase within 7 days prior to surgery, U/L, median (IQR)			
Minimum	17 (12, 28)	17 (11, 29)	0.38
Maximum	19 (12, 30)	19 (12, 33)	0.51
Average	18 (12, 29)	18 (12, 31)	0.92
Variance	0 (0, 0)	0 (0, 0)	<.001
Count	0 (0, 1)	0 (0, 1)	<.001
Serum Alanine aminotransferase within 8-365 days prior to surgery, U/L, median (IQR)			
Minimum	15 (10, 22)	14 (10, 22)	0.83
Maximum	22 (14, 38)	20 (14, 33)	<.001
Average	18 (13, 28)	17 (12, 27)	<.001
Variance	2 (0, 40)	1 (0, 24)	<.001
Count	0 (0, 1)	0 (0, 2)	<.001
Serum Albumin within 7 days prior to surgery, g/dL, median (IQR)			
Minimum	3.9 (3.3, 4.3)	3.6 (3.1, 4.1)	<.001
Maximum	4.0 (3.4, 4.3)	3.8 (3.3, 4.2)	<.001
Average	3.9 (3.3, 4.3)	3.7 (3.2, 4.1)	<.001
Variance	0.0 (0.0, 0.0)	0.0 (0.0, 0.0)	<.001
Count	0 (0, 1)	0 (0, 1)	<.001
Serum Albumin within 8-365 days prior to surgery, g/dL, median (IQR)			
Minimum	3.8 (3.2, 4.2)	3.9 (3.3, 4.2)	0.02
Maximum	4.2 (3.9, 4.5)	4.2 (3.9, 4.4)	<.001
Average	4.0 (3.6, 4.3)	4.0 (3.6, 4.3)	0.65
Variance	0.0 (0.0, 0.1)	0.0 (0.0, 0.1)	0.002
Count	0 (0, 1)	0 (0, 2)	<.001
Serum Aspartate aminotransferase within 7 days prior to surgery, U/L, median (IQR)			
Minimum	21 (16, 30)	20 (15, 30)	<.001

Features	UFH GNV	UFH JAX	P-value
Maximum	22 (17, 33)	22 (16, 35)	<.001
Average	22 (16, 32)	21 (15, 33)	<.001
Variance	0 (0, 0)	0 (0, 0)	<.001
Count	0 (0, 1)	0 (0, 1)	<.001
Serum Aspartate aminotransferase within 8-365 days prior to surgery, U/L, median (IQR)			
Minimum	18 (14, 23)	17 (13, 23)	<.001
Maximum	24 (18, 38)	22 (17, 33)	0.01
Average	21 (16, 29)	20 (15, 27)	<.001
Variance	2 (0, 41)	2 (0, 24)	<.001
Count	0 (0, 1)	0 (0, 2)	<.001
Serum Bilirubin direct within 7 days prior to surgery, mg/dL, median (IQR)			
Minimum	0.2 (0.2, 0.2)	0.1 (0.1, 0.2)	<.001
Maximum	0.2 (0.2, 0.2)	0.2 (0.1, 0.2)	<.001
Average	0.2 (0.2, 0.2)	0.2 (0.1, 0.2)	<.001
Variance	0.0 (0.0, 0.0)	0.0 (0.0, 0.0)	0.09
Count	0 (0, 0)	0 (0, 0)	<.001
Serum Bilirubin direct within 8-365 days prior to surgery, mg/dL, median (IQR)			
Minimum	0.2 (0.1, 0.2)	0.1 (0.1, 0.2)	<.001
Maximum	0.2 (0.2, 0.2)	0.1 (0.1, 0.2)	<.001
Average	0.2 (0.2, 0.2)	0.1 (0.1, 0.2)	<.001
Variance	0.0 (0.0, 0.0)	0.0 (0.0, 0.0)	<.001
Count	0 (0, 0)	0 (0, 0)	<.001
Serum C reactive protein within 7 days prior to surgery, mg/L, median (IQR)			
Minimum	35.5 (6.4, 107.4)	58.6 (12.0, 144.2)	<.001
Maximum	39.4 (6.8, 114.4)	60.6 (12.3, 147.6)	<.001
Average	38.3 (6.7, 111.0)	60.1 (12.2, 144.9)	<.001
Variance	0.0 (0.0, 0.0)	0.0 (0.0, 0.0)	0.005
Count	0 (0, 0)	0 (0, 0)	<.001
Serum C reactive protein within 8-365 days prior to surgery, mg/L, median (IQR)			

Features	UFH GNV	UFH JAX	P-value
Minimum	9.2 (2.6, 44.8)	11.7 (3.4, 55.2)	<.001
Maximum	34.9 (5.3, 117.5)	26.6 (5.3, 104.3)	0.01
Average	23.8 (4.7, 79.1)	19.3 (4.8, 82.0)	0.86
Variance	0.0 (0.0, 521.7)	0.0 (0.0, 20.6)	<.001
Count	0 (0, 0)	0 (0, 0)	<.001
Serum INR within 7 days prior to surgery, median (IQR)			
Minimum	1.1 (1.0, 1.2)	1.1 (1.0, 1.1)	<.001
Maximum	1.1 (1.0, 1.2)	1.1 (1.0, 1.2)	<.001
Average	1.1 (1.0, 1.2)	1.1 (1.0, 1.2)	<.001
Variance	0.0 (0.0, 0.0)	0.0 (0.0, 0.0)	<.001
Count	0 (0, 1)	1 (0, 1)	<.001
Serum INR within 8-365 days prior to surgery, median (IQR)			
Minimum	1.0 (1.0, 1.1)	1.0 (1.0, 1.1)	<.001
Maximum	1.1 (1.0, 1.3)	1.1 (1.0, 1.2)	<.001
Average	1.1 (1.0, 1.2)	1.1 (1.0, 1.2)	<.001
Variance	0.0 (0.0, 0.0)	0.0 (0.0, 0.0)	0.09
Count	0 (0, 1)	0 (0, 1)	0.03
Erythrocyte sedimentation rate within 7 days prior to surgery, mm/h, median (IQR)			
Minimum	47 (22, 83)	56 (28, 86)	<.001
Maximum	48 (23, 85)	57 (29, 87)	<.001
Average	48 (22, 84)	56 (29, 87)	<.001
Variance	0 (0, 0)	0 (0, 0)	0.06
Count	0 (0, 0)	0 (0, 0)	<.001
Erythrocyte sedimentation rate within 8-365 days prior to surgery, mm/h, median (IQR)			
Minimum	28 (11, 55)	31 (13, 66)	<.001
Maximum	40 (17, 78)	42 (16, 86)	0.12
Average	35 (16, 65)	39 (15, 76)	.004
Variance	0 (0, 53)	0 (0, 40)	0.33
Count	0 (0, 0)	0 (0, 0)	<.001
Serum Troponin I within 7 days prior to surgery, ng/mL, median (IQR)			

Features	UFH GNV	UFH JAX	P-value
Minimum	0.03 (0.03, 0.1)	0.05 (0.05, 0.05)	<.001
Maximum	0.04 (0.03, 0.1)	0.05 (0.05, 0.05)	<.001
Average	0.04 (0.03, 0.1)	0.05 (0.05, 0.05)	<.001
Variance	0.0 (0.0, 0.0)	0.0 (0.0, 0.0)	<.001
Count	0 (0, 0)	0 (0, 0)	<.001
Serum Troponin I within 8-365 days prior to surgery, ng/mL, median (IQR)			
Minimum	0.03 (0.03, 0.1)	0.05 (0.05, 0.05)	<.001
Maximum	0.04 (0.03, 0.1)	0.05 (0.05, 0.05)	<.001
Average	0.04 (0.03, 0.1)	0.05 (0.05, 0.05)	<.001
Variance	0.0 (0.0, 0.0)	0.0 (0.0, 0.0)	<.001
Count	0 (0, 0)	0 (0, 0)	<.001
Serum Troponin T within 7 days prior to surgery, ng/mL, median (IQR)			
Minimum	0.03 (0.02, 0.03)	0.01 (0.01, 0.01)	<.001
Maximum	0.03 (0.03, 0.03)	0.01 (0.01, 0.03)	<.001
Average	0.03 (0.03, 0.03)	0.01 (0.01, 0.02)	<.001
Variance	0.0 (0.0, 0.0)	0.0 (0.0, 0.0)	<.001
Count	0 (0, 0)	0 (0, 0)	<.001
Serum Troponin T within 8-365 days prior to surgery, ng/mL, median (IQR)			
Minimum	0.03 (0.01, 0.03)	0.01 (0.01, 0.01)	<.001
Maximum	0.03 (0.03, 0.03)	0.01 (0.01, 0.02)	<.001
Average	0.03 (0.03, 0.03)	0.01 (0.01, 0.01)	<.001
Variance	0.0 (0.0, 0.0)	0.0 (0.0, 0.0)	<.001
Count	0 (0, 0)	0 (0, 0)	<.001
Reference estimated glomerular filtration rate, median (IQR)	96.6 (83.8, 108.2)	99.8 (86.2, 110.8)	<.001
Reference serum creatinine, mg/dL, median (IQR)	0.8 (0.7, 1.0)	0.8 (0.7, 1.0)	0.008

Abbreviation: SD, standard deviation; IQR, interquartile range; OB, obstetrician; ACE, Angiotensin-converting enzyme.
[a] Race and ethnicity were self-reported.
[b] Other races include American Indian or Alaska Native, Asian, Native Hawaiian or Pacific Islander, and multiracial.
[c] Other surgery type includes plastic surgery, burn surgery, pediatric surgery, transplantation, ophthalmology, medicine gastroenterology, and interventional cardiology.

SDC Table 2. Patient characteristics

Variables	UFH GNV			UFH JAX		
	Training	Validation	Test	Training	Validation	Test
Number of patients, n	39,582	4,397	18,848	14,846	1,649	7,068
Number of encounters, n	51,953	5,565	22,332	18,502	2,023	8,111
Age in years, mean (SD)[b]	57 (17)	57 (17)	58 (17)[a]	52 (17)	53 (17)[a]	53 (17)[a]
Sex, n (%)[b]						
Male	19,884 (50)	2,220 (50)	9,420 (50)	7,642 (51)	863 (52)	3,671 (52)
Female	19,698 (50)	2,177 (50)	9,428 (50)	7,204 (49)	786 (48)	3,397 (48)
Race, n (%)[b,c]						
White	30,947 (78)	3,490 (80)	14,677 (78)	8,675 (59)	972 (59)	4,133 (58)
African American	5,474 (14)	583 (13)	2,628 (14)	5,158 (35)	560 (34)	2,407 (34)
Other[d]	2,521 (6)	264 (6)	1,216 (6)	952 (6)	113 (7)	484 (7)
Missing	640 (2)	60 (1)	327 (2)	61 (0)	4 (0)	44 (1)
Ethnicity, n (%)[b,c]						
Non-Hispanic	37,165 (94)	4,078 (93)[a]	17,421 (93)[a]	14,079 (95)	1,559(95)	6,639 (94)[a]
Hispanic	1,707 (4)	254 (6)[a]	980 (5)[a]	676 (5)	85 (5)	376 (5)[a]
Missing	710 (2)	65 (1)	447 (2)[a]	91 (0)	5 (0)	53 (1)
Marital Status, n (%)[b]						
Married	19,182 (48)	2,001(45)[a]	8,815(47)[a]	5,055 (34)	566 (34)	2,577 (37)[a]
Single	11,769 (30)	1,256 (29)	5,333 (28)[a]	5,392 (36)	591 (36)	2,492 (35)
Divorced	6,084 (15)	596 (13)[a]	2,630(14)[a]	3,875 (26)	437 (26)	1,776 (25)
Missing	2,547 (7)	544 (13)[a]	2,070 (11)[a]	524 (4)	55 (4)	223 (3)
Insurance, n (%)[b]						
Medicare	17,767 (45)	1,987 (45)	8,841 (47)[a]	4,408 (30)	517 (31)	2,208 (31)
Private	12,311 (31)	1,288 (29)[a]	5,497 (29)[a]	4,308 (29)	492 (30)	2,181 (31)[a]
Medicaid	6,296 (16)	731 (17)	2,894(15)	5,908 (40)	598 (36)[a]	2,499 (35)[a]
Uninsured	3,208 (8)	391 (9)	1616 (9)	222 (1)	42 (3)[a]	180 (3)[a]
Complications, n (%)[e]						

Variables	UFH GNV			UFH JAX		
	Training	Validation	Test	Training	Validation	Test
Acute kidney injury	7,924 (15)	932 (17)[a]	3,917 (18)[a]	2,577 (14)	266 (13)	1,048 (13)
Cardiovascular complications	6,419 (12)	878 (16)[a]	3,911 (18)[a]	2,104 (11)	234 (12)	788 (10)[a]
Neurological complications, including delirium	8,873 (17)	1,178 (21)[a]	5,135 (23)[a]	2,199 (12)	240 (12)	973 (12)
Prolonged ICU stay	15,049 (29)	1,610 (29)	7,657 (34)[a]	4,372 (24)	512 (25)	2,013 (25)
Prolonged mechanical ventilation	4,667 (9)	461 (8)	1,833 (8)[a]	1,492 (8)	135 (7)	489 (6)[a]
Sepsis	3,958 (8)	542 (10)[a]	2,195 (10)[a]	1,601 (9)	202 (10)	669 (8)
Venous thromboembolism	2,483 (5)	310 (6)[a]	1,423 (6)[a]	721 (4)	69 (3)	255 (3)[a]
Wound complications	8,088 (16)	1,174 (21)[a]	5,037 (23)[a]	2,594 (14)	290 (14)	1,107 (14)
In-hospital mortality	952 (2)	93 (2)	383 (2)	329 (2)	27 (1)	97 (1)[a]

Abbreviation: ICU, intensive care unit.
[a] p<=0.05 comparing validation to training data for each site.
[b] Data were reported based on values calculated at the latest hospital admission.
[c] Race and ethnicity were self-reported.
[d] Other races include American Indian or Alaska Native, Asian, Native Hawaiian or Pacific Islander, and multiracial.
[e] Data were reported based on postoperative complication status for each surgical procedure.

SDC Table 3. Comparison of AUPRC for local learning and federated learning models

		Preoperative models		Perioperative models	
**Outcome**	**Model**	**GNV test data**	**JAX test data**	**GNV test data**	**JAX test data**
Prolonged ICU stay	GNV Model	0.83 (0.83-0.84)	0.62 (0.60-0.63)	**0.87 (0.87-0.88)**	0.71 (0.69-0.73)
	JAX Model	0.58 (0.57-0.59)	**0.76 (0.74-0.78)**	0.80 (0.79-0.80)	**0.80 (0.78-0.81)**
	SCAFFOLD	**0.84 (0.83-0.84)**	0.71 (0.70-0.73)	**0.87 (0.87-0.88)**	0.76 (0.74-0.78)
Sepsis	GNV Model	0.53 (0.51-0.55)	0.47 (0.44-0.51)	**0.56 (0.54-0.57)**	0.51 (0.48-0.54)
	JAX Model	0.42 (0.40-0.44)	**0.52 (0.49-0.55)**	0.44 (0.42-0.46)	**0.52 (0.49-0.56)**
	SCAFFOLD	**0.54 (0.52-0.56)**	0.50 (0.46-0.53)	**0.56 (0.54-0.58)**	0.51 (0.48-0.55)
Cardiovascular complication	GNV Model	0.52 (0.50-0.53)	0.28 (0.26-0.31)	0.58 (0.57-0.60)	0.41 (0.38-0.44)
	JAX Model	0.34 (0.33-0.36)	0.36 (0.34-0.39)	0.54 (0.53-0.60)	**0.43 (0.40-0.46)**
	SCAFFOLD	**0.53 (0.51-0.54)**	0.34 (0.32-0.37)	**0.59 (0.58-0.60)**	**0.43 (0.40-0.46)**
Venous thromboembolism	GNV Model	0.25 (0.23-0.27)	0.12 (0.10-0.15)	0.27 (0.25-0.29)	0.14 (0.11-0.17)
	JAX Model	0.16 (0.15-0.18)	0.13 (0.11-0.16)	0.19 (0.18-0.21)	0.14 (0.12-0.17)
	SCAFFOLD	**0.27 (0.25-0.29)**	**0.14 (0.11-0.18)**	**0.29 (0.27-0.31)**	0.14 (0.12-0.18)
Prolonged mechanical ventilation	GNV Model	0.53 (0.51-0.55)	0.30 (0.27-0.34)	0.61 (0.59-0.62)	0.41 (0.38-0.45)
	JAX Model	0.40 (0.38-0.42)	**0.41 (0.37-0.45)**	0.49 (0.47-0.51)	**0.51 (0.47-0.55)**
	SCAFFOLD	**0.56 (0.54-0.58)**	0.40 (0.36-0.44)	**0.62 (0.60-0.63)**	0.49 (0.45-0.53)
Neurological complications, including delirium	GNV Model	0.66 (0.65-0.68)	0.43 (0.40-0.46)	0.68 (0.67-0.69)	0.46 (0.43-0.48)
	JAX Model	0.47 (0.46-0.49)	0.43 (0.41-0.46)	0.52 (0.51-0.53)	0.46 (0.43-0.48)
	SCAFFOLD	**0.68 (0.67-0.69)**	**0.48 (0.46-0.51)**	**0.69 (0.68-0.70)**	**0.49 (0.46-0.52)**
Wound complications	GNV Model	0.58 (0.57-0.59)	0.32 (0.30-0.34)	0.60 (0.59-0.61)	0.34 (0.32-0.37)
	JAX Model	0.38 (0.37-0.39)	0.31 (0.29-0.33)	0.42 (0.41-0.43)	0.32 (0.30-0.35)
	SCAFFOLD	**0.60 (0.58-0.61)**	**0.37 (0.34-0.39)**	**0.61 (0.60-0.62)**	**0.39 (0.37-0.42)**
Acute kidney injury	GNV Model	0.52 (0.51-0.54)	0.40 (0.37-0.42)	**0.55 (0.53-0.56)**	0.43 (0.40-0.45)
	JAX Model	0.33 (0.32-0.34)	0.38 (0.36-0.41)	0.45 (0.44-0.46)	0.42 (0.39-0.44)
	SCAFFOLD	**0.53**	**0.41**	**0.55**	**0.44**

		Preoperative models		Perioperative models	
**Outcome**	**Model**	**GNV test data**	**JAX test data**	**GNV test data**	**JAX test data**
		**(0.51-0.54)**	**(0.38-0.43)**	**(0.54-0.57)**	**(0.41-0.46)**
In-hospital mortality	GNV Model	0.16 (0.14-0.19)	0.10 (0.07-0.15)	**0.20 (0.17-0.23)**	0.19 (0.14-0.26)
	JAX Model	0.13 (0.12-0.16)	**0.14 (0.10-0.20)**	0.16 (0.13-0.19)	0.20 (0.15-0.28)
	SCAFFOLD	**0.17 (0.14-0.19)**	**0.14 (0.10-0.19)**	**0.20 (0.17-0.24)**	**0.22 (0.17-0.30)**

Bold value indicates the best performance.

Abbreviation: ICU, intensive care unit.

SDC Table 4. Subgroup analysis of AUROC with 95% confidence interval for federated learning models based on sex in the UFH GNV cohort

		CL		SCAFFOLD		FedAvg		FedProx	
**Outcome**	**Period**	**Female**	**Male**	**Female**	**Male**	**Female**	**Male**	**Female**	**Male**
Prolonged ICU stay	PreOp	0.89 (0.89-0.90)	0.89 (0.89-0.90)	0.90 (0.90-0.91)	0.90 (0.89-0.90)	0.90 (0.90-0.91)	0.90 (0.89-0.90)	0.90 (0.90-0.91)	0.90 (0.89-0.90)
	PeriOp	0.92 (0.91-0.92)	0.91 (0.90-0.91)	0.92 (0.92-0.93)	0.92 (0.91-0.92)	0.92 (0.92-0.93)	0.92 (0.91-0.92)	0.92 (0.92-0.93)	0.91 (0.91-0.92)
Sepsis	PreOp	0.89 (0.88-0.90)	0.87 (0.86-0.88)[a]	0.89 (0.88-0.90)	0.87 (0.86-0.87)[a]	0.89 (0.88-0.90)	0.86 (0.85-0.87)[a]	0.89 (0.88-0.90)	0.87 (0.86-0.88)[a]
	PeriOp	0.89 (0.89-0.90)	0.88 (0.87-0.88)[a]	0.90 (0.89-0.91)	0.88 (0.87-0.88)[a]	0.90 (0.89-0.91)	0.88 (0.87-0.88)[a]	0.90 (0.89-0.91)	0.88 (0.87-0.89)[a]
Cardiovascular complication	PreOp	0.81 (0.80-0.82)	0.82 (0.82-0.83)[a]	0.81 (0.80-0.82)	0.82 (0.82-0.83)[a]	0.81 (0.80-0.81)	0.82 (0.81-0.83)[a]	0.81 (0.80-0.82)	0.83 (0.82-0.84)[a]
	PeriOp	0.84 (0.83-0.85)	0.85 (0.85-0.86)	0.84 (0.84-0.85)	0.85 (0.85-0.86)	0.84 (0.84-0.85)	0.86 (0.85-0.86)	0.84 (0.84-0.85)	0.86 (0.85-0.87)[a]
Venous thromboembolism	PreOp	0.85 (0.84-0.86)	0.81 (0.80-0.82)[a]	0.85 (0.84-0.87)	0.81 (0.80-0.82)[a]	0.85 (0.84-0.86)	0.80 (0.79-0.81)[a]	0.85 (0.84-0.86)	0.81 (0.80-0.83)[a]
	PeriOp	0.86 (0.84-0.87)	0.81 (0.80-0.82)[a]	0.86 (0.85-0.87)	0.82 (0.80-0.83)[a]	0.86 (0.85-0.87)	0.82 (0.80-0.83)[a]	0.86 (0.84-0.87)	0.82 (0.81-0.83)[a]
Prolonged mechanical ventilation	PreOp	0.90 (0.89-0.91)	0.90 (0.89-0.90)	0.90 (0.89-0.91)	0.90 (0.89-0.91)	0.90 (0.89-0.91)	0.90 (0.89-0.90)	0.90 (0.89-0.91)	0.90 (0.89-0.91)
	PeriOp	0.91 (0.90-0.92)	0.91 (0.91-0.92)	0.92 (0.91-0.93)	0.92 (0.91-0.92)	0.92 (0.91-0.93)	0.92 (0.91-0.93)	0.92 (0.91-0.93)	0.92 (0.91-0.93)
Neurological complications, including delirium	PreOp	0.85 (0.84-0.86)	0.85 (0.84-0.85)	0.86 (0.85-0.86)	0.85 (0.84-0.86)	0.85 (0.85-0.86)	0.85 (0.84-0.86)	0.86 (0.85-0.86)	0.85 (0.84-0.86)
	PeriOp	0.85 (0.84-0.86)	0.85 (0.84-0.86)	0.86 (0.85-0.87)	0.86 (0.85-0.86)	0.86 (0.85-0.87)	0.86 (0.85-0.86)	0.86 (0.85-0.87)	0.85 (0.85-0.86)
Wound complications	PreOp	0.79 (0.79-0.80)	0.79 (0.78-0.79)	0.80 (0.79-0.81)	0.79 (0.78-0.80)	0.79 (0.79-0.80)	0.78 (0.78-0.79)	0.80 (0.79-0.81)	0.79 (0.78-0.80)
	PeriOp	0.80 (0.79-0.81)	0.79 (0.79-0.80)	0.81 (0.80-0.82)	0.80 (0.79-0.81)	0.81 (0.80-0.82)	0.80 (0.79-0.81)	0.81 (0.80-0.82)	0.80 (0.79-0.81)
Acute kidney injury	PreOp	0.82 (0.81-0.83)	0.81 (0.80-0.82)	0.83 (0.82-0.84)	0.81 (0.81-0.82)	0.82 (0.81-0.83)	0.81 (0.80-0.82)	0.83 (0.82-0.84)	0.81 (0.81-0.82)
	PeriOp	0.82	0.82	0.84	0.83	0.83	0.83	0.83	0.82

		CL		SCAFFOLD		FedAvg		FedProx	
**Outcome**	**Period**	**Female**	**Male**	**Female**	**Male**	**Female**	**Male**	**Female**	**Male**
		(0.82-0.83)	(0.81-0.83)	(0.83-0.84)	(0.82-0.83)	(0.82-0.84)	(0.82-0.83)	(0.82-0.84)	(0.82-0.83)
In-hospital mortality	PreOp	0.90 (0.88-0.92)	0.90 (0.88-0.91)	0.89 (0.87-0.91)	0.88 (0.86-0.90)	0.89 (0.87-0.91)	0.88 (0.86-0.90)	0.90 (0.89-0.92)	0.89 (0.88-0.91)
	PeriOp	0.90 (0.89-0.92)	0.90 (0.88-0.91)	0.91 (0.89-0.92)	0.90 (0.89-0.92)	0.92 (0.90-0.93)	0.91 (0.89-0.92)	0.91 (0.89-0.92)	0.91 (0.89-0.92)

[a] p<=0.05 comparing female to male patients for each model.
Abbreviation: CL, central learning; ICU, intensive care unit; PreOp, preoperative; PeriOp, perioperative.

SDC Table 5. Subgroup analysis of AUROC with 95% confidence interval for federated learning models based on sex in the UFH JAX cohort

		CL		SCAFFOLD		FedAvg		FedProx	
**Outcome**	**Period**	**Female**	**Male**	**Female**	**Male**	**Female**	**Male**	**Female**	**Male**
Prolonged ICU stay	PreOp	0.89 (0.88-0.90)	0.89 (0.88-0.90)	0.86 (0.85-0.87)	0.86 (0.85-0.87)	0.87 (0.86-0.88)	0.87 (0.86-0.88)	0.86 (0.85-0.88)	0.86 (0.85-0.87)
	PeriOp	0.90 (0.89-0.91)	0.91 (0.90-0.92)	0.89 (0.88-0.90)	0.89 (0.89-0.90)	0.88 (0.87-0.89)	0.89 (0.88-0.90)	0.88 (0.87-0.89)	0.89 (0.88-0.90)
Sepsis	PreOp	0.91 (0.89-0.92)	0.87 (0.85-0.89)[a]	0.90 (0.88-0.92)	0.86 (0.85-0.88)[a]	0.90 (0.89-0.92)	0.86 (0.84-0.87)[a]	0.90 (0.88-0.92)	0.86 (0.84-0.87)[a]
	PeriOp	0.92 (0.91-0.93)	0.88 (0.86-0.89)[a]	0.91 (0.90-0.93)	0.87 (0.85-0.89)[a]	0.91 (0.90-0.93)	0.87 (0.85-0.88)[a]	0.91 (0.89-0.92)	0.87 (0.85-0.89)[a]
Cardiovascular complication	PreOp	0.77 (0.75-0.79)	0.82 (0.81-0.84)[a]	0.76 (0.74-0.78)	0.81 (0.80-0.83)[a]	0.76 (0.74-0.78)	0.81 (0.79-0.83)[a]	0.76 (0.74-0.78)	0.80 (0.79-0.82)[a]
	PeriOp	0.82 (0.80-0.84)	0.86 (0.84-0.87)[a]	0.82 (0.80-0.84)	0.86 (0.84-0.87)[a]	0.82 (0.80-0.84)	0.86 (0.84-0.87)[a]	0.81 (0.79-0.84)	0.85 (0.84-0.87)[a]
Venous thromboembolism	PreOp	0.84 (0.81-0.87)	0.78 (0.75-0.81)[a]	0.83 (0.79-0.86)	0.75 (0.72-0.78)[a]	0.84 (0.80-0.87)	0.76 (0.74-0.79)[a]	0.83 (0.80-0.86)	0.76 (0.73-0.78)[a]
	PeriOp	0.86 (0.83-0.89)	0.80 (0.77-0.82)[a]	0.85 (0.81-0.88)	0.80 (0.78-0.83)	0.86 (0.83-0.88)	0.79 (0.76-0.81)[a]	0.85 (0.82-0.88)	0.79 (0.76-0.81)[a]
Prolonged mechanical ventilation	PreOp	0.84 (0.81-0.87)	0.84 (0.81-0.86)	0.84 (0.81-0.87)	0.83 (0.81-0.85)	0.84 (0.81-0.86)	0.83 (0.81-0.85)	0.84 (0.81-0.87)	0.81 (0.79-0.83)
	PeriOp	0.86 (0.83-0.88)	0.86 (0.84-0.88)	0.86 (0.83-0.89)	0.86 (0.84-0.88)	0.85 (0.82-0.88)	0.85 (0.83-0.87)	0.85 (0.83-0.88)	0.85 (0.83-0.87)
Neurological complications, including delirium	PreOp	0.85 (0.84-0.87)	0.83 (0.82-0.85)	0.85 (0.83-0.87)	0.83 (0.81-0.84)	0.84 (0.83-0.86)	0.83 (0.81-0.84)	0.84 (0.83-0.86)	0.82 (0.80-0.83)
	PeriOp	0.86 (0.85-0.88)	0.83 (0.82-0.85)[a]	0.85 (0.84-0.87)	0.83 (0.82-0.84)	0.84 (0.83-0.86)	0.83 (0.82-0.85)	0.86 (0.84-0.87)	0.83 (0.81-0.84)[a]
Wound complications	PreOp	0.71 (0.69-0.73)	0.72 (0.70-0.74)	0.70 (0.68-0.73)	0.71 (0.69-0.73)	0.71 (0.69-0.73)	0.71 (0.69-0.72)	0.71 (0.69-0.73)	0.71 (0.69-0.73)
	PeriOp	0.73 (0.71-0.75)	0.73 (0.72-0.75)	0.73 (0.71-0.75)	0.74 (0.72-0.76)	0.72 (0.70-0.75)	0.73 (0.72-0.75)	0.73 (0.71-0.75)	0.73 (0.71-0.75)
Acute kidney injury	PreOp	0.82 (0.80-0.83)	0.78 (0.76-0.79)[a]	0.81 (0.79-0.82)	0.77 (0.76-0.79)[a]	0.81 (0.79-0.83)	0.77 (0.76-0.79)[a]	0.81 (0.79-0.83)	0.78 (0.76-0.79)[a]
	PeriOp	0.83	0.79	0.82	0.80	0.82	0.80	0.82	0.79

		CL		SCAFFOLD		FedAvg		FedProx	
**Outcome**	**Period**	**Female**	**Male**	**Female**	**Male**	**Female**	**Male**	**Female**	**Male**
		(0.81-0.84)	(0.78-0.81)[a]	(0.81-0.84)	(0.78-0.81)	(0.80-0.83)	(0.78-0.81)	(0.80-0.83)	(0.77-0.81)
In-hospital mortality	PreOp	0.95 (0.92-0.97)	0.90 (0.87-0.92)[a]	0.94 (0.91-0.97)	0.87 (0.84-0.90)[a]	0.93 (0.90-0.96)	0.87 (0.84-0.90)[a]	0.93 (0.89-0.96)	0.88 (0.84-0.91)
	PeriOp	0.96 (0.92-0.98)	0.91 (0.88-0.93)[a]	0.98 (0.96-0.99)	0.89 (0.85-0.92)[a]	0.97 (0.95-0.98)	0.89 (0.86-0.92)[a]	0.97 (0.96-0.98)	0.90 (0.87-0.93)[a]

[a] p<=0.05 comparing female to male patients for each model.

Abbreviation: CL, central learning; ICU, intensive care unit; PreOp, preoperative; PeriOp, perioperative.

SDC Table 6. Subgroup analysis of AUROC with 95% confidence interval for federated learning models based on race in the UFH GNV cohort

		CL		SCAFFOLD		FedAvg		FedProx	
**Outcome**	**Period**	**African American**	**Non-African American**	**African American**	**Non-African American**	**African American**	**Non-African American**	**African American**	**Non-African American**
Prolonged ICU stay	PreOp	0.90 (0.89-0.91)	0.89 (0.89-0.90)	0.90 (0.89-0.91)	0.90 (0.90-0.91)	0.90 (0.89-0.91)	0.90 (0.90-0.90)	0.90 (0.89-0.91)	0.90 (0.90-0.91)
	PeriOp	0.91 (0.90-0.92)	0.91 (0.91-0.92)	0.92 (0.91-0.92)	0.92 (0.92-0.92)	0.91 (0.91-0.92)	0.92 (0.92-0.92)	0.91 (0.91-0.92)	0.92 (0.92-0.92)
Sepsis	PreOp	0.90 (0.88-0.91)	0.87 (0.87-0.88)[a]	0.90 (0.88-0.91)	0.87 (0.87-0.88)[a]	0.90 (0.88-0.91)	0.87 (0.86-0.88)[a]	0.90 (0.88-0.91)	0.88 (0.87-0.88)[a]
	PeriOp	0.90 (0.89-0.92)	0.88 (0.88-0.89)[a]	0.91 (0.90-0.92)	0.88 (0.87-0.89)[a]	0.91 (0.89-0.92)	0.88 (0.88-0.89)[a]	0.91 (0.89-0.92)	0.88 (0.88-0.89)[a]
Cardiovascular complication	PreOp	0.82 (0.81-0.84)	0.81 (0.81-0.82)	0.82 (0.81-0.84)	0.82 (0.81-0.82)	0.82 (0.81-0.84)	0.81 (0.81-0.82)	0.82 (0.81-0.84)	0.82 (0.81-0.82)
	PeriOp	0.85 (0.83-0.87)	0.85 (0.84-0.85)	0.85 (0.84-0.87)	0.85 (0.84-0.86)	0.85 (0.84-0.87)	0.85 (0.85-0.86)	0.85 (0.83-0.87)	0.85 (0.85-0.86)
Venous thromboembolism	PreOp	0.84 (0.82-0.86)	0.83 (0.82-0.84)	0.85 (0.83-0.87)	0.83 (0.82-0.84)	0.84 (0.82-0.86)	0.82 (0.81-0.83)	0.85 (0.83-0.87)	0.83 (0.82-0.84)
	PeriOp	0.84 (0.82-0.86)	0.83 (0.82-0.84)	0.85 (0.82-0.87)	0.84 (0.83-0.85)	0.84 (0.82-0.86)	0.84 (0.83-0.85)	0.85 (0.83-0.87)	0.84 (0.83-0.85)
Prolonged mechanical ventilation	PreOp	0.90 (0.88-0.91)	0.90 (0.89-0.91)	0.90 (0.88-0.92)	0.90 (0.90-0.91)	0.89 (0.88-0.91)	0.90 (0.89-0.91)	0.90 (0.88-0.92)	0.90 (0.90-0.91)
	PeriOp	0.91 (0.89-0.92)	0.92 (0.91-0.92)	0.91 (0.89-0.92)	0.92 (0.91-0.92)	0.91 (0.90-0.93)	0.92 (0.92-0.93)	0.91 (0.89-0.92)	0.92 (0.92-0.93)
Neurological complications, including delirium	PreOp	0.86 (0.85-0.87)	0.85 (0.84-0.85)	0.87 (0.86-0.88)	0.85 (0.85-0.86)	0.86 (0.85-0.88)	0.85 (0.85-0.86)	0.87 (0.85-0.88)	0.85 (0.85-0.86)
	PeriOp	0.86 (0.85-0.87)	0.85 (0.85-0.86)	0.87 (0.86-0.88)	0.86 (0.85-0.86)	0.87 (0.86-0.88)	0.86 (0.85-0.86)	0.87 (0.86-0.88)	0.86 (0.85-0.86)
Wound complications	PreOp	0.77 (0.76-0.79)	0.79 (0.79-0.80)	0.78 (0.77-0.80)	0.80 (0.79-0.80)	0.78 (0.76-0.80)	0.79 (0.78-0.80)	0.79 (0.78-0.81)	0.80 (0.79-0.80)
	PeriOp	0.79 (0.77-0.81)	0.80 (0.79-0.81)	0.80 (0.78-0.81)	0.81 (0.80-0.82)	0.79 (0.78-0.81)	0.81 (0.80-0.81)	0.80 (0.78-0.81)	0.81 (0.80-0.82)
	PreOp	0.81	0.82	0.81	0.82	0.82	0.82	0.82	0.82

		CL		SCAFFOLD		FedAvg		FedProx	
**Outcome**	**Period**	**African American**	**Non-African American**	**African American**	**Non-African American**	**African American**	**Non-African American**	**African American**	**Non-African American**
Acute kidney injury		(0.79-0.82)	(0.81-0.82)	(0.80-0.83)	(0.82-0.83)	(0.80-0.83)	(0.81-0.83)	(0.80-0.83)	(0.82-0.83)
	PeriOp	0.81 (0.80-0.83)	0.82 (0.82-0.83)	0.82 (0.81-0.84)	0.83 (0.83-0.84)	0.83 (0.81-0.84)	0.83 (0.82-0.84)	0.82 (0.81-0.84)	0.83 (0.82-0.84)
In-hospital mortality	PreOp	0.93 (0.91-0.95)	0.89 (0.88-0.91)[a]	0.92 (0.89-0.94)	0.88 (0.87-0.90)[a]	0.90 (0.86-0.93)	0.88 (0.87-0.90)	0.92 (0.90-0.95)	0.90 (0.88-0.91)
	PeriOp	0.93 (0.91-0.95)	0.90 (0.89-0.91)[a]	0.93 (0.91-0.95)	0.90 (0.89-0.91)	0.93 (0.91-0.95)	0.91 (0.90-0.92)	0.93 (0.91-0.95)	0.90 (0.89-0.91)

[a] $p<=0.05$ comparing African American to Non-African American patients for each model.
Abbreviation: CL, central learning; ICU, intensive care unit; PreOp, preoperative; PeriOp, perioperative.

SDC Table 7. Subgroup analysis of AUROC with 95% confidence interval for federated learning models based on race in the UFH JAX cohort

		CL	CL	SCAFFOLD	SCAFFOLD	FedAvg	FedAvg	FedProx	FedProx
**Outcome**	**Period**	**African American**	**Non-African American**	**African American**	**Non-African American**	**African American**	**Non-African American**	**African American**	**Non-African American**
Prolonged ICU stay	PreOp	0.90 (0.89-0.91)	0.89 (0.88-0.90)	0.87 (0.86-0.88)	0.87 (0.86-0.87)	0.87 (0.86-0.88)	0.88 (0.87-0.88)	0.87 (0.85-0.88)	0.87 (0.86-0.88)
	PeriOp	0.91 (0.90-0.92)	0.90 (0.90-0.91)	0.90 (0.89-0.91)	0.89 (0.88-0.90)	0.89 (0.88-0.90)	0.88 (0.88-0.89)	0.90 (0.88-0.91)	0.88 (0.88-0.89)
Sepsis	PreOp	0.90 (0.88-0.91)	0.88 (0.87-0.90)	0.89 (0.87-0.91)	0.88 (0.86-0.89)	0.89 (0.87-0.91)	0.87 (0.85-0.89)	0.88 (0.86-0.90)	0.87 (0.86-0.89)
	PeriOp	0.90 (0.89-0.92)	0.89 (0.88-0.91)	0.90 (0.88-0.91)	0.88 (0.87-0.90)	0.90 (0.88-0.91)	0.88 (0.87-0.90)	0.89 (0.88-0.91)	0.88 (0.87-0.90)
Cardiovascular complication	PreOp	0.80 (0.78-0.82)	0.80 (0.78-0.82)	0.78 (0.76-0.81)	0.79 (0.77-0.81)	0.78 (0.76-0.80)	0.79 (0.78-0.81)	0.78 (0.76-0.80)	0.79 (0.77-0.80)
	PeriOp	0.84 (0.82-0.86)	0.84 (0.82-0.85)	0.84 (0.82-0.86)	0.84 (0.82-0.86)	0.83 (0.82-0.85)	0.84 (0.83-0.86)	0.83 (0.81-0.85)	0.84 (0.82-0.85)
Venous thromboembolism	PreOp	0.80 (0.77-0.83)	0.82 (0.79-0.84)	0.77 (0.73-0.80)	0.80 (0.77-0.83)	0.77 (0.74-0.81)	0.81 (0.78-0.84)	0.78 (0.74-0.81)	0.80 (0.77-0.83)
	PeriOp	0.84 (0.81-0.87)	0.82 (0.80-0.85)	0.82 (0.79-0.86)	0.82 (0.80-0.85)	0.82 (0.79-0.85)	0.82 (0.79-0.84)	0.81 (0.78-0.85)	0.82 (0.79-0.84)
Prolonged mechanical ventilation	PreOp	0.85 (0.82-0.88)	0.84 (0.82-0.86)	0.84 (0.81-0.87)	0.83 (0.81-0.85)	0.83 (0.80-0.86)	0.83 (0.81-0.85)	0.83 (0.80-0.86)	0.82 (0.80-0.84)
	PeriOp	0.85 (0.82-0.88)	0.87 (0.85-0.89)	0.86 (0.83-0.88)	0.86 (0.85-0.88)	0.85 (0.82-0.88)	0.86 (0.84-0.88)	0.85 (0.82-0.88)	0.86 (0.84-0.88)
Neurological complications, including delirium	PreOp	0.87 (0.85-0.88)	0.83 (0.82-0.84)[a]	0.85 (0.84-0.87)	0.83 (0.82-0.84)	0.85 (0.84-0.87)	0.83 (0.81-0.84)[a]	0.85 (0.83-0.86)	0.82 (0.81-0.84)
	PeriOp	0.87 (0.85-0.88)	0.84 (0.82-0.85)[a]	0.86 (0.84-0.88)	0.83 (0.82-0.85)	0.86 (0.84-0.87)	0.83 (0.82-0.84)[a]	0.86 (0.84-0.87)	0.83 (0.82-0.85)
Wound complications	PreOp	0.71 (0.68-0.73)	0.72 (0.71-0.74)	0.69 (0.67-0.72)	0.72 (0.70-0.74)	0.69 (0.67-0.72)	0.72 (0.70-0.73)	0.70 (0.67-0.72)	0.72 (0.70-0.74)
	PeriOp	0.73 (0.70-0.75)	0.74 (0.72-0.76)	0.74 (0.71-0.76)	0.73 (0.72-0.75)	0.72 (0.69-0.75)	0.74 (0.72-0.75)	0.72 (0.69-0.75)	0.74 (0.72-0.76)
	PreOp	0.81	0.78	0.80	0.78	0.80	0.78	0.80	0.78

		CL	CL	SCAFFOLD	SCAFFOLD	FedAvg	FedAvg	FedProx	FedProx
**Outcome**	**Period**	**African American**	**Non-African American**	**African American**	**Non-African American**	**African American**	**Non-African American**	**African American**	**Non-African American**
Acute kidney injury		(0.79-0.83)	(0.77-0.80)	(0.78-0.82)	(0.76-0.79)	(0.78-0.82)	(0.77-0.80)	(0.78-0.82)	(0.77-0.80)
	PeriOp	0.82 (0.80-0.84)	0.80 (0.78-0.81)	0.83 (0.81-0.84)	0.80 (0.78-0.81)	0.82 (0.80-0.83)	0.80 (0.78-0.81)	0.81 (0.79-0.83)	0.79 (0.78-0.81)
In-hospital mortality	PreOp	0.95 (0.93-0.96)	0.90 (0.88-0.93)[a]	0.94 (0.91-0.96)	0.88 (0.85-0.91)[a]	0.93 (0.90-0.96)	0.88 (0.84-0.91)[a]	0.92 (0.89-0.95)	0.88 (0.85-0.91)
	PeriOp	0.97 (0.95-0.98)	0.91 (0.88-0.94)[a]	0.97 (0.96-0.98)	0.89 (0.86-0.92)[a]	0.97 (0.95-0.98)	0.89 (0.86-0.92)[a]	0.97 (0.96-0.98)	0.90 (0.87-0.93)[a]

[a] p<=0.05 comparing African American to Non-African American patients for each model.
Abbreviation: CL, central learning; ICU, intensive care unit; PreOp, preoperative; PeriOp, perioperative.

SDC Table 8. Subgroup analysis of AUROC with 95% confidence interval for federated learning models based on age in the UFH GNV cohort

		CL	CL	SCAFFOLD	SCAFFOLD	FedAvg	FedAvg	FedProx	FedProx
**Outcome**	**Period**	**Age <= 65**	**Age > 65**	**Age <= 65**	**Age > 65**	**Age <= 65**	**Age > 65**	**Age <= 65**	**Age > 65**
Prolonged ICU stay	PreOp	0.90 (0.90-0.90)	0.88 (0.88-0.89)[a]	0.91 (0.90-0.91)	0.89 (0.89-0.90)[a]	0.90 (0.90-0.91)	0.89 (0.89-0.90)[a]	0.91 (0.90-0.91)	0.89 (0.89-0.90)[a]
	PeriOp	0.92 (0.91-0.92)	0.91 (0.90-0.91)[a]	0.92 (0.92-0.93)	0.91 (0.91-0.92)[a]	0.92 (0.92-0.93)	0.91 (0.91-0.92)[a]	0.92 (0.92-0.93)	0.91 (0.91-0.92)
Sepsis	PreOp	0.88 (0.88-0.89)	0.87 (0.86-0.88)[a]	0.88 (0.88-0.89)	0.87 (0.86-0.88)[a]	0.88 (0.87-0.89)	0.87 (0.86-0.88)	0.88 (0.88-0.89)	0.87 (0.86-0.88)
	PeriOp	0.89 (0.88-0.90)	0.88 (0.87-0.89)[a]	0.89 (0.88-0.90)	0.88 (0.87-0.89)	0.89 (0.89-0.90)	0.88 (0.87-0.89)[a]	0.89 (0.89-0.90)	0.88 (0.87-0.89)
Cardiovascular complication	PreOp	0.83 (0.82-0.83)	0.79 (0.78-0.80)[a]	0.83 (0.82-0.84)	0.79 (0.78-0.80)[a]	0.82 (0.82-0.83)	0.79 (0.78-0.80)[a]	0.83 (0.82-0.84)	0.79 (0.79-0.80)[a]
	PeriOp	0.86 (0.85-0.86)	0.83 (0.82-0.84)[a]	0.86 (0.85-0.87)	0.83 (0.82-0.84)[a]	0.86 (0.85-0.87)	0.83 (0.82-0.84)[a]	0.86 (0.85-0.87)	0.83 (0.82-0.84)[a]
Venous thromboembolism	PreOp	0.85 (0.83-0.86)	0.80 (0.79-0.82)[a]	0.85 (0.84-0.86)	0.80 (0.79-0.82)[a]	0.84 (0.83-0.85)	0.79 (0.78-0.81)[a]	0.85 (0.84-0.86)	0.81 (0.79-0.82)[a]
	PeriOp	0.85 (0.84-0.86)	0.81 (0.79-0.82)[a]	0.85 (0.84-0.86)	0.82 (0.80-0.83)[a]	0.85 (0.84-0.86)	0.82 (0.80-0.83)[a]	0.85 (0.84-0.86)	0.81 (0.80-0.83)[a]
Prolonged mechanical ventilation	PreOp	0.91 (0.90-0.92)	0.88 (0.87-0.89)[a]	0.91 (0.91-0.92)	0.88 (0.87-0.89)[a]	0.91 (0.90-0.92)	0.88 (0.87-0.89)[a]	0.92 (0.91-0.92)	0.88 (0.87-0.89)[a]
	PeriOp	0.92 (0.91-0.93)	0.90 (0.90-0.91)[a]	0.92 (0.92-0.93)	0.90 (0.89-0.91)[a]	0.93 (0.92-0.94)	0.91 (0.90-0.92)[a]	0.93 (0.92-0.93)	0.91 (0.90-0.92)[a]
Neurological complications, including delirium	PreOp	0.87 (0.86-0.88)	0.82 (0.81-0.82)[a]	0.87 (0.86-0.88)	0.82 (0.82-0.83)[a]	0.87 (0.86-0.88)	0.82 (0.81-0.83)[a]	0.87 (0.86-0.88)	0.82 (0.81-0.83)[a]
	PeriOp	0.87 (0.86-0.87)	0.82 (0.81-0.83)[a]	0.87 (0.87-0.88)	0.83 (0.82-0.84)[a]	0.88 (0.87-0.88)	0.83 (0.82-0.84)[a]	0.87 (0.87-0.88)	0.83 (0.82-0.84)[a]
Wound complications	PreOp	0.79 (0.78-0.79)	0.80 (0.79-0.81)	0.79 (0.78-0.80)	0.81 (0.80-0.82)[a]	0.78 (0.77-0.79)	0.80 (0.79-0.81)[a]	0.79 (0.78-0.80)	0.81 (0.80-0.82)[a]
	PeriOp	0.79 (0.78-0.80)	0.81 (0.80-0.82)[a]	0.80 (0.79-0.81)	0.82 (0.81-0.83)[a]	0.80 (0.79-0.80)	0.82 (0.81-0.83)[a]	0.80 (0.79-0.81)	0.82 (0.81-0.83)[a]
Acute kidney injury	PreOp	0.83 (0.82-0.84)	0.79 (0.78-0.80)[a]	0.83 (0.82-0.84)	0.80 (0.79-0.81)[a]	0.83 (0.82-0.84)	0.79 (0.78-0.80)[a]	0.83 (0.83-0.84)	0.80 (0.79-0.81)[a]
	PeriOp	0.84	0.80	0.84	0.81	0.84	0.81	0.84	0.81

		CL	CL	SCAFFOLD	SCAFFOLD	FedAvg	FedAvg	FedProx	FedProx
**Outcome**	**Period**	**Age <= 65**	**Age > 65**	**Age <= 65**	**Age > 65**	**Age <= 65**	**Age > 65**	**Age <= 65**	**Age > 65**
		(0.83-0.85)	(0.79-0.80)[a]	(0.84-0.85)	(0.80-0.82)[a]	(0.83-0.85)	(0.80-0.81)[a]	(0.83-0.85)	(0.80-0.82)[a]
In-hospital mortality	PreOp	0.92 (0.91-0.94)	0.86 (0.84-0.88)[a]	0.92 (0.90-0.93)	0.84 (0.82-0.87)[a]	0.91 (0.89-0.93)	0.85 (0.82-0.87)[a]	0.92 (0.91-0.94)	0.86 (0.84-0.88)[a]
	PeriOp	0.92 (0.91-0.93)	0.87 (0.86-0.89)[a]	0.92 (0.91-0.94)	0.88 (0.86-0.89)[a]	0.93 (0.92-0.94)	0.88 (0.86-0.90)[a]	0.93 (0.91-0.94)	0.88 (0.86-0.90)[a]

[a] p<=0.05 comparing patients with age > 65 to those with age <= 65 for each model.

Abbreviation: CL, central learning; ICU, intensive care unit; PreOp, preoperative; PeriOp, perioperative.

SDC Table 9. Subgroup analysis of AUROC with 95% confidence interval for federated learning models based on age in the UFH JAX cohort

		CL		SCAFFOLD		FedAvg		FedProx	
**Outcome**	**Period**	**Age <= 65**	**Age > 65**	**Age <= 65**	**Age > 65**	**Age <= 65**	**Age > 65**	**Age <= 65**	**Age > 65**
Prolonged ICU stay	PreOp	0.89 (0.89-0.90)	0.89 (0.87-0.90)	0.87 (0.86-0.87)	0.85 (0.84-0.87)	0.87 (0.86-0.88)	0.87 (0.85-0.88)	0.87 (0.86-0.87)	0.86 (0.84-0.87)
	PeriOp	0.91 (0.90-0.92)	0.89 (0.87-0.90)[a]	0.90 (0.89-0.91)	0.88 (0.86-0.89)[a]	0.89 (0.88-0.90)	0.86 (0.85-0.88)[a]	0.89 (0.88-0.90)	0.87 (0.85-0.88)[a]
Sepsis	PreOp	0.89 (0.87-0.90)	0.90 (0.88-0.92)	0.88 (0.87-0.89)	0.89 (0.87-0.91)	0.88 (0.86-0.89)	0.89 (0.87-0.91)	0.88 (0.86-0.89)	0.89 (0.87-0.91)
	PeriOp	0.90 (0.88-0.91)	0.90 (0.88-0.92)	0.89 (0.88-0.90)	0.90 (0.88-0.92)	0.89 (0.88-0.90)	0.89 (0.87-0.91)	0.89 (0.87-0.90)	0.90 (0.87-0.92)
Cardiovascular complication	PreOp	0.81 (0.80-0.83)	0.75 (0.72-0.77)[a]	0.80 (0.78-0.81)	0.75 (0.72-0.78)[a]	0.80 (0.78-0.81)	0.74 (0.72-0.77)[a]	0.79 (0.78-0.81)	0.74 (0.72-0.77)[a]
	PeriOp	0.85 (0.84-0.87)	0.79 (0.76-0.81)[a]	0.85 (0.83-0.86)	0.80 (0.77-0.83)[a]	0.85 (0.83-0.86)	0.80 (0.77-0.82)[a]	0.85 (0.83-0.86)	0.80 (0.77-0.82)[a]
Venous thromboembolism	PreOp	0.82 (0.80-0.84)	0.79 (0.75-0.83)	0.80 (0.77-0.82)	0.76 (0.72-0.80)	0.81 (0.78-0.83)	0.77 (0.72-0.80)	0.80 (0.78-0.83)	0.76 (0.72-0.80)
	PeriOp	0.83 (0.81-0.85)	0.82 (0.77-0.85)	0.83 (0.80-0.85)	0.82 (0.78-0.85)	0.82 (0.80-0.85)	0.81 (0.76-0.85)	0.82 (0.80-0.84)	0.80 (0.76-0.84)
Prolonged mechanical ventilation	PreOp	0.85 (0.83-0.87)	0.82 (0.79-0.85)	0.84 (0.82-0.86)	0.83 (0.80-0.86)	0.83 (0.82-0.85)	0.83 (0.80-0.86)	0.82 (0.81-0.84)	0.81 (0.78-0.84)
	PeriOp	0.87 (0.85-0.88)	0.85 (0.81-0.88)	0.86 (0.84-0.88)	0.86 (0.83-0.90)	0.85 (0.84-0.87)	0.86 (0.83-0.89)	0.86 (0.84-0.87)	0.86 (0.83-0.89)
Neurological complications, including delirium	PreOp	0.86 (0.85-0.87)	0.78 (0.76-0.80)[a]	0.85 (0.84-0.87)	0.78 (0.75-0.80)[a]	0.85 (0.84-0.86)	0.78 (0.76-0.80)[a]	0.84 (0.83-0.86)	0.78 (0.76-0.80)[a]
	PeriOp	0.86 (0.85-0.88)	0.79 (0.76-0.81)[a]	0.86 (0.84-0.87)	0.79 (0.76-0.81)[a]	0.85 (0.84-0.87)	0.78 (0.76-0.80)[a]	0.86 (0.84-0.87)	0.78 (0.76-0.80)[a]
Wound complications	PreOp	0.72 (0.71-0.74)	0.70 (0.68-0.74)	0.71 (0.70-0.73)	0.70 (0.67-0.73)	0.71 (0.70-0.73)	0.69 (0.66-0.72)	0.71 (0.70-0.73)	0.70 (0.68-0.73)
	PeriOp	0.74 (0.72-0.75)	0.73 (0.70-0.76)	0.74 (0.72-0.75)	0.73 (0.70-0.76)	0.74 (0.72-0.75)	0.72 (0.70-0.75)	0.74 (0.72-0.75)	0.73 (0.70-0.76)
Acute kidney injury	PreOp	0.80 (0.79-0.81)	0.75 (0.73-0.77)[a]	0.79 (0.78-0.81)	0.76 (0.73-0.78)[a]	0.80 (0.78-0.81)	0.74 (0.72-0.77)[a]	0.79 (0.78-0.81)	0.75 (0.73-0.77)[a]
	PeriOp	0.82	0.76	0.81	0.77	0.81	0.76	0.81	0.76

		CL		SCAFFOLD		FedAvg		FedProx	
**Outcome**	**Period**	**Age <= 65**	**Age > 65**	**Age <= 65**	**Age > 65**	**Age <= 65**	**Age > 65**	**Age <= 65**	**Age > 65**
		(0.80-0.83)	(0.74-0.79)[a]	(0.80-0.83)	(0.75-0.79)[a]	(0.80-0.82)	(0.74-0.79)[a]	(0.79-0.82)	(0.74-0.78)[a]
In-hospital mortality	PreOp	0.94 (0.92-0.96)	0.86 (0.82-0.90)[a]	0.91 (0.88-0.94)	0.84 (0.80-0.88)[a]	0.91 (0.88-0.94)	0.84 (0.79-0.88)[a]	0.90 (0.87-0.94)	0.84 (0.80-0.89)
	PeriOp	0.95 (0.93-0.97)	0.88 (0.83-0.92)[a]	0.93 (0.90-0.96)	0.89 (0.84-0.92)	0.93 (0.90-0.96)	0.87 (0.83-0.91)[a]	0.94 (0.91-0.96)	0.88 (0.84-0.92)[a]

[a] p<=0.05 comparing patients with age > 65 to those with age <= 65 for each model.
Abbreviation: CL, central learning; ICU, intensive care unit; PreOp, preoperative; PeriOp, perioperative.

SDC Table 10. Subgroup analysis of AUROC with 95% confidence interval across models (central learning and SCAFFOLD models) based on surgery type in the UFH GNV cohort

		CL					SCAFFOLD				
**Outcome**	**Period**	**Cardiothoracic surgery**	**Noncardiac general surgery**	**Neurological Surgery**	**Specialty Surgery**	**Other Surgery**	**Cardiothoracic surgery**	**Noncardiac general surgery**	**Neurological Surgery**	**Specialty Surgery**	**Other Surgery**
Prolonged ICU stay	PreOp	0.80 (0.78-0.81)	0.87 (0.86-0.88)[a]	0.84 (0.83-0.85)[ab]	0.88 (0.87-0.89)[ac]	0.89 (0.87-0.91)[ac]	0.84 (0.83-0.86)	0.88 (0.87-0.89)[a]	0.85 (0.84-0.86)[b]	0.89 (0.88-0.89)[ac]	0.89 (0.87-0.92)[ac]
	PeriOp	0.86 (0.85-0.87)	0.89 (0.89-0.90)[a]	0.86 (0.85-0.87)[b]	0.91 (0.90-0.92)[abc]	0.90 (0.88-0.92)[ac]	0.88 (0.87-0.89)	0.90 (0.90-0.91)[a]	0.86 (0.85-0.87)[ab]	0.92 (0.91-0.92)[abc]	0.91 (0.89-0.93)[ac]
Sepsis	PreOp	0.83 (0.82-0.85)	0.86 (0.85-0.87)	0.85 (0.83-0.87)	0.89 (0.88-0.90)[abc]	0.83 (0.78-0.88)[d]	0.84 (0.81-0.85)	0.86 (0.85-0.87)	0.85 (0.82-0.87)	0.89 (0.88-0.90)[abc]	0.84 (0.80-0.88)
	PeriOp	0.86 (0.84-0.87)	0.87 (0.86-0.88)	0.86 (0.83-0.88)	0.90 (0.89-0.91)[abc]	0.83 (0.79-0.88)[d]	0.85 (0.84-0.87)	0.87 (0.86-0.88)	0.84 (0.82-0.87)	0.90 (0.89-0.91)[abc]	0.84 (0.79-0.88)[d]
Cardiovascular complication	PreOp	0.70 (0.68-0.72)	0.79 (0.78-0.80)[a]	0.77 (0.75-0.79)[a]	0.77 (0.76-0.79)[a]	0.80 (0.75-0.84)[a]	0.70 (0.68-0.71)	0.79 (0.78-0.80)[a]	0.78 (0.76-0.79)[a]	0.78 (0.76-0.79)[a]	0.81 (0.77-0.85)[a]
	PeriOp	0.76 (0.74-0.77)	0.83 (0.82-0.84)[a]	0.79 (0.78-0.81)[ab]	0.83 (0.81-0.84)[ac]	0.82 (0.79-0.86)[a]	0.75 (0.74-0.77)	0.84 (0.83-0.85)[a]	0.79 (0.77-0.81)[ab]	0.83 (0.82-0.84)[ac]	0.82 (0.79-0.86)[a]
Venous thromboembolism	PreOp	0.79 (0.77-0.81)	0.79 (0.78-0.80)	0.81 (0.79-0.84)	0.82 (0.79-0.84)	0.81 (0.74-0.86)	0.80 (0.78-0.82)	0.79 (0.78-0.81)	0.82 (0.79-0.84)	0.82 (0.79-0.84)	0.80 (0.74-0.85)
	PeriOp	0.81 (0.79-0.83)	0.79 (0.78-0.80)	0.82 (0.80-0.84)	0.81 (0.79-0.83)	0.80 (0.74-0.85)	0.82 (0.80-0.84)	0.81 (0.79-0.82)	0.81 (0.79-0.84)	0.82 (0.80-0.84)	0.80 (0.74-0.86)
Prolonged mechanical ventilation	PreOp	0.82 (0.80-0.84)	0.89 (0.88-0.90)[a]	0.89 (0.88-0.91)[a]	0.90 (0.88-0.91)[a]	0.86 (0.79-0.92)	0.82 (0.80-0.84)	0.89 (0.88-0.90)[a]	0.90 (0.88-0.91)[a]	0.91 (0.90-0.92)[a]	0.86 (0.80-0.92)
	PeriOp	0.86 (0.84-0.88)	0.90 (0.89-0.91)[a]	0.91 (0.89-0.92)[a]	0.91 (0.90-0.93)[a]	0.86 (0.79-0.93)	0.86 (0.84-0.88)	0.91 (0.90-0.92)[a]	0.90 (0.89-0.92)[a]	0.92 (0.91-0.94)[a]	0.87 (0.80-0.94)
Neurological complications	PreOp	0.73 (0.71-0.75)	0.84 (0.83-0.85)[a]	0.81 (0.80-0.82)[ab]	0.80 (0.79-0.81)[ab]	0.78 (0.74-0.82)[b]	0.72 (0.70-0.74)	0.84 (0.83-0.85)[a]	0.82 (0.81-0.83)[ab]	0.81 (0.80-0.82)[ab]	0.77 (0.74-0.81)[b]
	PeriOp	0.75 (0.73-0.77)	0.85 (0.84-0.85)[a]	0.82 (0.81-0.83)[ab]	0.80 (0.79-0.81)[ab]	0.77 (0.73-0.81)[b]	0.74 (0.72-0.76)	0.85 (0.84-0.86)[a]	0.82 (0.81-0.84)[ab]	0.82 (0.80-0.83)[ab]	0.78 (0.74-0.81)[bc]
Wound complications	PreOp	0.68 (0.66-0.70)	0.74 (0.73-0.75)[a]	0.75 (0.73-0.77)[a]	0.85 (0.84-0.85)[abc]	0.70 (0.67-0.74)[d]	0.69 (0.67-0.71)	0.75 (0.74-0.76)[a]	0.75 (0.74-0.77)[a]	0.84 (0.83-0.85)[abc]	0.73 (0.70-0.76)[d]
	PeriOp	0.68 (0.66-0.70)	0.76 (0.75-0.77)[a]	0.77 (0.75-0.78)[a]	0.85 (0.84-0.86)[abc]	0.72 (0.68-0.75)[cd]	0.70 (0.68-0.72)	0.76 (0.75-0.77)[a]	0.77 (0.76-0.79)[a]	0.86 (0.85-0.87)[abc]	0.74 (0.71-0.77)[d]
Acute kidney injury	PreOp	0.79 (0.78-0.81)	0.79 (0.78-0.80)	0.78 (0.76-0.79)	0.81 (0.80-0.82)[c]	0.83 (0.79-0.86)[c]	0.79 (0.78-0.81)	0.80 (0.79-0.81)	0.78 (0.76-0.80)	0.82 (0.81-0.83)[ac]	0.84 (0.80-0.87)[ac]
	PeriOp	0.80 (0.78-0.81)	0.80 (0.79-0.81)	0.78 (0.76-0.79)	0.82 (0.81-0.83)[c]	0.84 (0.81-0.87)[abc]	0.82 (0.80-0.83)	0.81 (0.80-0.82)	0.78 (0.76-0.80)[a]	0.83 (0.82-0.84)[bc]	0.85 (0.81-0.88)[c]

		CL					SCAFFOLD				
**Outcome**	**Period**	**Cardiothoracic surgery**	**Noncardiac general surgery**	**Neurological Surgery**	**Specialty Surgery**	**Other Surgery**	**Cardiothoracic surgery**	**Noncardiac general surgery**	**Neurological Surgery**	**Specialty Surgery**	**Other Surgery**
In-hospital mortality	PreOp	0.78 (0.74-0.83)	0.89 (0.87-0.90)[a]	0.87 (0.84-0.90)[a]	0.91 (0.87-0.94)[a]	0.97 (0.96-0.99)[abcd]	0.76 (0.71-0.81)	0.87 (0.85-0.89)[a]	0.88 (0.85-0.90)[a]	0.91 (0.88-0.94)[a]	0.97 (0.96-0.98)[abcd]
	PeriOp	0.80 (0.75-0.85)	0.89 (0.87-0.91)[a]	0.89 (0.86-0.91)[a]	0.92 (0.90-0.94)[a]	0.96 (0.93-0.98)[abcd]	0.81 (0.76-0.85)	0.89 (0.87-0.91)[a]	0.89 (0.86-0.91)[a]	0.93 (0.89-0.96)[a]	0.97 (0.96-0.99)[abcd]

Noncardiac general surgery includes general gastrointestinal surgery, general oncology surgery, general colorectal surgery, vascular surgery, acute care and burn surgery, and transplant surgery.
Specialty surgery includes urological surgery, orthopedics surgery, gynecologic surgery, and ear nose throat surgery.
Other surgery includes ophthalmology and plastic surgery.
[a] $p<=0.05$ compared to patients underwent cardiothoracic surgery for each model.
[b] $p<=0.05$ compared to patients underwent noncardiac general surgery for each model.
[c] $p<=0.05$ compared to patients underwent neurologic surgery for each model.
[d] $p<=0.05$ compared to patients underwent specialty surgery for each model.
Abbreviation: CL, central learning; ICU, intensive care unit; PreOp, preoperative; PeriOp, perioperative.

SDC Table 11. Subgroup analysis of AUROC with 95% confidence interval across models (FedAvg and FedProx models) based on surgery type in the UFH GNV cohort

		FedAvg					FedProx				
**Outcome**	**Period**	**Cardiothoracic surgery**	**Noncardiac general surgery**	**Neurologic Surgery**	**Specialty Surgery**	**Other Surgery**	**Cardiothoracic surgery**	**Noncardiac general surgery**	**Neurologic Surgery**	**Specialty Surgery**	**Other Surgery**
Prolonged ICU stay	PreOp	0.84 (0.83-0.86)	0.87(0.86-0.88)[a]	0.85 (0.84-0.86)[b]	0.88 (0.88-0.89)[ac]	0.90 (0.88-0.92)[ac]	0.85 (0.84-0.87)	0.88 (0.87-0.88)[a]	0.85 (0.84-0.86)[b]	0.89 (0.88-0.90)[ac]	0.89 (0.87-0.92)[ac]
	PeriOp	0.89 (0.88-0.90)	0.90(0.89-0.91)	0.87 (0.86-0.88)[ab]	0.91 (0.91-0.92)[abc]	0.92 (0.90-0.94)[abc]	0.88 (0.87-0.89)	0.90 (0.89-0.91)[a]	0.86 (0.85-0.87)[ab]	0.92 (0.91-0.92)[abc]	0.92 (0.90-0.94)[ac]
Sepsis	PreOp	0.83 (0.81-0.85)	0.86(0.85-0.87)[a]	0.85 (0.82-0.87)	0.88 (0.87-0.90)[abc]	0.83 (0.78-0.87)	0.84 (0.82-0.86)	0.86 (0.85-0.87)	0.85 (0.83-0.87)	0.89 (0.88-0.90)[abc]	0.83 (0.79-0.87)[d]
	PeriOp	0.86 (0.84-0.88)	0.87 (0.86-0.88)	0.85 (0.83-0.88)	0.90 (0.89-0.91)[abc]	0.82 (0.78-0.87)[d]	0.86 (0.84-0.87)	0.87 (0.86-0.88)	0.85 (0.83-0.87)	0.90 (0.89-0.91)[abc]	0.83 (0.78-0.87)[d]
Cardiovascular complication	PreOp	0.68 (0.66-0.70)	0.80 (0.79-0.81)[a]	0.78 (0.76-0.80)[a]	0.77 (0.76-0.79)[a]	0.79 (0.75-0.83)[a]	0.70 (0.68-0.72)	0.80 (0.78-0.81)[a]	0.78 (0.76-0.79)[a]	0.79 (0.78-0.80)[a]	0.79 (0.75-0.83)[a]
	PeriOp	0.76 (0.74-0.78)	0.84 (0.83-0.85)[a]	0.80 (0.78-0.81)[ab]	0.83 (0.82-0.85)[ac]	0.83 (0.79-0.86)[a]	0.76 (0.74-0.77)	0.84 (0.83-0.85)[a]	0.80 (0.78-0.82)[ab]	0.83 (0.82-0.85)[ac]	0.83 (0.79-0.86)[a]
Venous thromboembolism	PreOp	0.80 (0.78-0.82)	0.78 (0.77-0.80)	0.81 (0.78-0.83)	0.80 (0.77-0.82)	0.78 (0.71-0.84)	0.79 (0.77-0.81)	0.80 (0.79-0.81)	0.82 (0.79-0.84)	0.82 (0.79-0.84)	0.82 (0.77-0.87)
	PeriOp	0.82 (0.80-0.84)	0.80 (0.79-0.81)	0.82 (0.80-0.84)	0.81 (0.79-0.84)	0.81 (0.75-0.86)	0.81 (0.79-0.83)	0.81 (0.79-0.82)	0.82 (0.80-0.84)	0.82 (0.80-0.84)	0.83 (0.77-0.87)
Prolonged MV	PreOp	0.82 (0.80-0.84)	0.89 (0.88-0.90)[a]	0.90 (0.88-0.91)[a]	0.90 (0.88-0.91)[a]	0.85 (0.79-0.91)	0.82 (0.81-0.84)	0.90 (0.88-0.90)[a]	0.90 (0.89-0.91)[a]	0.90 (0.89-0.92)[a]	0.86 (0.80-0.92)
	PeriOp	0.87 (0.86-0.89)	0.91 (0.90-0.92)[a]	0.91 (0.90-0.93)[a]	0.92 (0.90-0.93)[a]	0.87 (0.80-0.93)	0.86 (0.85-0.88)	0.91 (0.90-0.92)[a]	0.91 (0.90-0.92)[a]	0.92 (0.90-0.93)[a]	0.88 (0.82-0.93)
Neurological complications	PreOp	0.74 (0.72-0.76)	0.84 (0.83-0.85)[a]	0.81 (0.79-0.82)[ab]	0.81 (0.80-0.82)[ab]	0.78 (0.74-0.82)[b]	0.73 (0.71-0.75)	0.84 (0.83-0.85)[a]	0.82 (0.81-0.83)[ab]	0.80 (0.79-0.82)[ab]	0.79 (0.75-0.83)[ab]
	PeriOp	0.75 (0.73-0.76)	0.85 (0.85-0.86)[a]	0.82 (0.81-0.83)[ab]	0.82 (0.80-0.83)[ab]	0.78 (0.74-0.82)[b]	0.75 (0.73-0.76)	0.85 (0.84-0.86)[a]	0.82 (0.81-0.83)[ab]	0.81 (0.80-0.82)[ab]	0.79 (0.75-0.83)[b]
Wound complications	PreOp	0.66 (0.63-0.68)	0.74 (0.73-0.75)[a]	0.74 (0.73-0.76)[a]	0.84 (0.83-0.85)[abc]	0.72 (0.68-0.75)[ad]	0.69 (0.67-0.71)	0.75 (0.74-0.76)[a]	0.75 (0.73-0.77)[a]	0.85 (0.84-0.86)[abc]	0.73 (0.70-0.76)[d]
	PeriOp	0.70 (0.68-0.72)	0.76 (0.75-0.77)[a]	0.77 (0.75-0.79)[a]	0.86 (0.85-0.87)[abc]	0.75 (0.72-0.79)[ad]	0.71 (0.69-0.73)	0.76 (0.75-0.77)[a]	0.77 (0.75-0.79)[a]	0.86 (0.85-0.87)[abc]	0.75 (0.71-0.78)[d]
Acute kidney injury	PreOp	0.80 (0.78-0.82)	0.79 (0.78-0.81)	0.78 (0.76-0.80)	0.81 (0.80-0.82)[c]	0.83 (0.80-0.87)[c]	0.80 (0.78-0.81)	0.80 (0.79-0.81)	0.78 (0.76-0.80)	0.82 (0.81-0.83)[c]	0.84 (0.80-0.87)[bc]
	PeriOp	0.82 (0.80-0.84)	0.81 (0.80-0.82)	0.78 (0.76-0.80)[ab]	0.82 (0.81-0.83)[c]	0.84 (0.80-0.87)[c]	0.81 (0.80-0.83)	0.81 (0.80-0.82)	0.78 (0.76-0.80)[a]	0.82 (0.81-0.83)[c]	0.83 (0.80-0.86)[c]

		FedAvg					FedProx				
**Outcome**	**Period**	**Cardiothoracic surgery**	**Noncardiac general surgery**	**Neurologic Surgery**	**Specialty Surgery**	**Other Surgery**	**Cardiothoracic surgery**	**Noncardiac general surgery**	**Neurologic Surgery**	**Specialty Surgery**	**Other Surgery**
In-hospital mortality	PreOp	0.77 (0.73-0.82)	0.87 (0.85-0.89)[a]	0.86 (0.83-0.89)[a]	0.90 (0.87-0.93)[a]	0.98 (0.97-0.99)[abcd]	0.78 (0.73-0.83)	0.89 (0.86-0.91)[a]	0.87 (0.84-0.90)[a]	0.94 (0.92-0.96)[abc]	0.98 (0.97-0.99)[abcd]
	PeriOp	0.81 (0.77-0.86)	0.90 (0.87-0.91)[a]	0.90 (0.87-0.92)[a]	0.94 (0.92-0.96)[abc]	0.97 (0.96-0.99)[abcd]	0.80 (0.76-0.85)	0.89 (0.87-0.91)[a]	0.89 (0.86-0.91)[a]	0.93 (0.91-0.95)[ab]	0.96 (0.94-0.98)[abc]

Noncardiac general surgery includes general gastrointestinal surgery, general oncology surgery, general colorectal surgery, vascular surgery, acute care and burn surgery, and transplant surgery.
Specialty surgery includes urological surgery, orthopedics surgery, gynecologic surgery, and ear nose throat surgery.
Other surgery includes ophthalmology and plastic surgery.
[a] p<=0.05 compared to patients underwent cardiothoracic surgery for each model.
[b] p<=0.05 compared to patients underwent noncardiac general surgery for each model.
[c] p<=0.05 compared to patients underwent neurologic surgery for each model.
[d] p<=0.05 compared to patients underwent specialty surgery for each model.
Abbreviation: ICU, intensive care unit; PreOp, preoperative; PeriOp, perioperative.

SDC Table 12. Subgroup analysis of AUROC with 95% confidence interval across models (central learning and SCAFFOLD models) based on surgery type in the UFH JAX cohort

		CL					SCAFFOLD				
**Outcome**	**Period**	**Cardiothoracic surgery**	**Noncardiac general surgery**	**Neurologic Surgery**	**Specialty Surgery**	**Other Surgery**	**Cardiothoracic surgery**	**Noncardiac general surgery**	**Neurologic Surgery**	**Specialty Surgery**	**Other Surgery**
Prolonged ICU stay	PreOp	0.77 (0.71-0.83)	0.88 (0.87-0.89)[a]	0.71 (0.68-0.74)[b]	0.84 (0.82-0.86)[abc]	0.84 (0.81-0.87)[bc]	0.74 (0.68-0.80)	0.84 (0.83-0.86)[a]	0.72 (0.69-0.75)[b]	0.83 (0.81-0.85)[ac]	0.80 (0.77-0.83)[bc]
	PeriOp	0.83 (0.78-0.87)	0.90 (0.89-0.92)[a]	0.75 (0.72-0.77)[ab]	0.87 (0.85-0.88)[bc]	0.86 (0.84-0.89)[bc]	0.84 (0.79-0.88)	0.89 (0.87-0.90)	0.74 (0.71-0.76)[ab]	0.87 (0.85-0.88)[c]	0.85 (0.83-0.88)[c]
Sepsis	PreOp	0.87 (0.80-0.94)	0.85 (0.83-0.87)	0.85 (0.80-0.90)	0.89 (0.87-0.91)	0.86 (0.83-0.88)	0.87 (0.80-0.94)	0.84 (0.82-0.86)	0.86 (0.81-0.90)	0.89 (0.86-0.91)[b]	0.85 (0.83-0.88)
	PeriOp	0.87 (0.80-0.94)	0.87 (0.85-0.89)	0.84 (0.78-0.90)	0.91 (0.89-0.93)[b]	0.87 (0.84-0.89)[d]	0.89 (0.83-0.94)	0.86 (0.84-0.88)	0.86 (0.81-0.90)	0.90 (0.87-0.92)[b]	0.85 (0.82-0.87)[d]
Cardiovascular complication	PreOp	0.65 (0.59-0.71)	0.81 (0.79-0.83)[a]	0.70 (0.65-0.75)[b]	0.77 (0.74-0.79)[ac]	0.83 (0.79-0.86)[acd]	0.67 (0.61-0.72)	0.79 (0.77-0.81)[a]	0.70 (0.64-0.76)[b]	0.77 (0.74-0.79)[a]	0.80 (0.76-0.84)[ac]
	PeriOp	0.70 (0.64-0.74)	0.85 (0.83-0.87)[a]	0.74 (0.69-0.79)[b]	0.82 (0.80-0.85)[ac]	0.86 (0.83-0.89)[ac]	0.69 (0.64-0.74)	0.85 (0.83-0.87)[a]	0.72 (0.67-0.78)[b]	0.83 (0.81-0.85)[ac]	0.86 (0.83-0.89)[ac]
Venous thromboembolism	PreOp	0.68 (0.56-0.80)	0.80 (0.77-0.83)	0.63 (0.54-0.71)[b]	0.86 (0.82-0.89)[abc]	0.74 (0.67-0.81)[d]	0.70 (0.58-0.81)	0.77 (0.74-0.81)	0.60 (0.51-0.68)[b]	0.85 (0.81-0.88)[abc]	0.73 (0.65-0.79)[cd]
	PeriOp	0.66 (0.54-0.78)	0.83 (0.80-0.86)[a]	0.68 (0.60-0.75)[b]	0.87 (0.83-0.91)[ac]	0.74 (0.66-0.81)[d]	0.76 (0.68-0.84)	0.82 (0.79-0.85)	0.67 (0.59-0.76)[b]	0.87 (0.82-0.90)[c]	0.72 (0.65-0.79)[bd]
Prolonged mechanical ventilation	PreOp	0.71 (0.65-0.77)	0.85 (0.82-0.88)[a]	0.89 (0.84-0.92)[a]	0.83 (0.80-0.86)[a]	0.84 (0.78-0.89)[a]	0.71 (0.64-0.78)	0.82 (0.79-0.85)[a]	0.89 (0.86-0.93)[ab]	0.84 (0.81-0.87)[ac]	0.80 (0.75-0.85)[c]
	PeriOp	0.71 (0.63-0.78)	0.88 (0.85-0.90)[a]	0.90 (0.86-0.93)[a]	0.86 (0.83-0.89)[a]	0.86 (0.82-0.91)[a]	0.75 (0.68-0.81)	0.87 (0.84-0.90)[a]	0.91 (0.87-0.94)[a]	0.84 (0.81-0.87)[ac]	0.87 (0.83-0.92)[a]
Neurological complications	PreOp	0.65 (0.58-0.72)	0.87 (0.85-0.89)[a]	0.74 (0.70-0.77)[b]	0.82 (0.80-0.84)[abc]	0.84 (0.81-0.87)[ac]	0.67 (0.60-0.74)	0.85 (0.83-0.87)[a]	0.75 (0.72-0.78)[b]	0.82 (0.79-0.84)[abc]	0.84 (0.81-0.87)[ac]
	PeriOp	0.69 (0.63-0.76)	0.87 (0.86-0.89)[a]	0.77 (0.74-0.80)[b]	0.82 (0.80-0.84)[abc]	0.83 (0.80-0.86)[abc]	0.65 (0.59-0.72)	0.86 (0.84-0.88)[a]	0.77 (0.74-0.79)[ab]	0.82 (0.80-0.84)[abc]	0.84 (0.81-0.86)[ac]
Wound complications	PreOp	0.62 (0.54-0.70)	0.73 (0.70-0.75)[a]	0.64 (0.60-0.68)[b]	0.75 (0.72-0.77)[ac]	0.62 (0.58-0.66)[bd]	0.58 (0.51-0.66)	0.71 (0.68-0.73)[a]	0.64 (0.59-0.68)[b]	0.75 (0.72-0.77)[ac]	0.64 (0.60-0.68)[bd]
	PeriOp	0.67 (0.59-0.73)	0.76 (0.74-0.78)[a]	0.66 (0.61-0.70)[b]	0.76 (0.74-0.78)[ac]	0.65 (0.61-0.69)[bd]	0.63 (0.55-0.70)	0.74 (0.71-0.76)[a]	0.64 (0.60-0.68)[b]	0.77 (0.74-0.79)[ac]	0.65 (0.61-0.70)[bd]
Acute kidney injury	PreOp	0.62 (0.57-0.68)	0.80 (0.78-0.82)[a]	0.77 (0.73-0.82)[a]	0.79 (0.77-0.81)[a]	0.79 (0.76-0.82)[a]	0.63 (0.57-0.68)	0.79 (0.77-0.82)[a]	0.77 (0.72-0.82)[a]	0.78 (0.75-0.80)[a]	0.79 (0.76-0.82)[a]
	PeriOp	0.67 (0.62-0.72)	0.82 (0.80-0.84)[a]	0.78 (0.74-0.82)[a]	0.80 (0.78-0.82)[a]	0.80 (0.77-0.83)[a]	0.69 (0.63-0.74)	0.82 (0.79-0.84)[a]	0.77 (0.73-0.82)[a]	0.80 (0.78-0.82)[a]	0.80 (0.77-0.83)[a]

		CL					SCAFFOLD				
**Outcome**	**Period**	**Cardiothoracic surgery**	**Noncardiac general surgery**	**Neurologic Surgery**	**Specialty Surgery**	**Other Surgery**	**Cardiothoracic surgery**	**Noncardiac general surgery**	**Neurologic Surgery**	**Specialty Surgery**	**Other Surgery**
In-hospital mortality	PreOp	0.73 (0.50-0.92)	0.90 (0.87-0.94)	0.95 (0.92-0.98)	0.94 (0.92-0.96)	0.92 (0.86-0.97)	0.67 (0.43-0.89)	0.86 (0.81-0.91)	0.93 (0.89-0.97)	0.94 (0.91-0.96)[b]	0.91 (0.86-0.95)
	PeriOp	0.80 (0.61-0.95)	0.95 (0.92-0.97)	0.96 (0.94-0.98)	0.93 (0.90-0.96)	0.89 (0.79-0.95)	0.83 (0.63-0.96)	0.93 (0.88-0.97)	0.91 (0.85-0.97)	0.93 (0.90-0.95)	0.93 (0.88-0.97)

Noncardiac general surgery includes general gastrointestinal surgery, general oncology surgery, general colorectal surgery, vascular surgery, acute care and burn surgery, and transplant surgery.
Specialty surgery includes urological surgery, orthopedics surgery, gynecologic surgery, and ear nose throat surgery.
Other surgery includes ophthalmology and plastic surgery.
[a] $p<=0.05$ compared to patients underwent cardiothoracic surgery for each model.
[b] $p<=0.05$ compared to patients underwent noncardiac general surgery for each model.
[c] $p<=0.05$ compared to patients underwent neurologic surgery for each model.
[d] $p<=0.05$ compared to patients underwent specialty surgery for each model.
Abbreviation: CL, central learning; ICU, intensive care unit; PreOp, preoperative; PeriOp, perioperative.

SDC Table 13. Subgroup analysis of AUROC with 95% confidence interval across models (FedAvg and FedProx models) based on surgery type in the UFH JAX cohort

		FedAvg					FedProx				
**Outcome**	**Period**	**Cardiothoracic surgery**	**Noncardiac general surgery**	**Neurologic Surgery**	**Specialty Surgery**	**Other Surgery**	**Cardiothoracic surgery**	**Noncardiac general surgery**	**Neurologic Surgery**	**Specialty Surgery**	**Other Surgery**
Prolonged ICU stay	PreOp	0.73 (0.67-0.80)	0.85 (0.84-0.86)[a]	0.73 (0.71-0.76)[b]	0.82 (0.80-0.84)[ac]	0.80 (0.77-0.83)[bc]	0.77 (0.71-0.84)	0.85 (0.83-0.86)	0.71 (0.68-0.74)[b]	0.82 (0.80-0.84)[c]	0.80 (0.77-0.83)[bc]
	PeriOp	0.87 (0.83-0.90)	0.88 (0.87-0.89)	0.74 (0.72-0.77)[ab]	0.83 (0.81-0.85)[bc]	0.85 (0.82-0.88)[c]	0.85 (0.81-0.89)	0.89 (0.87-0.90)	0.73 (0.70-0.76)[ab]	0.84 (0.82-0.86)[bc]	0.85 (0.82-0.88)[c]
Sepsis	PreOp	0.86 (0.78-0.93)	0.85 (0.82-0.87)	0.86 (0.82-0.90)	0.88 (0.85-0.90)	0.84 (0.82-0.87)	0.87 (0.79-0.94)	0.84 (0.82-0.86)	0.84 (0.78-0.89)	0.88 (0.85-0.90)	0.85 (0.83-0.88)
	PeriOp	0.87 (0.79-0.94)	0.86 (0.84-0.88)	0.84 (0.78-0.89)	0.89 (0.87-0.91)	0.86 (0.84-0.89)	0.88 (0.81-0.94)	0.85 (0.83-0.88)	0.83 (0.77-0.89)	0.89 (0.87-0.91)[b]	0.85 (0.83-0.88)[d]
Cardiovascular complication	PreOp	0.68 (0.63-0.74)	0.78 (0.76-0.80)[a]	0.74 (0.69-0.79)	0.77 (0.74-0.79)[a]	0.80 (0.76-0.83)[a]	0.68 (0.62-0.74)	0.78 (0.75-0.80)[a]	0.70 (0.65-0.76)	0.77 (0.74-0.79)[a]	0.79 (0.75-0.83)[ac]
	PeriOp	0.69 (0.64-0.73)	0.85 (0.83-0.87)[a]	0.74 (0.69-0.79)[b]	0.83 (0.81-0.85)[ac]	0.86 (0.83-0.89)[ac]	0.69 (0.63-0.73)	0.85 (0.83-0.87)[a]	0.73 (0.67-0.78)[b]	0.83 (0.81-0.85)[ac]	0.84 (0.81-0.88)[ac]
Venous thromboembolism	PreOp	0.69 (0.58-0.80)	0.78 (0.75-0.82)	0.62 (0.54-0.70)[b]	0.84 (0.80-0.88)[ac]	0.74 (0.67-0.80)[d]	0.69 (0.58-0.80)	0.78 (0.74-0.81)	0.62 (0.54-0.71)[b]	0.84 (0.80-0.88)[abc]	0.74 (0.66-0.80)[d]
	PeriOp	0.64 (0.52-0.76)	0.81 (0.78-0.84)[a]	0.70 (0.62-0.77)[b]	0.86 (0.82-0.89)[ac]	0.74 (0.67-0.80)[d]	0.67 (0.54-0.78)	0.80 (0.77-0.83)	0.68 (0.61-0.75)[b]	0.86 (0.82-0.89)[abc]	0.75 (0.69-0.81)[d]
Prolonged mechanical ventilation	PreOp	0.70 (0.63-0.76)	0.85 (0.82-0.87)[a]	0.90 (0.86-0.93)[ab]	0.82 (0.79-0.85)[ac]	0.80 (0.74-0.85)[c]	0.73 (0.65-0.79)	0.83 (0.80-0.86)[a]	0.88 (0.84-0.92)[a]	0.81 (0.78-0.84)[c]	0.79 (0.74-0.84)[c]
	PeriOp	0.72 (0.65-0.79)	0.87 (0.84-0.89)[a]	0.90 (0.86-0.93)[a]	0.85 (0.82-0.87)[a]	0.86 (0.81-0.90)[a]	0.71 (0.65-0.77)	0.87 (0.84-0.89)[a]	0.89 (0.86-0.93)[a]	0.85 (0.82-0.88)[a]	0.86 (0.81-0.91)[a]
Neurological complications	PreOp	0.68 (0.61-0.74)	0.86 (0.84-0.88)[a]	0.73 (0.70-0.76)[b]	0.81 (0.79-0.83)[abc]	0.83 (0.80-0.86)[ac]	0.69 (0.62-0.75)	0.85 (0.83-0.87)[a]	0.74 (0.71-0.77)[b]	0.81 (0.78-0.83)[abc]	0.82 (0.79-0.85)[ac]
	PeriOp	0.65 (0.58-0.71)	0.87 (0.85-0.89)[a]	0.75 (0.72-0.78)[ab]	0.82 (0.79-0.84)[abc]	0.84 (0.81-0.87)[ac]	0.65 (0.58-0.71)	0.87 (0.85-0.88)[a]	0.76 (0.73-0.79)[ab]	0.82 (0.80-0.84)[abc]	0.84 (0.81-0.86)[ac]
Wound complications	PreOp	0.60 (0.53-0.67)	0.71 (0.68-0.73)[a]	0.64 (0.59-0.68)[b]	0.74 (0.72-0.76)[ac]	0.66 (0.62-0.70)[d]	0.61 (0.54-0.68)	0.71 (0.69-0.74)[a]	0.63 (0.59-0.67)[b]	0.74 (0.72-0.76)[ac]	0.64 (0.59-0.68)[bd]
	PeriOp	0.62 (0.54-0.68)	0.74 (0.72-0.77)[a]	0.66 (0.62-0.70)[b]	0.75 (0.73-0.78)[ac]	0.68 (0.64-0.72)[bd]	0.62 (0.55-0.68)	0.74 (0.72-0.76)[a]	0.67 (0.63-0.71)[b]	0.75 (0.73-0.77)[ac]	0.67 (0.63-0.71)[bd]
Acute kidney injury	PreOp	0.63 (0.57-0.68)	0.80 (0.78-0.82)[a]	0.75 (0.71-0.80)[a]	0.79 (0.76-0.81)[a]	0.79 (0.76-0.82)[a]	0.63 (0.58-0.68)	0.79 (0.77-0.82)[a]	0.77 (0.72-0.82)[a]	0.79 (0.76-0.81)[a]	0.78 (0.75-0.81)[a]
	PeriOp	0.69 (0.63-0.74)	0.82 (0.80-0.84)[a]	0.78 (0.74-0.82)[a]	0.80 (0.78-0.82)[a]	0.80 (0.77-0.83)[a]	0.67 (0.62-0.72)	0.82 (0.80-0.84)[a]	0.76 (0.72-0.81)[a]	0.80 (0.78-0.82)[a]	0.79 (0.76-0.82)[a]

		FedAvg					FedProx				
**Outcome**	**Period**	**Cardiothoracic surgery**	**Noncardiac general surgery**	**Neurologic Surgery**	**Specialty Surgery**	**Other Surgery**	**Cardiothoracic surgery**	**Noncardiac general surgery**	**Neurologic Surgery**	**Specialty Surgery**	**Other Surgery**
In-hospital mortality	PreOp	0.73 (0.48-0.94)	0.86 (0.81-0.91)	0.92 (0.89-0.96)	0.92 (0.89-0.95)	0.92 (0.88-0.96)	0.78 (0.52-0.98)	0.85 (0.79-0.90)	0.93 (0.89-0.96)[b]	0.93 (0.91-0.96)[b]	0.91 (0.87-0.95)
	PeriOp	0.76 (0.50-0.94)	0.92 (0.87-0.96)	0.95 (0.92-0.98)	0.93 (0.91-0.96)	0.92 (0.88-0.96)	0.76 (0.51-0.96)	0.92 (0.88-0.96)	0.95 (0.93-0.98)	0.94 (0.92-0.96)	0.92 (0.89-0.96)

Noncardiac general surgery includes general gastrointestinal surgery, general oncology surgery, general colorectal surgery, vascular surgery, acute care and burn surgery, and transplant surgery.
Specialty surgery includes urological surgery, orthopedics surgery, gynecologic surgery, and ear nose throat surgery.
Other surgery includes ophthalmology and plastic surgery.
[a] p<=0.05 compared to patients underwent cardiothoracic surgery for each model.
[b] p<=0.05 compared to patients underwent noncardiac general surgery for each model.
[c] p<=0.05 compared to patients underwent neurologic surgery for each model.
[d] p<=0.05 compared to patients underwent specialty surgery for each model.
Abbreviation: ICU, intensive care unit; PreOp, preoperative; PeriOp, perioperative.

SDC Table 14. Comparison of AUROC for federated learning preoperative models with varied and equal training sample sizes

	UFH GNV		UFH JAX	
**Outcome**	**SCAFFOLD model trained using raw data size**	**SCAFFOLD model trained using equal size, mean (SD)**	**SCAFFOLD model trained using raw data size**	**SCAFFOLD model trained using equal size, mean (SD)**
Prolonged ICU stay	0.90	0.89 (0.002)	0.87	0.89 (0.003)
Sepsis	0.88	0.87 (0.002)	0.88	0.88 (0.002)
Cardiovascular complication	0.82	0.81 (0.002)	0.79	0.79 (0.002)
Venous thromboembolism	0.83	0.81 (0.004)	0.79	0.80 (0.005)
Prolonged mechanical ventilation	0.90	0.89 (0.005)	0.84	0.84 (0.004)
Neurological complications, including delirium	0.85	0.84 (0.002)	0.84	0.84 (0.003)
Wound complications	0.80	0.78 (0.003)	0.71	0.71 (0.002)
Acute kidney injury	0.82	0.81 (0.001)	0.79	0.79 (0.003)
In-hospital mortality	0.89	0.88 (0.011)	0.90	0.91 (0.002)

Abbreviation: ICU, intensive care unit; SD, standard deviation.
